# A COSMIN Systematic Review of Sexual Health Literacy Self-Report Measures for Adolescents

**DOI:** 10.1007/s10508-025-03142-1

**Published:** 2025-06-06

**Authors:** Marlene Muehlmann, Katja Nieradt, Samuel Tomczyk

**Affiliations:** 1 Department Health and Prevention, Institute of Psychology, University of Greifswald, Robert-Blum-Str. 13, 17489 Greifswald, Germany; 2 German Center of Child and Adolescent Health, partner site Greifswald/Rostock, Greifswald, Germany

**Keywords:** Sexual health, Health literacy, COSMIN, Adolescent health

## Abstract

Sexual health literacy (SHL) is crucial for adolescent health. Measurement consistency aids in comparing studies and informing interventions. This systematic review identifies available self-report outcome measurement instruments (OMI) assessing SHL in adolescents, explores the aspects of SHL they cover, and assess their psychometric quality. Guided by the COSMIN guidelines, a systematic search of 9 databases was conducted, focusing on studies between 2002 and 18 April 2023. Inclusion criteria encompassed studies developing and validating OMIs assessing SHL in adolescents aged 10–19 years, with a focus on self-report OMIs developed since 2002—the year the World Health Organization (WHO, 2006) defined sexual health as it is defined today (WHO, 2006). A narrative synthesis assessed the conceptual fit of identified instruments with SHL dimensions. The quality of the development studies was evaluated using COSMIN criteria. The review was preregistered in PROSPERO (ID: CRD42022303682). Out of 18,637 records, 83 studies examining 68 different OMIs were included. OMIs varied in their coverage of SHL dimensions, with appraisal and application of sexual health information most frequently addressed. The quality of OMI development was generally inadequate or doubtful, with deficiencies noted in involvement of the target population and piloting processes. Findings underscore the need for standardized, well-developed OMIs to measure SHL in adolescents. Despite availability, OMIs’ quality and alignment with SHL are insufficient. There is a clear need for improvement in OMI development processes, including greater involvement of the target population and more rigorous piloting procedures. Additionally, developing a comprehensive SHL measure will enhance comparability and promote adolescent sexual health understanding and interventions.

**Supplementary Information:**

The online version contains supplementary material available at 10.1007/s10508-025-03142-1.

## Introduction

To date, programs in the field of sexuality education and the promotion of adolescent sexual health (WHO, 2001) have been evaluated in various ways and typically lacking comprehensiveness (Downing et al., 2011; Guse et al., 2012; Picot et al., 2012; Santa Maria et al., 2015; Widman et al., 2019; Wight & Fullerton, 2013). Although comprehensive sexuality education is considered a promising approach (International Planned Parenthood Federation [IPPF], 2010; Matloobi et al., 2022; WHO Regional Office for Europe & German Federal Centre for Health Education [BZgA], 2011), evaluations of programs often rely on traditional and very specific indicators such as later sexual debut, lower STI prevalence, number of adolescent pregnancies, or sexual partners (e.g., Santa Maria et al., 2015; Widman et al., 2019; Wight & Fullerton, 2013). Firstly, there is no core set of outcomes which makes it difficult to compare findings across studies. Secondly, typical indicators neglect the contemporary understanding of SH, where efficacy measures of programs should not only consider absent functional disorders and health risk avoidance but also promoting individuals’ well-being (Palmer et al., 2017, 2019; WHO, 2006). To this end, competence-oriented indicators such as sexual health literacy (SHL) have received increased attention in recent years (e.g., Alzate et al., 2020; Bober & Chevalier, 2021), which could complement specific measurements. Low SHL can lead to maladaptive sexual health attitudes and behaviors among adolescents (Zhamantayev et al., 2023), while high SHL can promote SH outcomes, e.g., the utilization of healthcare services (Väisänen et al., 2021). While previous studies often focus on some aspects of SHL (Martin, 2017), this comprehensive concept has rarely been studied (Martin, 2017), and it is thus unclear how it is best captured in adolescents.

SHL can be defined in line with the understanding of Sexual Health as proposed by the WHO (2006), and conceptualized similar to common health literacy concepts (Sørensen et al., 2012): Individual SHL is a specific kind of health literacy (Martin, 2017) comprising knowledge, beliefs, attitudes, motivations, competences, and skills to (1) access, (2) understand, (3) appraise, and (4) apply sexual health information in order to make judgments, take decisions, and negotiate in everyday life concerning (1) being ill, (2) being at risk, and (3) staying healthy to maintain or improve quality of life (Martin, 2017; Sørensen et al., 2012). Individual SHL is also influenced by contextual factors, including aspects of the healthcare system and social factors (Martin, 2017). Table 1 shows the HLS-EU health literacy matrix (Sørensen et al., 2013) in adapted form for sexual health literacy.

**Table 1:** The Sexual Health Literacy matrix

	(1) Access/Obtain information relevant to SH	(2) Understand information relevant to SH	(3) Process/Appraise information relevant to SH	(4) Apply/Use information relevant to SH	(5) Context factors relevant to SHL
(1) Healthcare	Ability to access information on medical and clinical issues (e.g. abortion, pregnancy)	Ability to understand medical information and derive meaning	Ability to interpret and evaluate medical information	Ability to make informed decisions on medical issues	Context factors that can influence the ability to make informed decisions on medical issues
(2) Disease prevention	Ability to access information on risk factors for SH (e.g. condom use, contraception, pearl index)	Ability to understand information on risk factors and derive meaning	Ability to interpret and evaluate information on risk factors for SH	Ability to make informed decisions on risk factors for SH	Context factors that can influence the ability to make informed decisions on risk factors for SH
(3) Health promotion	Ability to update oneself on determinants of SH in the social and physical environment (e.g. conversation with parents on sex and relationships)	Ability to understand information on determinants of SH in the social and physical environment and derive meaning	Ability to interpret and evaluate information on SH determinants in the social and physical environment	Ability to make informed decisions on SH determinants in the social and physical environment	Context factors that can influence the ability to make informed decisions on SH determinants in the social and physical environment

Based on Sørensen et al. (2013). SH = Sexual Health, SHL = Sexual Health Literacy

This matrix provides a comprehensive framework of SHL to synthesize findings, identify research and practice gaps, and improve sexual health promotion and education globally to improve public sexual health (Goodwin, 2021; Haruna et al., 2018; Lee-Foon et al., 2022). It can also inform the development, selection, and evaluation of psychometrically sound, validated measurements of SHL (Church et al., 2023; Väisänen et al., 2021). The aim of the review was to summarize and evaluate outcome measurement instruments (OMIs) that are suitable for measuring SHL. Therefore, the following research questions were addressed:

1. What self-report OMIs are available to assess SHL in adolescents?
2. Which aspects of SHL are considered by the existing OMIs?
3. What is the psychometric quality of existing OMIs?

## Method

The systematic review was guided by the recommendations of the COnsensus-based Standards for the selection of health Measurement INstruments (COSMIN) initiative (Mokkink et al. 2018a, Mokkink et al., 2016; Prinsen et al., 2016). It was preregistered on PROSPERO (ID: CRD42022303682; Muehlmann et al., 2022).

### Systematic Search

The following databases were searched from 2002 to the search date (18 April 2023): Medline/PubMed, PsycINFO, PSYNDEX, PsycArticles, Web of Science, Educational Resources Information Center (ERIC), Cochrane Library, EMBASE, Applied Social Sciences Index and Abstracts (ASSIA). Key author’s publication were searched by hand. References of included articles were screened manually.

The search strategy contained several components (see Supplement 1): the respective outcome–SHL–consistent with our working definition of SHL (Martin, 2017; Sørensen et al., 2012; e.g., sexual, literacy), the population of interest (e.g., adolescent, youth), and the type of the instrument (e.g., validation, comparative study; Jansma & de Vries, 2017; Terwee et al., 2009). Components were combined using Boolean Operators (OR, AND)*.*

Eligibile studies regard the construct of interest–based on the given definition of SHL–and the population of interest—adolescents between 10 and 19 years (WHO, 2001). The aim of the studies were the evaluation of one or more measurement properties, the development of an OMI, or the evaluation of the interpretability of the OMIs of interest (Prinsen et al., 2018). Furthermore, studies were included if the OMIs had been developed, updated or adapted since 2002—the year the WHO defined sexual health as it is defined today (WHO, 2006). Studies in English and German language that were peer-reviewed publications were included. Studies were excluded if they did not measure SHL-aspects, did not represent the target sample, concerned self-report OMIs or only used the respective OMIs as an outcome measurement instrument (Prinsen et al., 2018). Studies concerning OMIs that have not been updated or adapted since 2002 were excluded as well as studies in languages other than English or German due to limited language capabilities, and publication types such as comments, interviews, or newspaper articles (following Terwee et al., 2009; see also Supplement 2).

One author collected and synthesized all data using Citavi 6.17 and Excel 2019. Duplicates were removed via Citavi 6.17. The same author screened titles and abstracts for eligibility. All following procedures of full text screening, inclusion, data extraction, and quality rating were conducted by two independent raters. Differences were resolved through discussion until consensus was reached. Details regarding studies were extracted, including authors, year, and country and grouped into (1) OMI development including a design and a piloting phase, and (2) OMI validation studies based on the COSMIN framework (see Results section Included Studies for details). Information on study populations was collected divided into OMI design, piloting, and validation phase. This included capturing sample description, sample size, as well as age, and gender distribution. Wherever possible, missing sample characteristics (e.g., mean age of the study population) were calculated using available information. Additionally, information on OMIs was collected, covering language, topics, and subscales.

### Outcome Measurement Instruments

A narrative synthesis regarding the identified OMIs was performed. On item level it was rated whether the OMIs addressed the dimensions of SHL according to the adapted SHL Matrix (Sørensen et al., 2013; see Supplement 3 for sample items used as anchors). The conceptual fit of the analysed OMIs was rated on three levels: the dimension is not considered by the OMI (−), is considered by the OMI (+), or is covered comprehensively due to the relevant health area (++). If the full item list was not available, study authors were contacted. If the item list was then not provided, the fit was assessed using the OMI description in the study (see Results section Included OMIs).

### Risk of Bias

The COSMIN Risk of Bias checklist was used for quality assessment (Mokkink et al., 2018a). According to COMSIN, the most relevant psychometric property is content validity (Mokkink, Prinsen et al., 2018b; Terwee et al., 2018a, 2018b). In order to assess it, the quality of the OMI development is first evaluated using the COSMIN Box 3 (Terwee et al., 2018a, 2018b). This is followed by the evaluation of the quality of the content validity studies applying the COSMIN Box 2, before the content validity itself is evaluated. The COSMIN Box 3 to Box 10 evaluate (1) structural validity, (2) internal consistency, (3) cross-cultural validity, (4) reliability, (5) measurement error, (6) criterion validity, (7) construct validity, and (8) responsiveness (Prinsen et al., 2018).

Since initial analysis revealed that many OMIs were insufficient as early as in the development phase, the present review took a two-step approach. First, all included OMIs were examined regarding the quality of their development (development studies). Achievable ratings were very good (V), adequate (A), doubtful (D), and inadequate (I). Only OMIs with an A or V rating in the development phase were subsequently analyzed with regard to further psychometric properties (Prinsen et al., 2018).

## Results

### Search Process

**Fig. 1:**
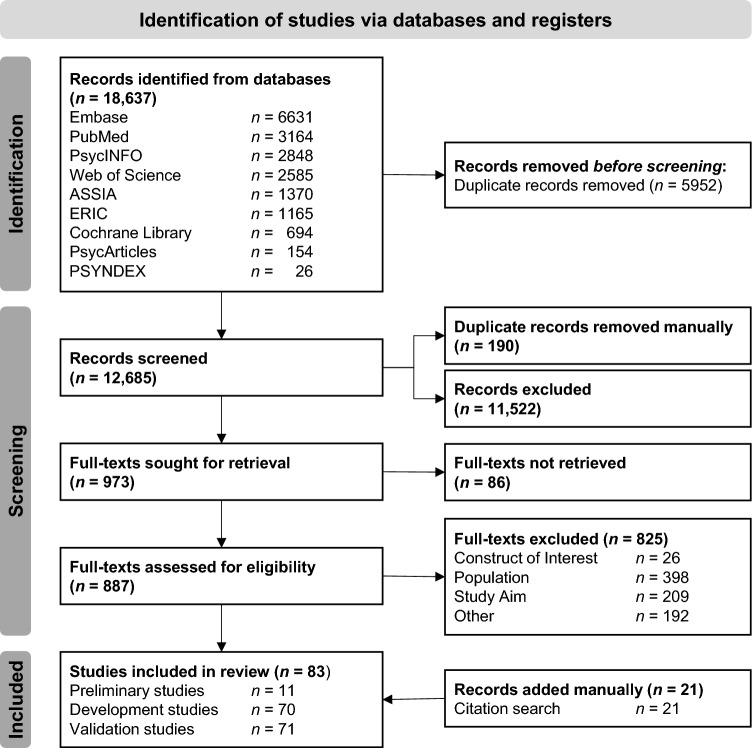
PRISMA Flow Chart of the systematic review of selfreported outcome measurement instruments of sexual health literacy in adolescents, based on the PRISMA 2020 statement (Page et al., 2021)

A total of 18,637 records was identified. After excluding duplicates, 12,685 titles and abstracts were screened with respect to the inclusion and exclusion criteria. Two independent raters examined 887 full texts and included 62 studies for the final analyses. Through manual search, 21 additional studies were identified, resulting in a total of 83 studies being included. Details are shown in the PRISMA flow chart in Fig. 1 (see Supplement 4 for details).

### Included Studies

Of the 83 included studies, 11 (13%) reported exclusively preliminary investigations such as qualitative studies within the target group without additional psychometric examination. The development of the OMI, encompassing the design (*k* = 35, 42%) and piloting (*k* = 42, 50%) process, was addressed in 70 studies. The validation of the OMI was the focus of 71 (86%) studies. An overview of the studies is presented in Supplement 5.

Most studies were from North America (*k* = 49, 59%), particularly the USA (*k* = 45, 54%), and Canada (*k* = 5). Other studies were conducted in Europe (*k* = 22, 27%), particularly Spain (*k* = 7, 8%), and Portugal (*k* = 3, 4%). Some studies originated from Asia (*k* = 10, 12%). Most African studies (*k* = 9, 11%) were conducted in cooperation with other countries. Few studies from Australia (*k* = 5, 6%), and Latin America (*k* = 2, 2%) could be identified. Studies from the years 2003–2023 were considered, with a publication peak (*k* = 8, 10%) in 2012 (see Supplement 6).

Details on study populations divided into OMI design, OMI piloting, and OMI validation study were captured regarding sample description, sample size, age, and gender distribution. Sample sizes of the development studies, combining both design and pilot phases, varied between *N* = 3 and *N* = 755, while the sample sizes of validation studies ranged from *N* = 62 to *N* = 15,782. Development studies were conducted with participants aged between 12 and 30 years, with average scores (*M* or *Mdn*) of 14–18·9 years. The OMIs were validated with participants between the ages of 10 and 32 years, with an average (*M* or *Mdn*) of 12·5–19·9 years. In some cases, the age of the participants was not specified but their grade level. These studies were conducted in grades 5–12 (typically span ages 10–18, depending on the education system). The gender distribution in combined samples ranged from 45% female to 92% female in development studies. In validation studies, female representation ranged from 44 to 81%. Studies on some OMIs (*n* = 16, 24%) concerned exclusively female adolescents throughout all research phases, a few (*n* = 2, 3%) focused on male participants only. In addition to binary gender information, for 6 (7%) studies additional genders were reported.

Missingness of descriptive sample data was much higher for development (= design and pilot phases) than validation studies (see Supplement 6). In fact, comprehensive information on analyzed samples (i.e., sample size, age range, age, and gender distribution) could only be identified with certainty for 10 (13%) studies within the design and pilot phases.

### Included Outcome Measurement Instruments

The included 83 studies examined 68 different OMIs that were assessed regarding the quality of their development and their fit with SHL dimensions. The *inter-rater reliability* of both independent raters was Kappa = 0.660 for the content assessments, and weighted Kappa = 0.793 for the development quality ratings. According to Landis and Koch (1977) both of these values can be considered as substantial agreement.

#### Topics of the Outcome Measurement Instruments

The content of the OMIs was assessed at item level where possible. In 11 (13%) studies, the description of the OMIs in the original study was used, as the questionnaire itself was not available. Table 2 shows the topics and subscales of the respective OMIs as well as the SHL dimensions (access, understand, appraise, apply, context factors) that were considered.

**Table 2:** Topis of the Outcome Measurement Instruments

OMI	OMI-Languages	Subscales/Topic	Access			Understand			Appraise			Apply			Context		
			HC	DP	HP	HC	DP	HP	HC	DP	HP	HC	DP	HP	HC	DP	HP
Sexual Health Questionnaire (Acharya et al., 2016)	(Nepali)	(1) SH norms and beliefs, (2) Sources of SH information, (3) SH knowledge/understanding, (4) SH awareness level	+	+	+	+		+		+	+						
Measures of Adolescents’ Attitudes about Sexual Relationship Rights (Berglas et al., 2017)	(English)	(1) Sex Refusal, (2) Sex Engagement								+	+						
The Adolescent Masculinity Ideology in Relationships Scale (AMIRS; Chu et al., 2005)	English, Spanish	*Attitudes and beliefs about what constitutes appropriate behaviors for males within The contexts of their interpersonal relationships (unidimensional)*									+						
Belief-Based Reproductive Health Questionnaire (BBRHQ; Darabi et al., 2018)	(Farsi)	(1) Reproductive health Knowledge, (2) Attitudes towards reproductive health, (3) Subjective Norm, (4) Behavioral Intention, (5) Perceived parental control, (6) Perceived behavioral control, (7) Reproductive health Behavior			+				+	+	+		+	+ +		+	+
Untitled (Deardorff et al., 2008)	English, Spanish	*Sexual values scales* (1) Sexual Talk as Disrespectful, (2) Female Virginity as Important, (3) Comfort with Sexual Communication, (4) Sexual Comfort, (5) Sexual Self-Acceptance, (6) Satisfaction of Sexual Needs as Important						+			+		+	+			
The Married Adolescent Women's Sexual and Reproductive Health Needs Assessment Questionnaire (MAWSRHNAQ; Ghiasi et al., 2022)	(Farsi)	(1) Need to improve married adolescent women's sexual quality of life, (2) Need to promote married adolescent women's SRH self-care, (3) Need to improve married adolescent women's SRH self-efficacy, (4) Need to increase married adolescent women's SRH knowledge, (5) Need to increase husband's involvement in married adolescent woman's SRH, (6) Need to improve the performance of health care providers, (7) Need to strengthen the family support to married adolescent women, (8) Need to improve family involvement in SRH education of married adolescent women, (9) Need to provide specic premarital counseling to married adolescent women	+	+	+	+	+					+	+	+	+	+	+
Scale of Myths about Sexuality (Escala de Mitos sobre la Sexualidad, EMS; Guerra et al., 2019)	Spanish	(1) Intolerance, (2) Romantic love, (3) Sexist Myths, (4) Generational Myths, (5) Contraception, (6) Pregnancy				( +)	( +)				( +)						
Untitled (Jerman et al., 2015)	English	*SH Measures* Behavioral Subscales: (1) Romantic and sexual behaviors, (2) SH services history SH Subscales: (3) SH knowledge, (4) SH service knowledge, (5) Sexual orientation acceptance, (6) Condom-use values, (7) Partner sexual communication, (8) Parent sexual communiation, (9) Protection self-efficacy, (10) Condom-use intentions (steady partner & casual partner)	+	+	+	+	+			+	+		+	+	+	+	
The Sexual Self-Concept Inventory (SSCI; O'Sullivan et al., 2006)	English, Spanish	(1) Sexual Arousability, (2) Sexual Agency, (3) Negative Sexual Affect									+			+			
The Homophobic Bullying Scale (Prati, 2012)	Italian	(1) Homophobic aggressive behavior (witness perspective), (2) Homophobic aggressive behavior (bully perspective), (3) Homophobic victimization (victim perspective)												+			+
Adolescent Health Attitude and Behavior Survey (AHABS; Reininger et al., 2003)	(English)	Attitudes Towards Adolescent Sexual Behavior Subscales: (1)Perceived Sexual Norms, (2) Perceived Birth Control Use, (3) Perceptions of Others Sexual Involvement Youth Assets Subscales: (4) Youth's Accountability to Parents and Other Adults, (5) Youth Empowerment, (6) Quantity of Other Adult Support, (7) Youth's Empathetic Relationship, (8) Youth's Perceived Support by Parents and Other Adults, (9) Youth's Perceived School Support, (10) Self Peer Values Regarding Risk Behaviors			+					+	+		+				+
The Parent-Adolescent Communication Scale (PACS; Sales et al., 2008)	(English)	*Adolescents’ self-reported frequency of communicating about sexually related topics with their parents (unidimensional)*		+	+												
The Worry About Sexual Outcomes (WASO) Scale (Sales et al., 2009)	(English)	(1) STI/HIV worry, (2) Pregnancy worry	+	+													
Cyberdating Q_A (Sánchez et al., 2015)	Spanish	(1) Online Control, (2) Online Jealousy, (3) Online Intrusive Behavior, (4) Online Intimacy, (5) Emotional Communication Strategies, (6) Cyberdating Practices												+			
The Gender Roles and Male Provision Expectations (GRMPE) scale (Kyegombe et al., 2020; Stoebenau et al., 2023)	Luganda	(1) Male authority, (2) Male Sexual decision-making, (3) Women’s sexual agency, (4) Provision and love									+ +						
The Gender Climate Scale (GCS; Ullman, 2014; Ullman et al., 2023)	(English)	(1) School acceptance and support of gender and sexual diversity, (2) Reinforcement of Traditional Gender Difference, (3) Freedom of subject selection, (4) Freedom of Appearance Expression, (5) Inclusive Health and Physical Education Curriculum, (6) Academic fairness, (7) Popularity based on gender norms															+ +
The Sexual and Reproductive Empowerment Scale for Adolescents and Young Adults (Fefferman & Upadhyay, 2018; Upadhyay & Lipkovich, 2020; Upadhyay et al., 2021)	(English)	(1) Comfort talking with partner, (2) Choice of partners, (3) Marriage and children, (4) Parental support, (5) Sexual safety, (6) Self-love, (7) Sense of future, (8) Sexual pleasure										+	+	+			+
Untitled (Edwards et al., 2015, 2018)	(English)	*Relationship Abuse and Sexual Assault Prevention Program Effectiveness Among Youth* (1) Media Literacy, (2) Rape Myths, (3) Bystander Intentions, (4) Bystander Readiness-Denial, (5) Barriers and Facilitators of Bystander Action, (6) Perceptions of Peer Helping, (7) Perceptions of Personnel Helping, (8) Victim Empathy			+						+		+	+		+	+
The Measure of Adolescent Heterosocial Competence (MAHC; Grover et al., 2005, 2007)	English	*General communication, initiation, friendships, dating, sexual situations, drugs and alcohol, Harassment/abusive situations, school situations, work situations*											( +)	( +)			
Untitled (Apidechkul, 2019)	(Thai)	*Sexual behaviors, history of STIs, experience in receiving information regarding STIs and courses of information, knowledge and attitude of risk of STIs*		( +)			( +)			( +)		( +)	( +)				
Sexual Self-Concept (SSC) Scale (Biney, 2016)	(Ga, Twi, English)	(1) Sexual control, (2) Sexual intrepidness, (3) Sexual enthusiasm, (4) Sexual readiness			+						+			+			+
Untitled (Fisher et al., 2018)	(English)	*Barriers to HIV Prevention Services for Transgender Youth* (1) Gender and sexual minority Stigma, (2) Confidentiality Concerns, (3) Gender and sexual minority-SH Information	+	+													
Relational Entitlement and Proprietariness Scale (REPS; Hannawa et al., 2006)	(English)	(1) Behavioral Control, (2) Social Control, (3) Face Threat Reactivity, (4) Information Control									+			+			
The Adolescent Attitudes to Abortion Scale (AAA Scale; Hendriks et al., 2012; Skinner et al., 2008, 2009)	(English)	*Attitudes and intentions relating to abortion (unidimensional)*							+			+			+		
Gender Equitable Attitudes Scale (Hill et al., 2022; Yonas et al., 2013)	(English)	(1) Emotional and Sexual Stereotypes in Relationships, (2) Moral Code, (3) Heteronormativity									+						
The Jamaican Maternal Sexual Role Modelling (Jamaican MSRM) Questionnaire (Hutchinson et al., 2007, 2012a, 2012b)	(English)	(1) Direct Sexual Modelling, (2) Indirect Sexual Modelling, (3) Sexual Exposure of Daughter														+	+
The Cognitive Susceptibility Index (L'Engle et al., 2006)	(English)	*Initiation of sexual intercourse in younger (especially abstinent) adolescents (unidimensional)*									+			+			
The Youth Sexual Intention Scale (YSIS; Lubis et al., 2022)	Indonesian (English)	(1) Sexual attitude, (2) Subjective norm, (3) Perceived behavior control, (4) Sexual intention						+			+		+	+		+	+
The Masturbation Beliefs Scale (BMS!)–Chinese Version (Ren et al., 2022)	Chinese	(1) Beliefs about male masturbation, (2) Beliefs about female masturbation, (3) Negative affect toward masturbation				+		+			+						
The Makeup Questionnaire (MUQ), The Sexualized Clothing Questionnaire (SCQ; Smith et al., 2017)	(English)	MUQ: (1) Unconfident, (2) Unease; SCQ: (3) Body Discomfort, (4) Pressure									+			+			+
The Attitudes Toward Transactional Sex Scale (St Lawrence et al., 2023)	(English, Setswana)	(1) Perceived Benefits, (2) Refusal Ability, (3) Family Influence, (4) Personal Morals									+		+	+			+
The Contraceptive Behavior Scale (CBS; Wang et al., 2013)	Chinese	*Contraceptive Practice (unidimensional)*		+									+				
The Social Dating Goals Scale-Revised (SDGS-R; Zimmer-Gembeck et al., 2012)	(English)	(1) Identity dating goals, (2) Intimacy dating goals, (3) Status dating goals												+			
HIV and Other STIs Knowledge Scale (KSI; Abello-Luque et al., 2021)	Colombian Spanish, Spanish	(1) HIV transmition knowledge, (2) Other STI knowledge, (3) Condom knowledge, (4) HIV prevention knowledge, (5) General knowledge					+										
Adolescent Students’ Attitudes Scale towards Sexuality (Escala de Atitudes dos Alunos Adolescentes em face da Sexualidade, E3AS; Barros et al., 2020)	Portuguese	(1) Family planning and sex education, (2) First sexual relationship, (3) Violation of sexual rights and who to turn to in the event of an unplanned pregnancy, (4) Gender expression and identity, (5) Unplanned pregnancy and parenting							+		+					+	+
The Sexting Motivations Questionnaire (SMQ; Bianchi et al., 2016)	Italian	(1) Sexual purposes, (2) Instrumental/aggravated reasons, (3) Body image reinforcement												+			+
Adolescent Sexual Expectancies Scale (ASEXS; Bourdeau et al., 2011)	(English)	(1) Social Risk, (2) Social Benefit, (3) Health Risk, (4) Pleasure, (5) Frequency sexual behaviors								+	+		+	+			
Untitled (Buhi et al., 2011)	(English)	*Adolescent Sexual Abstinence* (1) Intentions, (2) Beliefs, (3) Norms, (4) Standards, (5) Positive Emotions, (6) Negative Emotions, (7) Rules, (8) Support									+			+			+
Scale for the Assessment of Sexual Standards among Youth (SASSY; Emmerink et al., 2017)	Dutch	*(Hetero)sexual double standard endorsement (unidimensional)*									+ +						
Condom Use Barriers Scale for Adolescents (CUBS-A; Escribano et al., 2017)	(Spanish)	(1) Negotiation skills, (2) Perceived feelings, (3) Negative aspects of condoms, (4) Disruption of the sexual experience					+			+			+			+	
HIV Attitudes Scale (HIV-AS; Espada et al., 2013; Gómez-Lugo et al., 2020; Morales et al., 2019)	Spanish, Portuguese, Colombian Spanish	(1) Attitudes toward safe sex when there are obstacles, (2) Attitudes toward HIV testing, (3) Attitudes toward condom use, (4) Attitudes toward people living with HIV/AIDS								+		+	+				
Adolescent Clinical Sexual Behavior Inventory-Self-Report (ACSBI-S; Friedrich et al., 2004; Wherry et al., 2009)	(English)	(1) Sexual Knowledge/Interest, (2) Sexual Risk/Misuse, (3) Divergent Sexual Interest, (4) Concerns About Appearance, (5) Fear			+								+	+			
Attitudes Toward Affective-Sexual Diversity Scale (ADAS; Garrido-Hernansaiz et al., 2018)	Spanish	*Attitudes toward homo- and bisexual people (unidimensional)*						+			+			+			
Maternal Health Literacy Scale (MaHeLi Scale; Guttersrud et al., 2015)	English	(1) Health seeking behaviour, (2) Appraisal of health information, (3) Competence and coping skills	+			+				+		+			+		
The Inventory of Anal Sex Knowledge (iASK; Kutner et al., 2022)	(English)	*Douches, fiber, lubrication, anatomy, sexual response, including pleasure, and pain (unidimensional)*				+ +		+									
The Relationship Options Survey (ROS; Lusczakoski & Rue, 2012)	(English)	(1) Sexual activity, (2) Sexual history, (3) Satisfaction primary, (4) Satisfaction tertiary, (5) Secondary contemplation, (6) Secondary Action									( +)		( +)	( +)			
Reproductive Health Literacy Questionnaire (Ma et al., 2021)	Chinese	(1) Accessing, (2) Understanding, (3) Appraising, (4) Applying x (1) Health Promotion, (2) Disease Prevention, (3) Health Care	( +)	( +)	( +)	( +)	( +)	( +)	( +)	( +)	( +)	( +)	( +)	( +)			
The Attitudes Toward Condom Use Scale (ATCUS; Masa & Chowa, 2014)	(English)	(1) Perceived Benefits, (2) Perceived Barriers, (3) Perceived Severity, (4) Perceived Susceptibility, (5) Perceived self-efficacy, (6) Perceived social support					+			+			+			+	
Untitled (Aarø et al., 2011; Mũkoma et al., 2009)	English, Kiswahili, Xhosa, Afrikaans, Sepedi	(1) Knowledge of HIV/AIDS, (2) Attitude towards delay of sexual intercourse, (3) Attitude towards condoms, (4) Attitude towards STDs/ pregnancy, (5) Subjective norms towards delay of sexual intercourse, (6) Subjective norms towards condom use, (7) Injunctive norm, (8) Self efficacy towards delay of sexual intercourse, (9) Self efficacy towards condom use, (10) Perceived condom availability					( +)		( +)	( +)	( +)		( +)	( +)		( +)	
Untitled (Mushwana et al., 2015)	N/A	(1) Attitude of adolescent’s engagement in sexual activities, (2) Psycho-sociological effect of environment, (3) Effect of health service accessibility/availability, (4) Effect of adolescent health/ nursing problems, (5) Relationship with nurses, Approachability	( +)					( +)			( +)				( +)	( +)	( +)
Scale of Knowledge about Sexually Transmitted Infections (Nelas et al., 2014)	Portuguese	*Insufficient, moderate and good knowledge about sexually transmitted infections (unidimensional)*					+			+			+				
Measurement of Bystander Intervention in Bullying and Sexual Harassment (Nickerson et al., 2014)	(English)	(1) Notice the event, (2) Interpret event as an emergency, (3) Accept responsibility to help, (4) Know how to help, (5) Implement intervention decision									+			+			
The Condom Use Negotiated Experiences Through Technology (CuNET) Scale (Okumu et al., 2022)	English, Swahili	*Text-based condom negotiation experiences while sexting (unidimensional)*											+ +				
Sexual Motivations Scale–Revised (SMS-R) & Motivations Against Sex Questionnaire (MASQ; Patrick et al., 2011)	(English)	*Types of Motivation* (1) Enhancement, (2) Intimacy, (3) Coping, (4) Values, (5) Health, (6) Not Ready								+	+		+	+			
The Sexual Relationship Power Scale (SRPS)–Subscale (Pulerwitz et al., 2018)	English, Kiswahili, Luo	*Experiences of violence, HIV risk, levels of sexual relationship power (subscale)*												+		+	+
Untitled (Roye et al., 2007)	English	*Types of intercourse (vaginal, oral, and anal), types of male partners (main, casual, and new), the number of (un-)protected sexual acts (limited to behaviors in the past 2 months and at last intercourse); self-efficacy for condom, nutrition questions*											( +)	( +)			
The Comfort with Sexual Matters for Young Adolescents scale (CWSMYA; Rye et al., 2012)	(English)	*Assess erotophobia-erotophilia with young adolescents (unidimensional)*									+						
Reproductive Health Scale (RHS; Saydam et al., 2010)	Turkish	(1) Partner Selection, (2) Values in Developing Protective Behavior, (3) Communication with Sexual Partner, (4) Consultation, (5) Confidence, (6) Protection from Sexually Transmitted Diseases	( +)	( +)	( +)					( +)	( +)						
Attitudes Toward Sex Education (ATSES; Sim-Sim & Viana, 2017)	Portuguese	(1) Trust Attitudes, (2) Willingness Attitudes		+	+					+	+						
Untitled (Stevens-Simon et al., 2005)	(English)	*Childbearing expectations* (1) Future plans, (2) Self-esteem, (3) Autonomy, (4) Boyfriend relations, (5) Peer relations, (6) Family relations, (7) Be-like relations, (8) Like babies, (9) Need babies							( +)		( +)						
The Sexual Attitudes and Experiences Scale (SAES; Tobin, 2011)	(English)	*Education, Sexual practices* (1) Attitudes, (2) Experiences			+				+	+	+	+	+	+			+
The HIV Knowledge Questionnaire for Adolescent Girls (HIV-KQ AG; Volpe et al., 2007)	(English)	*HIV Knowledge developmentally- and gender-specific for adolescent girls (unidimensional)*					+ +										
Questionnaire on Sexual and Reproductive Health Literacy (Questionnaire on SRHL; Vongxay et al., 2019)	Lao	(1) Access SRH info, (2) Understand SRH info, (3) Appraise SRH info, (4) Apply SRH info	( +)	( +)	( +)	( +)	( +)	( +)	( +)	( +)			( +)				
The Perceived Heterosexism Scale (PHS) & The Preoccupation with Disclosure of Parents’ Sexual Orientation Scale (PDPSOS; Vyncke et al., 2011)	English, French	*Experiences of children raised by gay or lesbian parents* (1) Perceived Heterosexism, (2) Preoccupation with Disclosure of Parent's Sexual Orientation						+			+			+			+
Alcohol and Sexual Consent Scale (Ward et al., 2012)	(English)	(1) Campus Beliefs and Myths, (2) Sexual Assault Programming Messages					+	+		+	+						
Untitled (Yau et al., 2020)	Thai	*Risk and Preventive Behaviors toward Premarital Sexual Practice* (1) Knowledge, (2) Attitude, (3) Perceived Susceptibility, (4) Perceived Severity, (5) Perceived Self-Efficacy				+	+			+	+		+	+			
Stage of Change (SOC) Measure (Yusufov & Orchowski, 2020)	(English)	(1) Readiness to use assertive responding to threat: (1) Precontemplation (PC), (2) Contemplation (3), (3) Action (1), (4) Maintenance (M); (2) Readiness to use self-protective behaviors: (1) PC, (2) C, (3) A, (4) M; (3) Readiness to use open sexual communication: (1) PC, (2) C, (3) A, (4) M											+	+			
Untitled (Zakaria et al., 2020)	Bengali	*Knowledge on, Attitude towards, and Practice of SRH* (1) Knowledge, (2) Attitude, (3) Practice				+	+				+	+		+			+

OMI = Outcome Measurement Instrument, HC = Healthcare, DP = Disease Prevention, HP = Health Promotion, P = Preliminary Study, T = Translation/Adaption Study, V = Validation Study; OMIs are sorted by quality of *Total Development* followed by the quality of *Total OMI-Design*; (Language) = The OMI language was not explicitly mentioned in the publication itself and was derived from the context of the study; SH = Sexual Health, SRH = Sexual and Reproductive Health, PC = Precontemplation, C = Contemplation, A = Action, M = Maintenance, STI = Sexually Transmitted Infections, HIV = Human Immunodeficiency Virus; +  = dimension is considered in the OMI, +  +  = dimension is covered comprehensively due to the relevant health area, (+ / + +) = original items were not available and the information was derived from the description of the OMI in the study

On average, OMIs covered 2 of the 5 dimensions (*Mdn* = 2, *IQR* = 1–4). The OMIs more frequently captured appraisal (*n* = 47, 69%) and application (*n* = 45, 66%) of sexual health related information, followed by context factors (*n* = 26, 38%), understanding (*n* = 24, 35%) and accessing information (*n* = 20, 29%). Two OMIs addressed all 5 areas of SHL (Guttersrud et al., 2015; Jerman et al., 2015).

In differentiating between healthcare, prevention, and health promotion dimensions, most OMIs addressed two dimensions (*Mdn* = 2, *IQR* = 1–2). Healthcare issues were considered in 24 (35%), prevention in 45 (66%) and health promotion issues in 55 (81%) OMIs. Overall, 15 (22%) OMIs addressed all three dimensions.

Regarding conceptual fit, 6 (9%) OMIs instruments received high ratings (++), albeit in different dimensions. No OMI was comprehensive in more than one dimension of SHL.

#### Quality of the Outcome Measurement Instrument Development

In the quality assessment (see Table 3), 51 (75%) OMIs were rated as inadequate, and 17 (25%) as doubtful. No OMI received a higher rating than doubtful.

**Table 3:** Box 1 ratings

References	OMI	OMI design							Cognitive interview (CI) study^b^				TOTAL OMI DEVELOPMENT
		General design requirements^c^					Concept elicitation^a^	Total OMI design	General design requirements^c^	Comprehensibility	Comprehensiveness	Total CI study	
		Clear construct	Clear origin of construct	Clear target population	Clear context of use	OMI developed in sample representing the target population							
Acharya et al. (2016)	Sexual Health Questionnaire	V	V	V	V	A	D	D	A	D	D	D	D
Berglas et al. (2017)	Measures of Adolescents’ Attitudes about Sexual Relationship Rights	V	V	V	V	V	D	D	V	D	D	D	D
Chu et al. (2005)	The Adolescent Masculinity Ideology in Relationships Scale (AMIRS)	V	V	V	V	V	D	D	V	D	D	D	D
Darabi et al. (2018)	Belief-Based Reproductive Health Questionnaire (BBRHQ)	V	V	V	V	V	D	D	V	D	D	D	D
Deardorff et al. (2008)	Untitled	V	V	V	V	V	D	D	A	D	D	D	D
Ghiasi et al. (2022)	The Married Adolescent Women's Sexual and Reproductive Health Needs Assessment Questionnaire (MAWSRHNAQ)	V	D	V	V	V	D	D	V	D	D	D	D
Guerra et al. (2019)	Scale of Myths about Sexuality (Escala de Mitos sobre la Sexualidad, EMS)	V	V	V	V	V	D	D	V	D	D	D	D
Jerman et al. (2015)	Untitled	V	V	V	V	V	D	D	V	D	D	D	D
O'Sullivan et al. (2006)	The Sexual Self-Concept Inventory (SSCI)	V	V	V	V	V	D	D	A	D	D	D	D
Prati (2012)	The Homophobic Bullying Scale	V	V	V	V	A	D	D	V	D	D	D	D
Reininger et al. (2003)	Adolescent Health Attitude and Behavior Survey (AHABS)	V	V	V	V	D	D	D	V	D	D	D	D
Sales et al. (2008)	The Parent-Adolescent Communication Scale (PACS)	V	V	V	V	V	D	D	V	D	D	D	D
Sales et al. (2009)	The Worry About Sexual Outcomes (WASO) Scale	V	V	V	V	V	D	D	V	D	D	D	D
Sánchez et al. (2015)	Cyberdating Q_A	V	V	V	V	V	D	D	V	D	D	D	D
Stoebenau et al. (2023), P = Kyegombe et al. (2020)	The Gender Roles and Male Provision Expectations (GRMPE) scale	V	V	V	V	V	D	D	V	A	D	D	D
Ullman et al. (2023), P = Ullman (2014)	The Gender Climate Scale (GCS)	V	V	V	V	V	D	D	V	D	D	D	D
Upadhyay et al. (2021), P = Fefferman and Upadhyay (2018), P = Upadhyay and Lipkovich (2020)	The Sexual and Reproductive Empowerment Scale for Adolescents and Young Adults	V	V	V	V	A	D	D	V	D	D	D	D
Edwards et al. (2018), P = (Edwards et al., 2015)	Untitled	V	V	V	V	V	V	V	NA	NA	NA	I	I
Grover et al. (2005), P = Grover et al. (2007)	The Measure of Adolescent Heterosocial Competence (MAHC)	V	V	V	V	V	V	V	V	D	D	D	I
Apidechkul (2019)	Untitled	V	V	V	V	V	D	D	V	D	D	D	I
Biney (2016)	Sexual Self-Concept (SSC) Scale	V	V	V	V	V	D	D	NA	NA	NA	I	I
Fisher et al. (2018)	Untitled	V	V	V	V	V	D	D	V	D	D	D	I
Hannawa et al. (2006)	Relational Entitlement and Proprietariness Scale (REPS)	V	V	V	V	V	D	D	NA	NA	NA	I	I
Hendriks et al. (2012), P = Skinner et al. (2008), P = Skinner et al. (2009)	The Adolescent Attitudes to Abortion Scale (AAA Scale)	V	V	V	V	D	D	D	NA	NA	NA	I	I
Hill et al. (2022), P = Yonas et al. (2013)	Gender Equitable Attitudes Scale	V	V	V	V	A	D	D	A	I	D	I	I
Hutchinson et al., (2012a, 2012b), P = Hutchinson et al. (2007), P = Hutchinson et al., (2012a, 2012b)	The Jamaican Maternal Sexual Role Modelling (Jamaican MSRM) Questionnaire	V	V	V	V	V	D	D	A	I	D	I	I
L'Engle et al. (2006)	The Cognitive Susceptibility Index	V	V	V	V	V	D	D	NA	NA	NA	I	I
Lubis et al. (2022)	The Youth Sexual Intention Scale (YSIS)	V	V	V	V	V	D	D	NA	NA	NA	I	I
Ren et al. (2022)	The Masturbation Beliefs Scale (BMS!)–Chinese Version	V	V	V	V	A	D	D	I	I	D	I	I
Smith et al. (2017)	The Makeup Questionnaire (MUQ), The Sexualized Clothing Questionnaire (SCQ)	V	V	V	V	V	D	D	NA	NA	NA	I	I
St Lawrence et al. (2023)	The Attitudes Toward Transactional Sex Scale	V	V	V	V	V	D	D	NA	NA	NA	I	I
Wang et al. (2013)	The Contraceptive Behavior Scale (CBS)	V	V	V	V	D	D	D	A	I	D	I	I
Zimmer-Gembeck et al. (2012)	The Social Dating Goals Scale-Revised (SDGS-R)	V	V	V	V	D	D	D	NA	NA	NA	I	I
Abello-Luque et al. (2021)	HIV and Other STIs Knowledge Scale (KSI)	V	V	V	V	I	NA	I	NA	NA	NA	I	I
Barros et al. (2020)	Adolescent Students’ Attitudes Scale towards Sexuality (Escala de Atitudes dos Alunos Adolescentes em face da Sexualidade, E3AS)	V	V	V	V	I^d^	NA	I	V	D	D	D	I
Bianchi et al. (2016)	The Sexting Motivations Questionnaire (SMQ)	V	V	V	D	I^d^	NA	I	NA	NA	NA	I	I
Bourdeau et al. (2011)	Adolescent Sexual Expectancies Scale (ASEXS)	V	V	V	V	I	NA	I	NA	NA	NA	I	I
Buhi et al. (2011)	Untitled	V	V	V	V	I	NA	I	V	I	D	I	I
Emmerink et al. (2017)	Scale for the Assessment of Sexual Standards among Youth (SASSY)	V	V	V	V	I	NA	I	NA	NA	NA	I	I
Escribano et al. (2017)	Condom Use Barriers Scale for Adolescents (CUBS-A)	V	V	V	V	I^d^	NA	I	A	D	D	D	I
Espada et al. (2013), T = Morales et al. (2019), T = Gómez-Lugo et al. (2020)	HIV Attitudes Scale (HIV-AS)	V	V	V	V	I^d^	NA	I	V	D	D	D	I
Friedrich et al. (2004)	Adolescent Clinical Sexual Behavior Inventory-Self-Report (ACSBI-S)	V	V	V	V	I	NA	I	V	D	D	D	I
Garrido-Hernansaiz et al. (2018)	Attitudes Toward Affective-Sexual Diversity Scale (ADAS)	V	V	V	V	I^d^	NA	I	NA	NA	NA	I	I
Guttersrud et al. (2015)	Maternal Health Literacy Scale (MaHeLi Scale)	V	V	V	V	I	NA	I	NA	NA	NA	I	I
Kutner et al. (2022)	The Inventory of Anal Sex Knowledge (iASK)	V	D	V	V	I^d^	NA	I	V	I	D	I	I
Lusczakoski and Rue (2012)	The Relationship Options Survey (ROS)	V	V	V	V	I^d^	NA	I	NA	NA	NA	I	I
Ma et al. (2021)	Reproductive Health Literacy Questionnaire	V	V	V	V	I^d^	NA	I	V	I	D	I	I
Masa and Chowa (2014)	The Attitudes Toward Condom Use Scale (ATCUS)	V	V	V	V	I^d^	NA	I	V	D	D	D	I
Mũkoma et al. (2009)	Untitled	V	V	V	V	I^d^	NA	I	V	I	D	I	I
Mushwana et al. (2015)	Untitled	V	V	V	V	I	NA	I	NA	NA	NA	I	I
Nelas et al. (2014)	Scale of Knowledge about Sexually Transmitted Infections	V	V	V	V	I	NA	I	NA	NA	NA	I	I
Nickerson et al. (2014)	Measurement of Bystander Intervention in Bullying and Sexual Harassment	V	V	V	V	I	NA	I	NA	NA	NA	I	I
Okumu et al. (2022)	The Condom Use Negotiated Experiences Through Technology (CuNET) Scale	V	V	V	V	I^d^	NA	I	V	I	D	I	I
Patrick et al. (2011)	Sexual Motivations Scale–Revised (SMS-R) & Motivations Against Sex Questionnaire (MASQ)	V	V	V	V	I	NA	I	NA	NA	NA	I	I
Pulerwitz et al. (2018)	The Sexual Relationship Power Scale (SRPS)—Subscale	V	V	V	V	I	NA	I	NA	NA	NA	I	I
Roye et al. (2007)	Untitled	V	V	V	V	I	NA	I	V	D	D	D	I
Rye et al. (2012)	The Comfort with Sexual Matters for Young Adolescents scale (CWSMYA)	V	V	V	V	I^d^	NA	I	NA	NA	NA	I	I
Saydam et al. (2010)	Reproductive Health Scale (RHS)	V	V	V	V	I	NA	I	V	D	D	D	I
Sim-Sim and Viana (2017)	Attitudes Toward Sex Education (ATSES)	V	V	V	V	I	NA	I	V	D	D	D	I
Stevens-Simon et al. (2005)	Untitled	V	V	V	V	I	NA	I	V	I	D	I	I
Tobin (2011)	The Sexual Attitudes and Experiences Scale (SAES)	V	V	V	V	I	NA	I	V	D	D	D	I
Volpe et al. (2007)	The HIV Knowledge Questionnaire for Adolescent Girls (HIV-KQ AG)	V	V	V	V	I	NA	I	NA	NA	NA	I	I
Vongxay et al. (2019)	Questionnaire on Sexual and Reproductive Health Literacy (Questionnaire on SRHL)	V	V	V	V	I	NA	I	V	I	D	I	I
Vyncke et al. (2011)	The Perceived Heterosexism Scale (PHS) & The Preoccupation with Disclosure of Parents’ Sexual Orientation Scale (PDPSOS)	V	V	V	V	I^d^	NA	I	NA	NA	NA	I	I
Ward et al. (2012)	Alcohol and Sexual Consent Scale	V	V	V	V	I	NA	I	A	I	D	I	I
Yau et al. (2020)	Untitled	V	V	V	V	I^d^	NA	I	V	I	D	I	I
Yusufov and Orchowski (2020)	Stage of Change (SOC) Measure	V	V	V	V	I	NA	I	D	I	D	I	I
Zakaria et al. (2020)	Untitled	V	V	V	V	I	NA	I	V	D	D	I	I

OMI = Outcome Measurement Instrument, P = Preliminary Study, T = Translation/Adaption Study, V = very good, A = adequate, D = doubtful, I = inadequate, NA = not applicable; OMIs are sorted by quality of *Total Development* followed by the quality of *Total OMI-Design*

^a^When the PROM was not developed in a sample representing the target population, the concept elicitation was not further rated

^b^NA indicates that a CI study (or part of it) was not performed

^c^CI study performed in sample representing the target population

^d^Experts were consulted while designing the OMI

Related to its design process, 35 (51%) OMIs were rated overall as inadequate, 31 (46%) OMIs as doubtful, no OMI as adequate, and 2 (3%) as very good. In all cases, inadequate ratings were due to the lack of involvement of the target population (for further details on the design processes of studies not directly involving the target population, see the Discussion section under Quality of the OMI Development). Very good rated studies developed the Measure of Adolescent Heterosocial Competence (Grover et al., 2005, 2007), and an untitled scale measuring relationship abuse and sexual assault (Edwards et al., 2015, 2018) reporting the involvement of the target population in the concept elicitation process regarding relevance and comprehensiveness.

The cognitive interview study, in which the OMI has to be piloted within the target population, was rated inadequate for 39 (57%) OMIs, doubtful for 29 (43%) OMIs, and adequate or very good for none of the OMIs. In many of the inadequate cases, this was because no pilot study was reported at all.

As none of the OMIs were overall rated better than doubtful in the development phase, the boxes 2–10 were not assessed for any of the OMIs. It was not considered reasonable to proceed with reporting psychometric properties as it could be misleading (Best et al., 2023). This should only occur after addressing the deficiencies identified during the development phase (see Table 3).

## Discussion

### Included Studies

This systematic review examined self-report OMIs of SHL, specifically developed for adolescent samples. The review followed COSMIN recommendations (Mokkink et al., 2016; Prinsen et al., 2016; Terwee et al., 2018a, 2018b). The analysis covered 68 OMIs, evaluating both their content fit to SHL dimensions and their developmental quality.

### Topics of the Outcome Measurement Instruments and Conceptual Fit with Sexual Health Literacy

Identified OMIs covered different aspects that need to be considered in SHL research, ranging from the assessment or recording of knowledge and behavior to more competence-oriented measurements (Palmer et al., 2019). The appraisal and application of sexual health information was measured frequently, followed by questions about understanding SH information (Sørensen et al., 2013). Contextual factors and the access to information on SH were less frequently measured. Since setting and the conditions in which adolescents grow up can influence how they ultimately process and use health-related information, these factors require further emphasis in research (Phongluxa et al., 2020).

While disease prevention and health care issues are important issues in sexuality education for decades, sexual health promotion has become increasingly important, as reflected by the WHO definition of sexual health (WHO, 2006). Accordingly, 81% of OMIs (*n* = 55) included recreational aspects of SH and well-being alongside functional limitations or health risks.

Many of the included OMIs are highly specific to a particular topic—e.g., the Gender Climate Scale (Ullman et al., 2023), or the Masturbation Beliefs Scale (Ren et al., 2022)–and are therefore useful for capturing particular nuances of certain sexuality domains. Despite their strengths, they cannot provide a comprehensive picture of sexual health literacy (SHL) across various contexts. An additional generic OMI that highlights SHL could be valuable. This would complement the existing specific measures and allow us to analyze SHL as an umbrella term, exploring its links to sexual health, as it is already done to some extent (e.g., Goodwin, 2021; Lirios et al., 2024; Panahi et al., 2022; Väisänen et al., 2021; Vamos et al., 2020). It would improve comparability of assessments and interventions regarding their impact on the SH of adolescents. It would minimize the need to develop separate measurement tools used only once for each of these topics, different cultures and interventions as is often the case today (Rogers et al., 2022; Tavousi et al., 2022; Yafi et al., 2020). Even if new topics emerge in our understanding of sexual health (e.g., virtual sexuality), interventions could be examined on how they are beneficial to adolescents’ SHL. Generic OMIs, that are not topic-specific or only relevant for certain target groups are suitable for further inspection (e.g., Acharya et al., 2016; Barros et al., 2020; Darabi et al., 2018; Rye et al., 2012). Those of them which items covered different aspects of SHL, i.e. at least two SHL dimensions, and are available in English (Bourdeau et al., 2011; Deardorff et al., 2008; Jerman et al., 2015; Lubis et al., 2022; Reininger et al., 2003; Tobin, 2011; Upadhyay et al., 2021) may be a promising start.

### Quality of the Outcome Measurement Instrument Development

A large number of OMIs received an inadequate overall rating, as the studies lacked relevant information, e.g., sample details or analysis information (compare Best et al., 2023). Many studies primarily focused on validating the OMIs, often at the expense of thoroughly reporting the development process. Insufficient ratings should not necessarily be attributed to poor research quality (e.g., not involving the target group in developing an OMI), but in some cases, they might also be due to the insufficient documentation of the development steps and lack of transparency in the research process (Guo et al., 2018).

Moreover, the insufficient quality ratings were often because the OMIs were not designed within the target group, but only through consultation of experts (Barros et al., 2020; Bianchi et al., 2016; Escribano et al., 2017; Espada et al., 2013; Garrido-Hernansaiz et al., 2018; Kutner et al., 2022; Lusczakoski & Rue, 2012; Ma et al., 2021; Masa & Chowa, 2014; Mũkoma et al., 2009; Okumu et al., 2022; Rye et al., 2012; Vyncke et al., 2011; Yau et al., 2020). This issue has been highlighted before (Lénárt et al., 2020). In some cases, elaborate procedures were carried out, but experts may not be able to fully empathize with the young people. The involvement of adolescents is particularly important, as age-appropriate and easily understandable language is required at this stage (Cotton et al., 2010; Henje et al., 2020; Weinhardt et al., 1998). Furthermore, items designed for an older population might not be relevant to adolescents due to a lack of practical experiences (Sopfe et al., 2021). It is important that researchers do not blindly rely on existing OMIs for adults and apply them to younger cohorts, but rather choose OMIs carefully and check their suitability for adolescents. This process should involve balancing the perspectives of both the target group and experts in the field, to ensure the measures are relevant and appropriate for the specific needs of adolescents while maintaining scientific rigor.

Another part of developing an OMI is piloting it within the target population. Pilot studies were often not conducted in a qualitative manner (Abello-Luque et al., 2021; Bianchi et al., 2016; Biney, 2016; Bourdeau et al., 2011; Edwards et al., 2018; Emmerink et al., 2017; Garrido-Hernansaiz et al., 2018; Guttersrud et al., 2015; Hannawa et al., 2006; Hendriks et al., 2012; L'Engle et al., 2006; Lubis et al., 2022; Lusczakoski & Rue, 2012; Mushwana et al., 2015; Nelas et al., 2014; Nickerson et al., 2014; Patrick et al., 2011; Pulerwitz et al., 2018; Rye et al., 2012; Smith et al., 2017; St Lawrence et al., 2023; Volpe et al., 2007; Vyncke et al., 2011; Zimmer-Gembeck et al., 2012). Studies rated as doubtful showed a wide range of methodological quality. For example some studies briefly stated that the OMI was piloted (Hill et al., 2022), while some were more elaborate yet their pilot report was too inaccurate for achieving a better rating (Stoebenau et al., 2023). In some cases it was not clear whether the pilot study assessed the comprehensibility of the items leading to an inadequate overall rating (Apidechkul, 2019; Fisher et al., 2018; Grover et al., 2005). In addition to comprehensibility, comprehensiveness must also be considered to ensure that the instrument captures all relevant aspects of sexual health literacy. The latter–comprehensiveness–was hardly ever explicitly considered in the pilot studies. Therefore, it is not known for any OMI whether the respective scale comprehensively captures what the relevant aspects include in the sense of adolescents (Sopfe et al., 2021).

The decision not to report any additional stated psychometric properties was made since none of the OMIs had received high ratings for their development. Therefore, OMIs may still be well-suited for the target group, for instance, by involving experts from the field, but this step was missing in many reports. For a thorough understanding of the psychometric properties of instruments, though, it is essential that the target group comprehends them as intended (Weinhardt et al., 1998). Active participation of them is hence vital to ensure accurate measurement of the outcomes (Lénárt et al., 2020).

### Limitations

#### Search

As the comprehensive definition of SHL is quite novel, the present review included many OMIs that appeared to measure some aspects of SHL. As the focus of the present review was on self-report measures and not on proxy reports—assessments completed by other individuals, such as parents—some scales were not included (Vongxay et al., 2022). Therefore, investigating OMIs as proxy ratings is an important area of future research. Moreover, some Spanish- and Portuguese-language articles may be of interest and could be reviewed in addition (see Supplement 7). In the present review they were excluded due to limited language capabilities.

#### Content

The content analysis was merely narrative. The study therefore only provides an initial assessment of the consideration of SHL components in the current research landscape. The OMIs were assessed at item level where possible. The decision whether a dimension was considered comprehensive due to the regarding topic was based solely on the raters’ assessment and not validated by the target population.

#### COSMIN

The focus of the current study was on the development quality, which repeatedly revealed poor ratings. It should be noted that COSMIN prescribes that the assessment of psychometric properties should be omitted only after the assessment of content validity and an insufficient overall result (Prinsen et al., 2018). For a valid assessment of content validity, information on relevance, comprehensiveness and/or comprehensibility should be considered from different perspectives (adolescents and experts) in addition to the developmental study (Prinsen et al., 2018). This information was only available in few cases in the present studies (Acharya et al., 2016; Darabi et al., 2018; Ghiasi et al., 2022; Hutchinson et al., 2012a, 2012b; Lubis et al., 2022; Mũkoma et al., 2009; Ren et al., 2022; Stoebenau et al., 2023). Furthermore, low ratings of development quality point to shortcomings in current research. Low quality ratings are achieved, for instance, when information is not presented explicitly and accurately. They provide limited insights into the actual quality of the underlying processes, focusing more on the documentation quality of those processes. As there are usually only single studies on OMI development and their validation in the field of sexuality education, it is even more essential that all available information should be provided to create as much transparency in research as possible.

### Implications

This review shows the status quo of self-report outcome measurement instruments used among adolescents and compares the extent to which they consider–often implicitly–aspects of SHL. In terms of content, no instrument could be identified in the present study that would adequately cover SHL (Martin, 2017). Nevertheless, it was shown that a wide range of aspects have already been considered in research over the last two decades (e.g., knowledge, attitudes, interpersonal action). Despite this, OMIs often focused on individual areas of sexual health, and more generic perspectives on SHL were missing. An additional generic SHL approach could complement specific measures and inform research and practice in the long term due to its independence from whether our understanding of single issues considered *healthy* today will change.

The ratings for the development process of the survey instruments were poor. Even if the COSMIN rating turns out to be a very rigorous rating system, the present study shows that there is a significant need for improvement. In future research, greater emphasis should be placed on involving the target group. In addition, the documentation of pilot studies (e.g., regarding the exact sample characteristics) was frequently insufficient. Authors and peer reviewers should use checklists, before publishing, to be able to review the study quality based on clear predefined criteria. The COSMIN guidelines, for example, could assist in the development phase of an OMI. Furthermore, few studies focused solely on the development of the questionnaires. Developing a high-quality OMI together with the target population is very time-consuming. If studies that only develop the OMIs and initially ignore other psychometric properties would be published independently, this may improve quality.

This review provides practitioners with an initial overview to help them select the most relevant instruments based on their specific needs. It emphasizes the importance of clearly defining what one intends to measure before selecting an instrument. Questionnaires such as the Gender Climate Scale (Ullman et al., 2023) are presented as tools for addressing specific research questions in particular contexts. If the aim is to assess a specific domain of sexual health literacy (SHL), such as the ability to update oneself on determinants of sexual health in the social and physical environment (Access*Health promotion), this review may also provide a foundation for selecting relevant instruments, such as those by Jerman et al. (2015) or Reininger et al. (2003). The findings also highlight that none of the available instruments comprehensively address all aspects of SHL—namely Access, Understand, Appraise, and Apply. Until a more generic instrument spanning all these dimensions is developed, practitioners may consider combining general health literacy scales–not explicitly related to sexual health–such as the HLS-EU-Q (Sørensen et al., 2013), with specific sexual health measurement tools to complement the measurement of specific aspects of sexual health with broader health information.

## Supplementary Information

Below is the link to the electronic supplementary material.

Supplementary file1 (PDF 163 KB)

Supplementary file2 (PDF 71 KB)

Supplementary file3 (PDF 100 KB)

Supplementary file4 (PDF 1029 KB)

Supplementary file5 (PDF 188 KB)

Supplementary file6 (PDF 124 KB)

Supplementary file7 (PDF 84 KB)
