# Supplementary material for: A COSMIN Systematic Review of Sexual Health Literacy Self-Report Measures for Adolescents
Source: Arch Sex Behav. 2025 Jun 6;54(5):1737–68. doi: 10.1007/s10508-025-03142-1 (PMC12162768; doi:10.1007/s10508-025-03142-1)
Supplement: Supplementary file 1 — Supplementary file1 (PDF 163 KB) [file 10508_2025_3142_MOESM1_ESM.pdf]

Search Strategies

Filters applied: time span (2002-2022)

Pubmed

((sexual*[tiab] *OR* reproductive[tiab]) *AND* (Literacy[tiab] *OR* literate[tiab] *OR* numeracy[tiab] *OR* knowledge[tiab] *OR* belief*[tiab] *OR* attitud*[tiab] *OR* motivation*[tiab] *OR* competenc*[tiab] *OR* skills*[tiab])) *AND* (adolescen*[tiab] *OR* teen*[tiab] *OR* youth[tiab] *OR* minor*[tiab] *OR* young[tiab] *OR* pupil*[tiab] *OR* student*[tiab]) *AND* (((self[tiab] *OR* child[tiab] *OR* parent[tiab] *OR* carer[tiab] *OR* proxy[tiab]) *AND* ((report[tiab] *OR* reported[tiab] *OR* reporting[tiab]) *OR* (rated[tiab] *OR* rating[tiab] *OR* ratings[tiab]) *OR* based[tiab] *OR* (assessed[tiab] *OR* assessment[tiab] *OR* assessments[tiab]))) *OR* (outcome[tiab] *OR* outcomes[tiab] *OR* index[tiab] *OR* indices[tiab] *OR* instrument[tiab] *OR* instruments[tiab] *OR* measure[tiab] *OR* measures[tiab] *OR* questionnaire[tiab] *OR* questionnaires[tiab] *OR* profile[tiab] *OR* profiles[tiab] *OR* scale[tiab] *OR* scales[tiab] *OR* score[tiab] *OR* scores[tiab] *OR* status[tiab] *OR* survey[tiab] *OR* surveys[tiab])) *AND* ((instrumentation[sh] *OR* methods[sh] *OR* "Validation Studies"[pt] *OR* "Comparative Study"[pt] *OR* "psychometrics"[MeSH] *OR* psychometr*[tiab] *OR* clinimetr*[tw] *OR* clinometr*[tw] *OR* "outcome assessment (health care)"[MeSH] *OR* "outcome assessment"[tiab] *OR* "outcome measure*"[tw] *OR* "observer variation"[MeSH] *OR* "observer variation"[tiab] *OR* "Health Status Indicators"[MeSH] *OR* "reproducibility of results"[MeSH] *OR* reproducib*[tiab] *OR* "discriminant analysis"[MeSH] *OR* reliab*[tiab] *OR* unreliab*[tiab] *OR* valid*[tiab] *OR* "coefficient of variation"[tiab] *OR* coefficient[tiab] *OR* homogeneity[tiab] *OR* homogeneous[tiab] *OR* "internal consistency"[tiab] *OR* (cronbach*[tiab] *AND* (alpha[tiab] *OR* alphas[tiab])) *OR* (item[tiab] *AND* (correlation*[tiab] *OR* selection*[tiab] *OR* reduction*[tiab])) *OR* agreement[tw] *OR* precision[tw] *OR* imprecision[tw] *OR* "precise values"[tw] *OR* test-retest[tiab] *OR* (test[tiab] *AND* retest[tiab]) *OR* (reliab*[tiab] *AND* (test[tiab] *OR* retest[tiab])) *OR* stability[tiab] *OR* interrater[tiab] *OR* inter-rater[tiab] *OR* intrarater[tiab] *OR* intra-rater[tiab] *OR* intertester[tiab] *OR* inter-tester[tiab] *OR* intratester[tiab] *OR* intra-tester[tiab] *OR* interobserver[tiab] *OR* inter-observer[tiab] *OR* intraobserver[tiab] *OR* intra-observer[tiab] *OR* intertechnician[tiab] *OR* inter-technician[tiab] *OR* intratechnician[tiab] *OR* intra-technician[tiab] *OR* interexaminer[tiab] *OR* inter-examiner[tiab] *OR* intraexaminer[tiab] *OR* intra-examiner[tiab] *OR* interassay[tiab] *OR* inter-assay[tiab] *OR* intraassay[tiab] *OR* intra-assay[tiab] *OR* interindividual[tiab] *OR* inter-individual[tiab] *OR* intraindividual[tiab] *OR* intra-individual[tiab] *OR* interparticipant[tiab] *OR* inter-participant[tiab]

OR intraparticipant**[tiab]** *OR* intra-participant**[tiab]** *OR* kappa**[tiab]** *OR* kappa's**[tiab]** *OR* kappas**[tiab]** *OR* repeatab*****[tw]** *OR* ((replicab*****[tw]** *OR* repeated**[tw]**) *AND* (measure**[tw]** *OR* measures**[tw]** *OR* findings**[tw]** *OR* result**[tw]** *OR* results**[tw]** *OR* test**[tw]** *OR* tests**[tw]**)) *OR* generaliza*****[tiab]** *OR* generalisa*****[tiab]** *OR* concordance**[tiab]** *OR* (intraclass**[tiab]** *AND* correlation*****[tiab]**) *OR* discriminative**[tiab]** *OR* "known group"**[tiab]** *OR* "factor analysis"**[tiab]** *OR* "factor analyses"**[tiab]** *OR* "factor structure"**[tiab]** *OR* "factor structures"**[tiab]** *OR* dimension*****[tiab]** *OR* subscale*****[tiab]** *OR* (multitrait**[tiab]** *AND* scaling**[tiab]** *AND* (analysis**[tiab]** *OR* analyses**[tiab]**)) *OR* "item discriminant"**[tiab]** *OR* "interscale correlation*"**[tiab]** *OR* error**[tiab]** *OR* errors**[tiab]** *OR* "individual variability"**[tiab]** *OR* "interval variability"**[tiab]** *OR* "rate variability"**[tiab]** *OR* (variability**[tiab]** *AND* (analysis**[tiab]** *OR* values**[tiab]**)) *OR* (uncertainty**[tiab]** *AND* (measurement**[tiab]** *OR* measuring**[tiab]**)) *OR* "standard error of measurement"**[tiab]** *OR* sensitiv*****[tiab]** *OR* responsive*****[tiab]** *OR* (limit**[tiab]** *AND* detection**[tiab]**) *OR* "minimal detectable concentration"**[tiab]** *OR* interpretab*****[tiab]** *OR* ((minimal**[tiab]** *OR* minimally**[tiab]** *OR* clinical**[tiab]** *OR* clinically**[tiab]**) *AND* (important**[tiab]** *OR* significant**[tiab]** *OR* detectable**[tiab]**) *AND* (change**[tiab]** *OR* difference**[tiab]**)) *OR* (small*****[tiab]** *AND* (real**[tiab]** *OR* detectable**[tiab]**) *AND* (change**[tiab]** *OR* difference**[tiab]**)) *OR* "meaningful change"**[tiab]** *OR* "ceiling effect"**[tiab]** *OR* "floor effect"**[tiab]** *OR* "Item response model"**[tiab]** *OR* IRT**[tiab]** *OR* Rasch**[tiab]** *OR* "Differential item functioning"**[tiab]** *OR* DIF**[tiab]** *OR* "computer adaptive testing"**[tiab]** *OR* "item bank"**[tiab]** *OR* "cross-cultural equivalence"**[tiab]**)) *NOT* (("addresses"**[pt]** *OR* "biography"**[pt]** *OR* "case reports"**[pt]** *OR* "comment"**[pt]** *OR* "directory"**[pt]** *OR* "editorial"**[pt]** *OR* "festschrift"**[pt]** *OR* "interview"**[pt]** *OR* "lectures"**[pt]** *OR* "legal cases"**[pt]** *OR* "legislation"**[pt]** *OR* "letter"**[pt]** *OR* "news"**[pt]** *OR* "newspaper article"**[pt]** *OR* "patient education handout"**[pt]** *OR* "popular works"**[pt]** *OR* "congresses"**[pt]** *OR* "consensus development conference"**[pt]** *OR* "consensus development conference, nih"**[pt]** *OR* "practice guideline"**[pt]**) *NOT* ("animals"**[MeSH]** *NOT* "humans"**[MeSH]**))

Embase

#1) (('sexual*':**ti,ab,kw** *OR* 'reproductive':**ti,ab,kw**) *AND* ('literacy':**ti,ab,kw** *OR* 'literate':**ti,ab,kw** *OR* 'numeracy':**ti,ab,kw** *OR* 'knowledge':**ti,ab,kw** *OR* 'belief*':**ti,ab,kw** *OR* 'attitud*':**ti,ab,kw** *OR* 'motivation*':**ti,ab,kw** *OR* 'competenc*':**ti,ab,kw** *OR* 'skills*':**ti,ab,kw**))

#2) ('adolescen*':**ti,ab,kw** *OR* 'teen*':**ti,ab,kw** *OR* 'youth':**ti,ab,kw** *OR* 'minor*':**ti,ab,kw** *OR* 'young':**ti,ab,kw** *OR* 'pupil*':**ti,ab,kw** *OR* 'student*':**ti,ab,kw**)

#3) ((('self':**ti,ab** *OR* 'child':**ti,ab** *OR* 'parent':**ti,ab** *OR* 'carer':**ti,ab** *OR* 'proxy':**ti,ab**) *AND* (('report':**ti,ab** *OR* 'reported':**ti,ab** *OR* 'reporting':**ti,ab**) *OR* ('rated':**ti,ab** *OR* 'rating':**ti,ab** *OR* 'ratings':**ti,ab**) *OR* 'based':**ti,ab** *OR* ('assessed':**ti,ab** *OR* 'assessment':**ti,ab** *OR* 'assessments':**ti,ab**))) *OR* ('outcome':**ti,ab** *OR* 'outcomes':**ti,ab** *OR* 'index':**ti,ab** *OR* 'indices':**ti,ab** *OR* 'instrument':**ti,ab** *OR* 'instruments':**ti,ab** *OR* 'measure':**ti,ab** *OR* 'measures':**ti,ab** *OR* 'questionnaire':**ti,ab** *OR* 'questionnaires':**ti,ab** *OR* 'profile':**ti,ab** *OR* 'profiles':**ti,ab** *OR* 'scale':**ti,ab** *OR* 'scales':**ti,ab** *OR* 'score':**ti,ab** *OR* 'scores':**ti,ab** *OR* 'status':**ti,ab** *OR* 'survey':**ti,ab** *OR* 'surveys':**ti,ab**))

#4) ('intermethod comparison'**/exp** *OR* 'data collection method'**/exp** *OR* 'validation study'**/exp** *OR* 'feasibility study'**/exp** *OR* 'pilot study'**/exp** *OR* 'psychometry'**/exp** *OR* 'reproducibility'**/exp** *OR* reproducib*:**ti,ab** *OR* 'audit':**ti,ab** *OR* psychometr*:**ti,ab** *OR* clinimetr*:**ti,ab** *OR* clinometr*:**ti,ab** *OR* 'observer variation'**/exp** *OR* 'observer variation':**ti,ab** *OR* 'discriminant analysis'**/exp** *OR* 'validity'**/exp** *OR* reliab*:**ti,ab** *OR* valid*:**ti,ab** *OR* 'coefficient':**ti,ab** *OR* 'internal consistency':**ti,ab** *OR* (cronbach*:**ti,ab** *AND* ('alpha':**ti,ab** *OR* 'alphas':**ti,ab**)) *OR* 'item correlation':**ti,ab** *OR* 'item correlations':**ti,ab** *OR* 'item selection':**ti,ab** *OR* 'item selections':**ti,ab** *OR* 'item reduction':**ti,ab** *OR* 'item reductions':**ti,ab** *OR* 'agreement':**ti,ab** *OR* 'precision':**ti,ab** *OR* 'imprecision':**ti,ab** *OR* 'precise values':**ti,ab** *OR* 'test-retest':**ti,ab** *OR* ('test':**ti,ab** *AND* 'retest':**ti,ab**) *OR* (reliab*:**ti,ab** *AND* ('test':**ti,ab** *OR* 'retest':**ti,ab**)) *OR* 'stability':**ti,ab** *OR* 'interrater':**ti,ab** *OR* 'inter-rater':**ti,ab** *OR* 'intrarater':**ti,ab** *OR* 'intra-rater':**ti,ab** *OR* 'intertester':**ti,ab** *OR* 'inter-tester':**ti,ab** *OR* 'intratester':**ti,ab** *OR* 'intratester':**ti,ab** *OR* 'interobeserver':**ti,ab** *OR* 'inter-observer':**ti,ab** *OR* 'intraobserver':**ti,ab** *OR* 'intraobserver':**ti,ab** *OR* 'intertechnician':**ti,ab** *OR* 'inter-technician':**ti,ab** *OR* 'intratechnician':**ti,ab** *OR* 'intratechnician':**ti,ab** *OR* 'interexaminer':**ti,ab** *OR* 'inter-examiner':**ti,ab** *OR* 'intraexaminer':**ti,ab** *OR* 'intraexaminer':**ti,ab** *OR* 'interassay':**ti,ab** *OR* 'inter-assay':**ti,ab** *OR* 'intraassay':**ti,ab** *OR* 'intra-assay':**ti,ab** *OR* 'interindividual':**ti,ab** *OR* 'inter-individual':**ti,ab** *OR* 'intraindividual':**ti,ab** *OR* 'intra-individual':**ti,ab** *OR* 'interparticipant':**ti,ab** *OR* 'inter-

participant’**:ti,ab** *OR* ‘intraparticipant’**:ti,ab** *OR* ‘intraparticipant’**:ti,ab** *OR* ‘kappa’**:ti,ab** *OR* ‘kappas’**:ti,ab** *OR* ‘coefficient of variation’**:ti,ab** *OR* repeatab***:ti,ab** *OR* (replicab***:ti,ab** *OR* ‘repeated’**:ti,ab** *AND* (‘measure’**:ti,ab** *OR* ‘measures’**:ti,ab** *OR* ‘findings’**:ti,ab** *OR* ‘result’**:ti,ab** *OR* ‘results’**:ti,ab** *OR* ‘test’**:ti,ab** *OR* ‘tests’**:ti,ab**)) *OR* generaliza***:ti,ab** *OR* generalisa***:ti,ab** *OR* ‘concordance’**:ti,ab** *OR* (‘intraclass’**:ti,ab** *AND* correlation***:ti,ab**) *OR* ‘discriminative’**:ti,ab** *OR* ‘known group’**:ti,ab** *OR* ‘factor analysis’**:ti,ab** *OR* ‘factor analyses’**:ti,ab** *OR* ‘factor structure’**:ti,ab** *OR* ‘factor structures’**:ti,ab** *OR* ‘dimensionality’**:ti,ab** *OR* subscale***:ti,ab** *OR* ‘multitrait scaling analysis’**:ti,ab** *OR* ‘multitrait scaling analyses’**:ti,ab** *OR* ‘item discriminant’**:ti,ab** *OR* ‘interscale correlation’**:ti,ab** *OR* ‘interscale correlations’**:ti,ab** *OR* (‘error’**:ti,ab** *OR* ‘errors’**:ti,ab** *AND* (measure***:ti,ab** *OR* correlat***:ti,ab** *OR* evaluat***:ti,ab** *OR* ‘accuracy’**:ti,ab** *OR* ‘accurate’**:ti,ab** *OR* ‘precision’**:ti,ab** *OR* ‘mean’**:ti,ab**)) *OR* ‘individual variability’**:ti,ab** *OR* ‘interval variability’**:ti,ab** *OR* ‘rate variability’**:ti,ab** *OR* ‘variability analysis’**:ti,ab** *OR* (‘uncertainty’**:ti,ab** *AND* (‘measurement’**:ti,ab** *OR* ‘measuring’**:ti,ab**)) *OR* ‘standard error of measurement’**:ti,ab** *OR* sensitiv***:ti,ab** *OR* responsive***:ti,ab** *OR* (‘limit’**:ti,ab** *AND* ‘detection’**:ti,ab**) *OR* ‘minimal detectable concentration’**:ti,ab** *OR* interpretab***:ti,ab** *OR* (small***:ti,ab** *AND* (‘real’**:ti,ab** *OR* ‘detectable’**:ti,ab**) *AND* (‘change’**:ti,ab** *OR* ‘difference’**:ti,ab**)) *OR* ‘meaningful change’**:ti,ab** *OR* ‘minimal important change’**:ti,ab** *OR* ‘minimal important difference’**:ti,ab** *OR* ‘minimally important change’**:ti,ab** *OR* ‘minimally important difference’**:ti,ab** *OR* ‘minimal detectable change’**:ti,ab** *OR* ‘minimal detectable difference’**:ti,ab** *OR* ‘minimally detectable change’**:ti,ab** *OR* ‘minimally detectable difference’**:ti,ab** *OR* ‘minimal real change’**:ti,ab** *OR* ‘minimal real difference’**:ti,ab** *OR* ‘minimally real change’**:ti,ab** *OR* ‘minimally real difference’**:ti,ab** *OR* ‘ceiling effect’**:ti,ab** *OR* ‘floor effect’**:ti,ab** *OR* ‘item response model’**:ti,ab** *OR* ‘irt’**:ti,ab** *OR* ‘rasch’**:ti,ab** *OR* ‘differential item functioning’**:ti,ab** *OR* ‘dif’**:ti,ab** *OR* ‘computer adaptive testing’**:ti,ab** *OR* ‘item bank’**:ti,ab** *OR* ‘cross-cultural equivalence’**:ti,ab**)

#5) **#1** ***AND*** **#2** ***AND*** **#3** ***AND*** **#4**

Cochrane

Note: If you reuse this search strategy, be careful when copying " or ' into the Cochrane Search Manager. Your text program may format it in a way the Search Manager cannot process. We recommend to manually transfer relevant sections.

#1) (((sexual* *OR* reproductive) *AND* (literacy *OR* literate *OR* numeracy *OR* knowledge *OR* belief* *OR* attitud* *OR* motivation* *OR* competenc* *OR* skills*)):**ti,ab,kw**)

#2) ((adolescen* *OR* teen* *OR* youth *OR* minor* *OR* young *OR* pupil* *OR* student*):**ti,ab,kw**)

#3) ((((self *OR* child *OR* parent *OR* carer *OR* proxy) *AND* ((report *OR* reported *OR* reporting) *OR* (rated *OR* rating *OR* ratings) *OR* based *OR* (assessed *OR* assessment *OR* assessments))) *OR* (outcome *OR* outcomes *OR* index *OR* indices *OR* instrument *OR* instruments *OR* measure *OR* measures *OR* questionnaire *OR* questionnaires *OR* profile *OR* profiles *OR* scale *OR* scales *OR* score *OR* scores *OR* status *OR* survey *OR* surveys)):**ti,ab**)

#4) (**[mh** methods**]** *OR* "comparative study":**ti,ab** *OR* **[mh** psychometrics**]** *OR* psychometr*:**ti,ab** *OR* clinimetr* *OR* clinometr* *OR* **[mh** "outcome assessment, health care"**]** *OR* "outcome assessment":**ti,ab** *OR* "outcome measure" *OR* **[mh** "observer variation"**]** *OR* "observer variation":**ti,ab** *OR* **[mh** "health status indicators"**]** *OR* **[mh** "reproducibility of results"**]** *OR* reproducib*:**ti,ab** *OR* **[mh** "discriminant analysis"**]** *OR* reliab*:**ti,ab** *OR* unreliab*:**ti,ab** *OR* valid*:**ti,ab** *OR* "coefficient of variation":**ti,ab** *OR* coefficient:**ti,ab** *OR* homogeneity:**ti,ab** *OR* homogeneous:**ti,ab** *OR* "internal consistency":**ti,ab** *OR* (cronbach*:**ti,ab** *AND* (alpha:**ti,ab** *OR* alphas:**ti,ab**)) *OR* (item:**ti,ab** *AND* (correlation*:**ti,ab** *OR* selection*:**ti,ab** *OR* reduction*:**ti,ab**)) *OR* agreement *OR* precision *OR* imprecision *OR* "precise values" *OR* test-retest:**ti,ab** *OR* (test:**ti,ab** *AND* retest:**ti,ab**) *OR* (reliab*:**ti,ab** *AND* (test:**ti,ab** *OR* retest:**ti,ab**)) *OR* stability:**ti,ab** *OR* interrater:**ti,ab** *OR* inter-rater:**ti,ab** *OR* intrarater:**ti,ab** *OR* intra-rater:**ti,ab** *OR* intertester:**ti,ab** *OR* inter-tester:**ti,ab** *OR* intratester:**ti,ab** *OR* intra-tester:**ti,ab** *OR* interobserver:**ti,ab** *OR* inter-observer:**ti,ab** *OR* intraobserver:**ti,ab** *OR* intra-observer:**ti,ab** *OR* intertechnician:**ti,ab** *OR* inter-technician:**ti,ab** *OR* intratechnician:**ti,ab** *OR* intra-technician:**ti,ab** *OR* interexaminer:**ti,ab** *OR* inter-examiner:**ti,ab** *OR* intraexaminer:**ti,ab** *OR* intra-examiner:**ti,ab** *OR* interassay:**ti,ab** *OR* inter-assay:**ti,ab** *OR* intraassay:**ti,ab** *OR* intra-assay:**ti,ab** *OR* interindividual:**ti,ab** *OR* inter-individual:**ti,ab** *OR* intraindividual:**ti,ab** *OR* intra-individual:**ti,ab** *OR* interparticipant:**ti,ab** *OR* inter-participant:**ti,ab** *OR* intraparticipant:**ti,ab** *OR* intra-participant:**ti,ab** *OR* kappa:**ti,ab** *OR* kappa's:**ti,ab** *OR* kappas:**ti,ab** *OR* repeatab* *OR* ((replicab* *OR* repeated) *AND* (measure *OR*

measures *OR* findings *OR* result *OR* results *OR* test *OR* tests)) *OR* generaliza*:**ti,ab** *OR* generalisa*:**ti,ab** *OR* concordance:**ti,ab** *OR* (intraclass:**ti,ab** *AND* correlation*:**ti,ab**) *OR* discriminative:**ti,ab** *OR* "known group":**ti,ab** *OR* "factor analysis":**ti,ab** *OR* "factor analyses":**ti,ab** *OR* "factor structure":**ti,ab** *OR* "factor structures":**ti,ab** *OR* dimension*:**ti,ab** *OR* subscale*:**ti,ab** *OR* (multitrait:**ti,ab** *AND* scaling:**ti,ab** *AND* (analysis:**ti,ab** *OR* analyses:**ti,ab**)) *OR* "item discriminant":**ti,ab** *OR* "interscale correlation":**ti,ab** *OR* "interscale correlations":**ti,ab** *OR* error:**ti,ab** *OR* errors:**ti,ab** *OR* "individual variability":**ti,ab** *OR* "interval variability":**ti,ab** *OR* "rate variability":**ti,ab** *OR* (variability:**ti,ab** *AND* (analysis:**ti,ab** *OR* values:**ti,ab**)) *OR* (uncertainty:**ti,ab** *AND* (measurement:**ti,ab** *OR* measuring:**ti,ab**)) *OR* "standard error of measurement":**ti,ab** *OR* sensitiv*:**ti,ab** *OR* responsive*:**ti,ab** *OR* (limit:**ti,ab** *AND* detection:**ti,ab**) *OR* "minimal detectable concentration":**ti,ab** *OR* interpretab*:**ti,ab** *OR* ((minimal:**ti,ab** *OR* minimally:**ti,ab** *OR* clinical:**ti,ab** *OR* clinically:**ti,ab**) *AND* (important:**ti,ab** *OR* significant:**ti,ab** *OR* detectable:**ti,ab**) *AND* (change:**ti,ab** *OR* difference:**ti,ab**)) *OR* (small*:**ti,ab** *AND* (real:**ti,ab** *OR* detectable:**ti,ab**) *AND* (change:**ti,ab** *OR* difference:**ti,ab**)) *OR* "meaningful change":**ti,ab** *OR* "ceiling effect":**ti,ab** *OR* "floor effect":**ti,ab** *OR* "Item response model":**ti,ab** *OR* IRT:**ti,ab** *OR* Rasch:**ti,ab** *OR* "Differential item functioning":**ti,ab** *OR* DIF:**ti,ab** *OR* "computer adaptive testing":**ti,ab** *OR* "item bank":**ti,ab** *OR* "cross-cultural equivalence":**ti,ab**)

#5) #1 AND #2 AND #3 AND #4

PsycInfo, PSYNDEX, PsycArticle (via EBSCO)

#1) (**TI** ((sexual* *OR* reproductive) *AND* (literacy *OR* literate *OR* numeracy *OR* knowledge *OR* belief* *OR* attitud* *OR* motivation* *OR* competenc* *OR* skills*)) *OR* **AB** ((sexual* *OR* reproductive) *AND* (literacy *OR* literate *OR* numeracy *OR* knowledge *OR* belief* *OR* attitud* *OR* motivation* *OR* competenc* *OR* skills*)) *OR* **SU** ((sexual* *OR* reproductive) *AND* (literacy *OR* literate *OR* numeracy *OR* knowledge *OR* belief* *OR* attitud* *OR* motivation* *OR* competenc* *OR* skills*)))

#2) (**TI** (adolescen* *OR* teen* *OR* youth *OR* minor* *OR* young *OR* pupil* *OR* student*) *OR* **AB** (adolescen* *OR* teen* *OR* youth *OR* minor* *OR* young *OR* pupil* *OR* student*) *OR* **SU** (adolescen* *OR* teen* *OR* youth *OR* minor* *OR* young *OR* pupil* *OR* student*)

#3) (**TI** (((self *OR* child *OR* parent *OR* carer *OR* proxy) *AND* ((report *OR* reported *OR* reporting) *OR* (rated *OR* rating *OR* ratings) *OR* based *OR* (assessed *OR* assessment *OR* assessments))) *OR* (outcome *OR* outcomes *OR* index *OR* indices *OR* instrument *OR* instruments *OR* measure *OR* measures *OR* questionnaire *OR* questionnaires *OR* profile *OR* profiles *OR* scale *OR* scales *OR* score *OR* scores *OR* status *OR* survey *OR* surveys)) *OR* **AB** (((self *OR* child *OR* parent *OR* carer *OR* proxy) *AND* ((report *OR* reported *OR* reporting) *OR* (rated *OR* rating *OR* ratings) *OR* based *OR* (assessed *OR* assessment *OR* assessments))) *OR* (outcome *OR* outcomes *OR* index *OR* indices *OR* instrument *OR* instruments *OR* measure *OR* measures *OR* questionnaire *OR* questionnaires *OR* profile *OR* profiles *OR* scale *OR* scales *OR* score *OR* scores *OR* status *OR* survey *OR* surveys))

#4) (**TI** (instrumentation *OR* "comparative study" *OR* psychometr* *OR* "outcome assessment" *OR* "observer variation" *OR* "health status indicators" *OR* "reproducibility of results" *OR* reproducib* *OR* "discriminant analysis" *OR* reliab* *OR* unreliab* *OR* valid* *OR* "coefficient of variation" *OR* coefficient *OR* homogeneity *OR* homogeneous *OR* "internal consistency" *OR* (cronbach* *AND* (alpha *OR* alphas)) *OR* (item *AND* (correlation* *OR* selection* *OR* reduction*)) *OR* test-retest *OR* (test *AND* retest) *OR* (reliab* *AND* (test *OR* retest)) *OR* stability *OR* interrater *OR* inter-rater *OR* intrarater *OR* intra-rater *OR* intertester *OR* inter-tester *OR* intratester *OR* intra-tester *OR* interobserver *OR* inter-observer *OR* intraobserver *OR* intra-observer *OR* intertechnician *OR* inter-technician *OR* intratechnician *OR* intra-technician *OR* interexaminer *OR* inter-examiner *OR* intraexaminer *OR* intra-examiner *OR* interassay *OR* inter-assay *OR* intraassay *OR* intra-assay *OR* interindividual *OR* inter-individual *OR* intraindividual *OR* intra-individual *OR* interparticipant *OR* inter-participant *OR* intraparticipant *OR* intra-participant *OR* kappa *OR* kappa's *OR* kappas *OR* generaliza* *OR* generalisa* *OR* concordance *OR* (intraclass *AND* correlation*) *OR* discriminative *OR* "known group" *OR* "factor analysis" *OR* "factor analyses" *OR*

"factor structure" *OR* "factor structures" *OR* dimension* *OR* subscale* *OR* (multitrait *AND* scaling *AND* (analysis *OR* analyses)) *OR* "item discriminant" *OR* "interscale correlation" *OR* "interscale correlations" *OR* error *OR* errors *OR* "individual variability" *OR* "interval variability" *OR* "rate variability" *OR* (variability *AND* (analysis *OR* values)) *OR* (uncertainty *AND* (measurement *OR* measuring)) *OR* "standard error of measurement" *OR* sensitiv* *OR* responsive* *OR* (limit *AND* detection) *OR* "minimal detectable concentration" *OR* interpretab* *OR* ((minimal *OR* minimally *OR* clinical *OR* clinically) *AND* (important *OR* significant *OR* detectable) *AND* (change *OR* difference)) *OR* (small* *AND* (real *OR* detectable) *AND* (change *OR* difference)) *OR* "meaningful change" *OR* "ceiling effect" *OR* "floor effect" *OR* "Item response model" *OR* IRT *OR* Rasch *OR* "Differential item functioning" *OR* DIF *OR* "computer adaptive testing" *OR* "item bank" *OR* "cross-cultural equivalence") *OR* **AB** (instrumentation *OR* "comparative study" *OR* psychometr* *OR* "outcome assessment" *OR* "observer variation" *OR* "health status indicators" *OR* "reproducibility of results" *OR* reproducib* *OR* "discriminant analysis" *OR* reliab* *OR* unreliab* *OR* valid* *OR* "coefficient of variation" *OR* coefficient *OR* homogeneity *OR* homogeneous *OR* "internal consistency" *OR* (cronbach* *AND* (alpha *OR* alphas)) *OR* (item *AND* (correlation* *OR* selection* *OR* reduction*)) *OR* test-retest *OR* (test *AND* retest) *OR* (reliab* *AND* (test *OR* retest)) *OR* stability *OR* interrater *OR* inter-rater *OR* intrarater *OR* intra-rater *OR* intertester *OR* inter-tester *OR* intratester *OR* intra-tester *OR* interobserver *OR* inter-observer *OR* intraobserver *OR* intra-observer *OR* intertechnician *OR* inter-technician *OR* intratechnician *OR* intra-technician *OR* interexaminer *OR* inter-examiner *OR* intraexaminer *OR* intra-examiner *OR* interassay *OR* inter-assay *OR* intraassay *OR* intra-assay *OR* interindividual *OR* inter-individual *OR* intraindividual *OR* intra-individual *OR* interparticipant *OR* inter-participant *OR* intraparticipant *OR* intra-participant *OR* kappa *OR* kappa's *OR* kappas *OR* generaliza* *OR* generalisa* *OR* concordance *OR* (intraclass *AND* correlation*) *OR* discriminative *OR* "known group" *OR* "factor analysis" *OR* "factor analyses" *OR* "factor structure" *OR* "factor structures" *OR* dimension* *OR* subscale* *OR* (multitrait *AND* scaling *AND* (analysis *OR* analyses)) *OR* "item discriminant" *OR* "interscale correlation" *OR* "interscale correlations" *OR* error *OR* errors *OR* "individual variability" *OR* "interval variability" *OR* "rate variability" *OR* (variability *AND* (analysis *OR* values)) *OR* (uncertainty *AND* (measurement *OR* measuring)) *OR* "standard error of measurement" *OR* sensitiv* *OR* responsive* *OR* (limit *AND* detection) *OR* "minimal detectable concentration" *OR* interpretab* *OR* ((minimal *OR* minimally *OR* clinical *OR* clinically) *AND* (important *OR* significant *OR* detectable) *AND* (change *OR* difference)) *OR* (small* *AND* (real *OR* detectable) *AND* (change *OR* difference)) *OR* "meaningful change" *OR* "ceiling effect" *OR* "floor effect" *OR* "Item response model" *OR* IRT *OR* Rasch *OR* "Differential item functioning" *OR* DIF *OR* "computer adaptive testing" *OR* "item bank" *OR* "cross-cultural equivalence")

OR **(**clinimetr* *OR* clinometr* *OR* “outcome measure” *OR* agreement *OR* precision *OR* imprecision *OR* “precise values” *OR* repeatab* *OR* ((replicab* *OR* repeated) *AND* (measure *OR* measures *OR* findings *OR* result *OR* results *OR* test *OR* tests**)**)))

#5)   ***#1 AND #2 AND #3 AND #4***

ERIC

Maximum search strategy length: 1700 characters; additional filter applied: peer review only

title:((sexual* *OR* reproductive) *AND* (literacy *OR* literate *OR* numeracy *OR* knowledge *OR* belief* *OR* attitud* *OR* motivation* *OR* competenc* *OR* skills*)) *OR* **abstract**:((sexual* *OR* reproductive) *AND* (literacy *OR* literate *OR* numeracy *OR* knowledge *OR* belief* *OR* attitud* *OR* motivation* *OR* competenc* *OR* skills*)) *OR* **keyword**:((sexual* *OR* reproductive) *AND* (literacy *OR* literate *OR* numeracy *OR* knowledge *OR* belief* *OR* attitud* *OR* motivation* *OR* competenc* *OR* skills*)) *AND* **title**:(adolescen* *OR* teen* *OR* youth *OR* minor* *OR* young *OR* pupil* *OR* student*) *OR* **abstract**:((adolescen* *OR* teen* *OR* youth *OR* minor* *OR* young *OR* pupil* *OR* student*) *OR* **keyword**:((adolescen* *OR* teen* *OR* youth *OR* minor* *OR* young *OR* pupil* *OR* student*) *AND* **title**:(((self *OR* child *OR* parent *OR* carer *OR* proxy) *AND* ((report *OR* reported *OR* reporting) *OR* (rated *OR* rating *OR* ratings) *OR* based *OR* (assessed *OR* assessment *OR* assessments))) *OR* (outcome *OR* outcomes *OR* index *OR* indices *OR* instrument *OR* instruments *OR* measure *OR* measures *OR* questionnaire *OR* questionnaires *OR* profile *OR* profiles *OR* scale *OR* scales *OR* score *OR* scores *OR* status *OR* survey *OR* surveys)) *OR* **abstract**:(((self *OR* child *OR* parent *OR* carer *OR* proxy) *AND* ((report *OR* reported *OR* reporting) *OR* (rated *OR* rating *OR* ratings) *OR* based *OR* (assessed *OR* assessment *OR* assessments))) *OR* (outcome *OR* outcomes *OR* index *OR* indices *OR* instrument *OR* instruments *OR* measure *OR* measures *OR* questionnaire *OR* questionnaires *OR* profile *OR* profiles *OR* scale *OR* scales *OR* score *OR* scores *OR* status *OR* survey *OR* surveys)) AND **pubyearmin**:2002

Web of Science

#1) ((**TI**=((sexual* *OR* reproductive) *AND* (literacy *OR* literate *OR* numeracy *OR* knowledge *OR* belief* *OR* attitud* *OR* motivation* *OR* competenc* *OR* skills*))) *OR* (**AB**=((sexual* *OR* reproductive) *AND* (literacy *OR* literate *OR* numeracy *OR* knowledge *OR* belief* *OR* attitud* *OR* motivation* *OR* competenc* *OR* skills*))) *OR* (**AK**=((sexual* *OR* reproductive) *AND* (literacy *OR* literate *OR* numeracy *OR* knowledge *OR* belief* *OR* attitud* *OR* motivation* *OR* competenc* *OR* skills*))))

#2) ((**TI**=(adolescen* *OR* teen* *OR* youth *OR* minor* *OR* young *OR* pupil* *OR* student*)) *OR* (**AB**=(adolescen* *OR* teen* *OR* youth *OR* minor* *OR* young *OR* pupil* *OR* student*)) *OR* (**AK**=(adolescen* *OR* teen* *OR* youth *OR* minor* *OR* young *OR* pupil* *OR* student*)))

#3) ((**TI**=(((self *OR* child *OR* parent *OR* carer *OR* proxy) *AND* ((report *OR* reported *OR* reporting) *OR* (rated *OR* rating *OR* ratings) *OR* based *OR* (assessed *OR* assessment *OR* assessments))) *OR* (outcome *OR* outcomes *OR* index *OR* indices *OR* instrument *OR* instruments *OR* measure *OR* measures *OR* questionnaire *OR* questionnaires *OR* profile *OR* profiles *OR* scale *OR* scales *OR* score *OR* scores *OR* status *OR* survey *OR* surveys))) *OR* (**AB**=(((self *OR* child *OR* parent *OR* carer *OR* proxy) *AND* ((report *OR* reported *OR* reporting) *OR* (rated *OR* rating *OR* ratings) *OR* based *OR* (assessed *OR* assessment *OR* assessments))) *OR* (outcome *OR* outcomes *OR* index *OR* indices *OR* instrument *OR* instruments *OR* measure *OR* measures *OR* questionnaire *OR* questionnaires *OR* profile *OR* profiles *OR* scale *OR* scales *OR* score *OR* scores *OR* status *OR* survey *OR* surveys))))

#4) (**TI**=(instrumentation *OR* "comparative study" *OR* psychometr* *OR* "outcome assessment" *OR* "observer variation" *OR* "health status indicators" *OR* "reproducibility of results" *OR* reproducib* *OR* "discriminant analysis" *OR* reliab* *OR* unreliab* *OR* valid* *OR* "coefficient of variation" *OR* coefficient *OR* homogeneity *OR* homogeneous *OR* "internal consistency" *OR* (cronbach* *AND* (alpha *OR* alphas)) *OR* (item *AND* (correlation* *OR* selection* *OR* reduction*)) *OR* test-retest *OR* (test *AND* retest) *OR* (reliab* *AND* (test *OR* retest)) *OR* stability *OR* interrater *OR* inter-rater *OR* intrarater *OR* intra-rater *OR* intertester *OR* inter-tester *OR* intratester *OR* intra-tester *OR* interobserver *OR* inter-observer *OR* intraobserver *OR* intra-observer *OR* intertechnician *OR* inter-technician *OR* intratechnician *OR* intra-technician *OR* interexaminer *OR* inter-examiner *OR* intraexaminer *OR* intra-examiner *OR* interassay *OR* inter-assay *OR* intraassay *OR* intra-assay *OR* interindividual *OR* inter-individual *OR* intraindividual *OR* intra-individual *OR* interparticipant *OR* inter-participant *OR* intraparticipant *OR* intra-participant *OR* kappa *OR* kappa’s *OR* kappas *OR* generaliza* *OR* generalisa* *OR* concordance *OR* (intraclass *AND* correlation*) *OR* discriminative *OR* "known group" *OR* "factor

analysis" *OR* "factor analyses" *OR* "factor structure" *OR* "factor structures" *OR* dimension* *OR* subscale* *OR* (multitrait *AND* scaling *AND* (analysis *OR* analyses)) *OR* "item discriminant" *OR* "interscale correlation" *OR* "interscale correlations" *OR* error *OR* errors *OR* "individual variability" *OR* "interval variability" *OR* "rate variability" *OR* (variability *AND* (analysis *OR* values)) *OR* (uncertainty *AND* (measurement *OR* measuring)) *OR* "standard error of measurement" *OR* sensitiv* *OR* responsive* *OR* (limit *AND* detection) *OR* "minimal detectable concentration" *OR* interpretab* *OR* ((minimal *OR* minimally *OR* clinical *OR* clinically) *AND* (important *OR* significant *OR* detectable) *AND* (change *OR* difference)) *OR* (small* *AND* (real *OR* detectable) *AND* (change *OR* difference)) *OR* "meaningful change" *OR* "ceiling effect" *OR* "floor effect" *OR* "Item response model" *OR* IRT *OR* Rasch *OR* "Differential item functioning" *OR* DIF *OR* "computer adaptive testing" *OR* "item bank" *OR* "cross-cultural equivalence") *OR* **AB**=(instrumentation *OR* "comparative study" *OR* psychometr* *OR* "outcome assessment" *OR* "observer variation" *OR* "health status indicators" *OR* "reproducibility of results" *OR* reproducib* *OR* "discriminant analysis" *OR* reliab* *OR* unreliab* *OR* valid* *OR* "coefficient of variation" *OR* coefficient *OR* homogeneity *OR* homogeneous *OR* "internal consistency" *OR* (cronbach* *AND* (alpha *OR* alphas)) *OR* (item *AND* (correlation* *OR* selection* *OR* reduction*)) *OR* test-retest *OR* (test *AND* retest) *OR* (reliab* *AND* (test *OR* retest)) *OR* stability *OR* interrater *OR* inter-rater *OR* intrarater *OR* intra-rater *OR* intertester *OR* inter-tester *OR* intratester *OR* intra-tester *OR* interobserver *OR* inter-observer *OR* intraobserver *OR* intra-observer *OR* intertechnician *OR* inter-technician *OR* intratechnician *OR* intra-technician *OR* interexaminer *OR* inter-examiner *OR* intraexaminer *OR* intra-examiner *OR* interassay *OR* inter-assay *OR* intraassay *OR* intra-assay *OR* interindividual *OR* inter-individual *OR* intraindividual *OR* intra-individual *OR* interparticipant *OR* inter-participant *OR* intraparticipant *OR* intra-participant *OR* kappa *OR* kappa's *OR* kappas *OR* generaliza* *OR* generalisa* *OR* concordance *OR* (intraclass *AND* correlation*) *OR* discriminative *OR* "known group" *OR* "factor analysis" *OR* "factor analyses" *OR* "factor structure" *OR* "factor structures" *OR* dimension* *OR* subscale* *OR* (multitrait *AND* scaling *AND* (analysis *OR* analyses)) *OR* "item discriminant" *OR* "interscale correlation" *OR* "interscale correlations" *OR* error *OR* errors *OR* "individual variability" *OR* "interval variability" *OR* "rate variability" *OR* (variability *AND* (analysis *OR* values)) *OR* (uncertainty *AND* (measurement *OR* measuring)) *OR* "standard error of measurement" *OR* sensitiv* *OR* responsive* *OR* (limit *AND* detection) *OR* "minimal detectable concentration" *OR* interpretab* *OR* ((minimal *OR* minimally *OR* clinical *OR* clinically) *AND* (important *OR* significant *OR* detectable) *AND* (change *OR* difference)) *OR* (small* *AND* (real *OR* detectable) *AND* (change *OR* difference)) *OR* "meaningful change" *OR* "ceiling effect" *OR* "floor effect" *OR* "Item response model" *OR* IRT *OR* Rasch *OR* "Differential item functioning" *OR* DIF *OR* "computer adaptive testing" *OR* "item

bank" *OR* "cross-cultural equivalence") *OR* **ALL**=(clinimetr* *OR* clinometr* *OR* "outcome measure" *OR* agreement *OR* precision *OR* imprecision *OR* "precise values" *OR* repeatab* *OR* ((replicab* *OR* repeated) *AND* (measure *OR* measures *OR* findings *OR* result *OR* results *OR* test *OR* tests))))

#5) **DT**=(Article *OR* Review)

#6) #1 *AND* #2 *AND* #3 *AND* #4 *AND* #5

ASSIA – Applied Social Sciences Index & Abstracts (via ProQuest)

noft(((sexual* *OR* reproductive) *AND* (literacy *OR* literate *OR* numeracy *OR* knowledge *OR* belief* *OR* attitud* *OR* motivation* *OR* competenc* *OR* skills*)) *AND* (adolescen* *OR* teen* *OR* youth *OR* minor* *OR* young *OR* pupil* *OR* student*) *AND* (((self *OR* child *OR* parent *OR* carer *OR* proxy) *AND* ((report *OR* reported *OR* reporting) *OR* (rated *OR* rating *OR* ratings) *OR* based *OR* (assessed *OR* assessment *OR* assessments))) *OR* (outcome *OR* outcomes *OR* index *OR* indices *OR* instrument *OR* instruments *OR* measure *OR* measures *OR* questionnaire *OR* questionnaires *OR* profile *OR* profiles *OR* scale *OR* scales *OR* score *OR* scores *OR* status *OR* survey *OR* surveys))**)** *AND* (**MESH(**instrumentation *OR* methods**)** *OR* **noft(**psychometrics *OR* "observer variation" *OR* "health status indicators" *OR* "reproducibility of results" *OR* "discriminant analysis" *OR* „comparative study" *OR* psychometr* *OR* "outcome assessment" *OR* "observer variation" *OR* reproducib* *OR* reliab* *OR* unreliab* *OR* valid* *OR* "coefficient of variation" *OR* coefficient *OR* homogeneity *OR* homogeneous *OR* "internal consistency" *OR* (cronbach* *AND* (alpha *OR* alphas)) *OR* (item *AND* (correlation* *OR* selection* *OR* reduction*)) *OR* test-retest *OR* (test *AND* retest) *OR* (reliab* *AND* (test *OR* retest)) *OR* stability *OR* interrater *OR* inter-rater *OR* intrarater *OR* intra-rater *OR* intertester *OR* inter-tester *OR* intratester *OR* intra-tester *OR* interobserver *OR* inter-observer *OR* intraobserver *OR* intra-observer *OR* intertechnician *OR* inter-technician *OR* intratechnician *OR* intra-technician *OR* interexaminer *OR* inter-examiner *OR* intraexaminer *OR* intra-examiner *OR* interassay *OR* inter-assay *OR* intraassay *OR* intra-assay *OR* interindividual *OR* inter-individual *OR* intraindividual *OR* intra-individual *OR* interparticipant *OR* inter-participant *OR* intraparticipant *OR* intra-participant *OR* kappa *OR* kappa's *OR* kappas *OR* generaliza* *OR* generalisa* *OR* concordance *OR* (intraclass *AND* correlation*) *OR* discriminative *OR* "known group" *OR* "factor analysis" *OR* "factor analyses" *OR* "factor structure" *OR* "factor structures" *OR* dimension* *OR* subscale* *OR* (multitrait *AND* scaling *AND* (analysis *OR* analyses)) *OR* "item discriminant" *OR* "interscale correlation" OR "interscale correlations" OR error *OR* errors *OR* "individual variability" *OR* "interval variability" *OR* "rate variability" *OR* (variability *AND* (analysis *OR* values)) *OR* (uncertainty *AND* (measurement *OR* measuring)) *OR* "standard error of measurement" *OR* sensitiv* *OR* responsive* *OR* (limit *AND* detection) *OR* "minimal detectable concentration" *OR* interpretab* *OR* ((minimal *OR* minimally *OR* clinical *OR* clinically) *AND* (important *OR* significant *OR* detectable) *AND* (change *OR* difference)) *OR* (small* *AND* (real *OR* detectable) *AND* (change *OR* difference)) *OR* "meaningful change" *OR* "ceiling effect" *OR* "floor effect" *OR* "Item response model" *OR* IRT *OR* Rasch *OR* "Differential item functioning" *OR* DIF *OR* "computer adaptive testing" *OR* "item bank" *OR* "cross-cultural equivalence"**)** *OR* **(**clinimetr* *OR* clinometr* *OR* "outcome

measure” *OR* agreement *OR* precision *OR* imprecision *OR* “precise values” *OR* repeatab* *OR* ((replicab* *OR* repeated) *AND* (measure *OR* measures *OR* findings *OR* result *OR* results *OR* test *OR* tests))**)**)
