# Supplementary material for: A COSMIN Systematic Review of Sexual Health Literacy Self-Report Measures for Adolescents
Source: Arch Sex Behav. 2025 Jun 6;54(5):1737–68. doi: 10.1007/s10508-025-03142-1 (PMC12162768; doi:10.1007/s10508-025-03142-1)
Supplement: Supplementary file 2 — Supplementary file2 (PDF 71 KB) [file 10508_2025_3142_MOESM2_ESM.pdf]

Inclusion and Exclusion Criteria

	Inclusion	Exclusion
**Construct of interest**	OMI aims to measure<br>• Sexual Health Literacy,<br>• Sexual Health knowledge,<br>• attitudes, beliefs and motivations towards sexuality and relationships<br>• or sexual (risk) behavior (competences, skills)	No aspects of SHL are measured by the OMI
**Population of interest**	Study sample (or at least an analyzed subgroup) represents Adolescents (10-19 years old; World Health Organization [WHO], 2001), all genders (also minority groups)	Study sample represents only<br>• Children under 10 years,<br>• (Young) Adults (study populations 18-25, 18+, mean age ≥ 19, undergraduate/university students),<br>• target groups with specific health conditions (e.g. obesity, asthma, autism-spectrum disorders),<br>• sex workers, health care providers<br><br>The OMI studied was not developed for the population of interest and has not been adapted for that group
**Study Aim**	• evaluation of one or more measurement properties,<br>• development of an OMI<br>• or the evaluation of the interpretability of the OMIs of interest	The study does not concern self-report OMIs, study only uses the OMI as an outcome measurement instrument
**Time span**	The OMI was developed, updated or adapted in the time span since 2002 until today	The OMI was developed before 2002 and was not updated or adapted since then
**Language(s)**	Englisch, German	Other Languages
**Type of Study**	Peer-reviewed publications	Non-peer-reviewed publications, editorials, conference abstracts, commentaries, book chapters, study protocols, dissertations, theses, grey literature

Note. OMI=Outcome Measurement Instrument

References
World Health Organization (WHO). (2001). *The Second Decade: Improving Adolescent Health and Development* (No. WHO/FRH/ADH/98.18 Rev. 1). https://apps.who.int/iris/handle/10665/64320
