# Supplementary material for: A COSMIN Systematic Review of Sexual Health Literacy Self-Report Measures for Adolescents
Source: Arch Sex Behav. 2025 Jun 6;54(5):1737–68. doi: 10.1007/s10508-025-03142-1 (PMC12162768; doi:10.1007/s10508-025-03142-1)
Supplement: Supplementary file 3 — Supplementary file3 (PDF 100 KB) [file 10508_2025_3142_MOESM3_ESM.pdf]

Sexual Health Literacy

Sample Items

Dimension		Definition	Sample Item
Access	a) Healthcare	Ability to access information on medical and clinical issues (e.g. abortion, pregnancy)	*I feel comfortable talking about my sexual and reproductive health concerns with healthcare professionals. (Ghiasi et al. 2022)*<br><br>*I have received information about nutrition during pregnancy, sex during pregnancy, oral health care during pregnancy, childbirth preparation classes, how to take iron supplement, benets of taking iron and multivitamins during pregnancy from health care providers. (Ghiasi et al. 2022)*<br><br>*As soon as I know I am pregnant, make first prenatal appointment with a doctor or midwife. (Ghiasi et al 2022)*<br><br>*When I go for a general medical checkup, my regular pediatrician or family doctor does not know I am transgender. (Fisher et al. 2018)*<br><br>*When I go for a medical checkup, my regular pediatrician or family doctor is helpful about sexual health issues specifically for transgender individuals. (Fisher et al. 2018)*<br><br>*How often do you worry that you might already have the HIV virus? (Sales et al. 2009)*
	a) Disease Prevention	Ability to access information on risk factors for SH (e.g. condom use, contraception, pearl index)	*I do not ask my regular doctor for information about condoms or other ways to prevent HIV/STIs because I worry that my doctor would tell my parents I was sexually active. (Fisher et al. 2018)*<br><br>*Have you ever been tested for a STD or HIV? (Yes, No) (Jerman et al. 2015)*<br><br>*Have you ever been to a health centre, clinic or doctor for sexual health services (like birth control, pregnancy tests or sexually transmitted disease [STD] tests)? (Jerman et al. 2015)*<br><br>*How often do you worry that you might get the HIV virus? (Sales et al. 2009)*
	a) Health Promotion	Ability to update oneself on determinants of SH in the social and physical environment (e.g. conversation with parents on sex and relationships)	*If you had an important concern about drugs, alcohol, sex, or some other serious issue, would you talk to your parents or other adults in your household about it? (Reininger et al. 2004)*<br><br>*I have had lots of good conversations with my parents or other adults in my household about waiting until I am older, or married, to have sexual intercourse (Reininger et al. 2004)*<br><br>*I feel comfortable talking about sexual and reproductive health issues with my mother. (Ghiasi et al. 2022)*<br><br>*The media is a good way to learn about Sexual Assault (Edwards et al. 2015)*<br><br>*I get information about relationships from the media (Edwards et al. 2015)*<br><br>*My parents give me enough training and guidance on the subject of AIDS. (Darabi et al. 2018)*
Understand	b) Healthcare	Ability to understand medical information and derive meaning	*Menstruation is one form of disease (Zakaria et al. 2020)*<br><br>*Correct medication can decrease viral load. (Yau et al. 2020)*<br><br>*When to get tested for HIV/STIs or pregnancy. (Yau et al. 2020)*<br><br>*Unsafe abortion can result in many health-damaging consequences. (Yau et al. 2020)*<br><br>*Having sex during pregnancy is generally safe and won't hurt the fetus. (Ghiasi et al. 2022)*<br><br>*The rectum makes enough lubrication for anal penetration.* (Kutner et al. 2022)*<br><br>*STIs may cause infertility. (Acharya et al. 2016)*

Dimension		Definition	Sample Item
	b) Disease Prevention	Ability to understand information on risk factors and derive meaning	*HIV does not spread the virus from an infected person's coughing and sneezing (Zakaria et al. 2020)*<br><br>*HIV is not transmittable by sleeping in the same room with an infected person. (Yau et al. 2020)*<br><br>*AIDS is caused by a virus called "HIV". (Abello-Luque et al. 2021)*<br><br>*Pregnancy can happen if semen is spilled outside the vagina (such as in intercrural sex, or anal sex). (Ghiasi et al. 2022)*<br><br>*The only time that one should use a condom is when you have sex with someone for the first time* (Aarø et al. 2001)*<br><br>*Using two condoms at the same time works better than using one.* (Jerman et al. 2015)*
	b) Health Promotion	Ability to understand information on determinants of SH in the social and physical environment and derive meaning	*I think homo and bisexuality are unnatural.* (Garrido-Hernansaiz et al. 2017)*<br><br>*In my opinion, homosexuality is not an illness. (Garrido-Hernansaiz et al. 2017)*<br><br>*Do you think once a guy is sexually excited, it would be harmful if he didn't ejaculate (come)? (Deardorff et al. 2008)*
Appraise	c) Healthcare	Ability to interpret and evaluate medical information	*If in a couple the woman becomes pregnant, they should have an abortion. (Barros et al. 2020)*<br><br>*Having a baby now would be too much of a burden on me. (Steven Simons et al. 2005)*<br><br>*Sexually transmitted diseases are a very serious health problem for people my age (Mũkoma et al. 2009)*<br><br>*People with AIDS should be kept away from school. (Darabie et al. 2018)*
	c) Disease Prevention	Ability to interpret and evaluate information on risk factors for SH	*I think it's ok for unmarried teenagers to have sexual intercourse if they use birth control (Reininger et al. 2004)*<br><br>*All STDs are preventable. (Yau et al. 2020)*<br><br>*Having sex at school age is wrong, it might lead to pregnancy and/or infection. (Yau et al. 2020)*<br><br>*Drinking alcohol can lead to premarital sex at school age. (Yau et al. 2020)*<br><br>*If a friend became infected with HIV, I probably would distance myself from him (Espada et al. 2013)*<br><br>*Using a condom is a way of expressing responsibility for my partner and myself (Mũkoma et al. 2009)*<br><br>*Girl can suggest boy to use condom if he is suffered from STI (Acharya et al. 2016)*<br><br>*A person who is in a sexual relationship with a steady partner (like a boyfriend or girlfriend) always has the right insist on using condoms or birth control (Berglas et al. 2016)*<br><br>*Asking somebody to use condoms presumes distrust (Escribano et al. 2017)*

Dimension		Definition	Sample Item
	**c) Health Promotion**	Ability to interpret and evaluate information on SH determinants in the social and physical environment	*Masturbation is a sign of weak willpower/self-control (Ren et al. 2022)*<br><br>*It's ok for a guy to say no to sex. (Chu et al. 2005)*<br><br>*In a good dating relationship, the guy gets his way most of the time.* (Chu et al. 2005)*<br><br>*Guys should not let it show when their feelings are hurt.* (Chu et al. 2005)*<br><br>*Guys should sleep with as many girls as possible.* (Hill et al. 2021)*<br><br>*I have a right to know where my partner is all the time* (Hannawa et al. 2006)*<br><br>*If a man is providing a lot of financial support then he should decide whether or not they use a condom during sex* (Stoebenau et al. 2022)*<br><br>*As a woman, it's important to know how to use physical beauty to get whatever you want from a man (Stoebenau et al. 2022)*<br><br>*Having sex at school age can be done because my peers do it.* (Yau et al 2020)*<br><br>*Sex is nasty (O'Sullivan et al. 2006)*<br><br>*Same-sex marriages sholud not be allowed.* (Garrido-Hernansaiz et al. 2017)*<br><br>*Sexual Assault charges are often used as a way of getting back at guys.* (Edwards et al. 2015)*<br><br>*I don't think there is much I can do about abuse and Sexual Assault (Edwards et al. 2015)*<br><br>*Is it okay for a girl to talk about sex with a guy when they know each other but aren't dating?* (Deardorff et al. 2008)*<br><br>*Do you think it's wrong for you to have sex?* (Deardorff et al. 2008)*<br><br>*A person who is in a sexual relationship with a steady partner (like a boyfriend or girlfriend) always has the right to say no to sex (Berglas et al. 2016)*<br><br>*I am aware that students at my school are sexually harassed. (Nickerson et al. 2014)*
**Apply**	**d) Healthcare**	Ability to make informed decisions on medical issues	*I can persuade my husband to pay attention to my ideas about timing and number of births. (Ghiasi et al. 2022)*<br><br>*I would be willing to be tested for AIDS if I had had a risk behavior. (Espada et al. 2013)*<br><br>*How would you feel talking about what you would do about a pregnancy (like keep the baby, have an abortion, etc.)? (Deardorff et al. 2008)*

Dimension		Definition	Sample Item
	d) Disease Prevention	Ability to make informed decisions on risk factors for SH	*If I were to have sex before marriage, I or my partner would use birth control every time (Reininger et al. 2004)*<br><br>*While sexting, I am not embarrassed to suggest using condoms to my partner (Okumu et al. 2022)*<br><br>*My husband and I decide together which form of birth control to use. (Ghiasi et al. 2022)*<br><br>*If I had a sexual partner, I would feel comfortable telling that person if I wanted to use a method to protect against infection or pregnancy, even when they did not want to. (Upadhyay et al. 2021)*<br><br>*If my partner would ask me to have unprotected sex I would refuse it. (Espada et al. 2013)*<br><br>*If I were going to have sex and realize that I have no condoms, I would wait until I have them to maintain sexual intercourse. (Espada et al. 2013)*<br><br>*I would be willing to publicly defend the use of condoms. (Espada et al. 2013)*<br><br>*If I try to do something, I can keep someone from getting hurt (Edwards et al. 2015)*<br><br>*Within next three months, I'm going to avoid risky behaviors (such as transfusions of infected blood, unprotected sex, etc.) that lead to HIV infection. (Darabi et al. 2018)*<br><br>*How would you feel talking about whether to use a condom? (Deardorff et al. 2008)*<br><br>*How would you feel putting a condom on a guy/having a girl put a condom on you? (Deardorff et al. 2008)*<br><br>*I would not know how to suggest to my partner to use condoms (Escribano et al. 2017)*<br><br>*Think about the first time you had sex … Did you or your partner use condoms that first time? (Yes, No, I've never had sex) (Jerman et al. 2015)*<br><br>*Convincing a partner to accept using a condom when having sex is easy. (Masa & Chowa 2014)*
	d) Health Promotion	Ability to make informed decisions on SH determinants in the social and physical environment	*If you had a boyfriend/girlfriend, how sure are you that you could refuse to have sex with him/her if you didn't feel ready? (L'Engle et al. 2006)*<br><br>*If I had sex with a guy, I would be running the risk of being played (taken advantage of)* (O'Sullivan et al. 2006)*<br><br>*When I decide to have sex with a guy, it will be because I wanted to have sex and not because he really wanted me to have sex with him (O'Sullivan et al. 2006)*<br><br>*If I kiss a guy I don't really know, I'm afraid of what he will try to make me do next (O'Sullivan et al. 2006)*<br><br>*I can refuse to have sex even if someone promises me a cell phone. (St. Lawrence et al. 2023)*<br><br>*I would have sex to get money to help my family.* (St. Lawrence et al. 2023)*<br><br>*Most ofthe time, we do what my partner wants to do. (Pulerwitz et al. 2018)*<br><br>*I can freely choose who I marry. (Upadhyay et al. 2022)*<br><br>*I do not feel afraid that I will be forced to do something sexually when I do not want to. (Upadhyay et al. 2021)*<br><br>*How would you feel talking about oral sex (going down)? (Deardorff et al. 2008)*<br><br>*Do you feel guilty about having sex?* (Deardorff et al. 2008)*<br><br>*How old were you when you had oral sex for the first time? (I've never had sex, 12 or younger, 13, 14, 15, 16, 17, 18 or older) (Jerman et al. 2015)*<br><br>*Did you ever talk with your current or most recent steady partner about your own sexual history? (Jerman et al. 2015)*

Dimension		Definition	Sample Item
**Context factors**	**e) Healthcare**	Context factors that can influence the ability to make informed decisions on medical issues	*My family will financially support me for costs associated with pregnancy (e.g. for purchasing foods, routine antenatal tests, ultrasound screening, or frequent hospitalizations), if needed. (Ghiasi et al. 2022)*<br><br>*Healthcare providers provide me all the information I need (pregnancy nutrition, sex in pregnancy, pregnancy risk symptoms, how to use contraception ...) without having to wait for a question from me. (Ghiasi et al. 2022)*<br><br>*Does the clinic need to get permission from a teen's parent or guardian in order to provide sexual health services? (Jerman et al. 2015)*
	**e) Disease Prevention**	Context factors that can influence the ability to make informed decisions on risk factors for SH	*My husband pays attention to his sexual hygiene. (Ghiasi et al. 2022)*<br><br>*If I asked my partner to use a condom, he would think I'm having sex with other people. (Pulerwitz et al. 2018)*<br><br>*I might get in trouble if I help (Edwards et al. 2015)*<br><br>*Staff would tell a group of boys calling a girl a "sluts" to stop (Edwards et al. 2015)*<br><br>*My sexual partner does not want to use them (Escribano et al. 2017)*<br><br>*Do teens have to pay for sexual health services (like birth control, pregnancy tests and STD tests)? (Jerman et al. 2015)*<br><br>*Adults in my life think condoms should be used during sex. (Masa & Chowa 2014)*
	**e) Health Promotion**	Context factors that can influence the ability to make informed decisions on SH determinants in the social and physical environment	*In my school, same-sex relationships are respected just as much as opposite-sex relationships. (Ullman et al. 2023)*<br><br>*I learned about bisexuality in my Health and Physical Education class at school. (Ullman et al. 2023)*<br><br>*My parents would not question me if they saw me with a man who was driving an expensive car.* (St. Lawrence et al. 2023)*<br><br>*I have a parent or guardian who accepts me as I am. (Upadhyay et al. 2021)*<br><br>*The media normalizes Sexual Assault (Edwards et al. 2015)*<br><br>*My family believes that I should continue my social activities during menstruation. (Darabi et al. 2018)*

*Reversed items
