# Supplementary material for: A COSMIN Systematic Review of Sexual Health Literacy Self-Report Measures for Adolescents
Source: Arch Sex Behav. 2025 Jun 6;54(5):1737–68. doi: 10.1007/s10508-025-03142-1 (PMC12162768; doi:10.1007/s10508-025-03142-1)
Supplement: Supplementary file 4 — Supplementary file4 (PDF 1029 KB) [file 10508_2025_3142_MOESM4_ESM.pdf]

Reasons for Exclusion of retrieved full-texts

Reason for Exclusion/ Inclusion	Author(s)	Year	Title	DOI
Construct of interest	Curwen, Tracey	2003	The Importance of Offense Characteristics, Victimization History, Hostility, and Social Desirability in Assessing Empathy of Male Adolescent Sex Offenders	10.1177/107906320301500410
Construct of interest	Hair, Elizabeth C.; Moore, Kristin Anderson; Garrett, Sarah B.; Kinukawa, Akemi; Lippman, Laura; Michelson, Erik	2006	The Parent-Adolescent Relationship Scale	10.1037/t16909-000.
Construct of interest	Bouchey, Heather A.	2007	Perceived romantic competence, importance of romantic domains, and psychosocial adjustment	10.1080/15374410701653120
Construct of interest	Goggin, K.; Malcarne, V. L.; Murray, T. S.; Metcalf, K. A.; Wallston, K. A.	2007	Do religious and control cognitions predict risky behavior? II. Development and validation of the Sexual Risk Behavior-related God Locus of Control Scale for Adolescents (SexGLOC-A)	10.1007/s10608-006-9090-1
Construct of interest	Funk, Jeanne; Fox, Christine; Chan, Margaret; Curtiss, Kathleen	2008	The development of the Children's Empathic Attitudes Questionnaire using classical and Rasch analyses	10.1016/j.appdev.2008.02.005
Construct of interest	Oneal, Brent J.; Burns, G. Leonard; Kahn, Timothy J.; Rich, Phil; Worling, James R.	2008	Initial psychometric properties of a treatment planning and progress inventory for adolescents who sexually abuse	10.1177/1079063208317465
Construct of interest	Runyon, M. K.; Steer, R. A.; Deblinger, E.	2009	Psychometric characteristics of the beck self-concept inventory for youth with adolescents who have experienced sexual abuse	10.1007/s10862-008-9100-6
Construct of interest	Levin-Zamir, Diane; Lemish, Dafna; Gofin, Rosa	2011	Media Health Literacy (MHL): Development and Measurement of the Concept among Adolescents	10.1093/her/cyr007
Construct of interest	Ortabag, Tulay; Ozdemir, Serpil; Bakir, Bilal; Tosun, Nuran	2011	Health promotion and risk behaviors among adolescents in Turkey	10.1177/1059840511408322
Construct of interest	Sesli, E.; Kara, Y.	2012	Development and application of a two-tier multiple-choice diagnostic test for high school students' understanding of cell division and reproduction	10.1080/00219266.2012.688849
Construct of interest	Ardelt, Peter U.; Cederqvist, Marco; Barth, Michael; Frankenschmidt, Alexander	2017	The SIGHT questionnaire: A novel assessment tool for Satisfaction In Genital Hypospadias Treatment	10.1016/j.jpurol.2016.01.016
Construct of interest	Choudhary, Vandana; Satapathy, Sujata; Sagar, Rajesh	2018	Development of a multi-dimensional scale to measure trauma associated with child sexual abuse (MSCSA) and its ramifying impacts on children: A pilot study	10.1016/j.ajp.2017.12.020
Construct of interest	García-Carpintero, María Ángeles; Rodríguez-Santero, Javier; Porcel-Gálvez, Ana María	2018	Design and validation of the scale for the detection of violence in courtship in young people in the Sevilla University (Spain)	10.1016/j.gaceta.2017.09.006
Construct of interest	Choudhary, Vandana; Satapathy, Sujata; Sagar, Rajesh	2019	Qualitative Study on the Impact of Child Sexual Abuse: Perspectives of Children, Caregivers, and Professionals in Indian Context	10.1080/10538712.2018.1563262
Construct of interest	Murshid, K. A. S.; Murshid, Nadine Shaanta	2019	Adolescent exposure to and attitudes toward violence: Empirical evidence from Bangladesh	10.1016/j.childyouth.2018.12.025
Construct of interest	Urrutia, C. I.; Baluch, B.; van den Akker, O.; Bailey-Rodriguez, D.	2019	Assisted reproductive technologies (ARTs) in the eyes of the mayans and ladinos	N/A
Construct of interest	Gerlsma, Coby; Lugtmeyer, Valerie; van Denderen, Mariëtte; Keijser, Jos de	2020	Revenge and forgiveness after victimization: Psychometric evaluation of a dutch version of the trim intended for victims and offenders	10.1007/s12144-020-01283-1
Construct of interest	Spitznagle, J.; Balmuri, N.; Adams, A.; Onel, K.; Taber, S.; Pan, N.	2020	Identifying Targets to Improve the Assessment of Psychosocial Risk Factors in Adolescent Patients: Perspectives from Pediatric Rheumatology Fellows in the United States and Canada	10.1002/art.41538
Construct of interest	van Ham, Kirsten; van Konijnenburg, Eva M. M. Hoytema; Brilleslijper-Kater, Sonja N.; Schepers, Amber; Daams, Joost G.; Teeuw, Arianne H.; van Rijn, Rick R.; van der Lee, Johanna H.	2020	A systematic review of instruments used to assess nonverbal emotional signs in children during an investigative interview for suspected sexual abuse	10.1002/car.2601
Construct of interest	Choudhary, Vandana; Satapathy, Sujata; Sagar, Rajesh	2021	Multidimensional scale for child sexual abuse (MSCSA): Development and psychometric properties	10.1016/j.ajp.2021.102643
Construct of interest	Devgun, Meetali; B N, Roopesh; Seshadri, Shekhar	2021	Breaking the silence: Development of a qualitative measure for inquiry of child sexual abuse (CSA) awareness and perceived barriers to CSA disclosure	10.1016/j.ajp.2021.102558
Construct of interest	Hinerman, Krystal M.; Hull, Darrell M.; Näslund-Hadley, Emma I.; Mirzaei Rafe, Mehri	2021	Social Emotional Learning Competencies in Belize Children: Psychometric Validation Through Exploratory Structural Equation Modeling	10.3389/fpsyg.2021.770501

Reason for Exclusion/ Inclusion	Author(s)	Year	Title	DOI
Construct of interest	Sopfe, Jenna; Marsh, Rebekah; Ziniel, Sonja I.; Klosky, James L.; Chow, Eric J.; Dorsey Holliman, Brooke; Peterson, Pamela N.	2021	Evaluation of the v2.0 Brief Profiles for Sexual Function and Satisfaction PROMIS in Adolescent and Young Adult Childhood Cancer Survivors	10.1089/jayao.2020.0166
Construct of interest	Zych, I.; Llorent, V. J.	2021	Bias-Based Cyberbullying in Spanish Adolescents and Its Relation to Social and Emotional Competencies and Technology Abuse	10.1177/02724316211020365
Construct of interest	Fekih-Romdhane, Feten; Dabbous, Mariam; Hallit, Rabih; Malaeb, Diana; Sawma, Toni; Obeid, Sahar; Hallit, Souheil	2022	Development and validation of a shortened version of the Child Abuse Self Report Scale (CASRS-12) in the Arabic language	10.1186/s13034-022-00533-3
Construct of interest	Leite, Paloma Loiola; Torres, Francisco Ayslan Ferreira; Pereira, Leonarda Marques; Bezerra, Adriana de Moraes; Machado, Lucas Dias Soares; Silva, Maria Rocineide Ferreira da	2022	Construction and validation of podcast for teen sexual and reproductive health education	10.1590/1518-8345.6263.3706
Inclusion	Reininger, B.; Evans, A. E.; Griffin, S. F.; Valois, R. F.; Vincen, M. L.; Parra-Medina, D.; Taylor, D. J.; Zullig, K. J.	2003	Development of a youth survey to measure risk behaviors, attitudes and assets: examining multiple influences	10.1093/her/cyf046
Inclusion	Friedrich, William N.; Lysne, Marit; Sim, Leslie; Shamos, Susan	2004	Assessing Sexual Behavior in High-Risk Adolescents with the Adolescent Clinical Sexual Behavior Inventory (ACSBI)	10.1177/1077559504266907
Inclusion	Chu, Judy Y.; Porche, Michelle V.; Tolman, Deborah L.	2005	The Adolescent Masculinity Ideology in Relationships Scale	10.1177/1097184X03257453
Inclusion	Grover, Rachel L.; Nangle, Douglas W.; Zeff, Karen R.	2005	The Measure of Adolescent Heterosocial Competence: Development and Initial Validation	10.1207/s15374424jccp3402_7
Inclusion	Stevens-Simon, Catherine; Sheeder, Jeanelle; Beach, Roberta; Harter, Susan	2005	Adolescent pregnancy: Do expectations affect intentions?	10.1016/j.jadohealth.2005.01.007
Inclusion	Hannawa, A. F.; Spitzberg, B. H.; Wiering, L.; Teranishi, C.	2006	"If I can't have you, no one can": Development of a Relational Entitlement and Proprietariness Scale (REPS)	10.1891/0886-6708.21.5.539
Inclusion	L'Engle, Kelly Ladin; Jackson, Christine; Brown, Jane D.	2006	Early Adolescents' Cognitive Susceptibility To Initiating Sexual Intercourse	10.1111/j.1931-2393.2006.tb00066.x
Inclusion	O'Sullivan, Lucia F.; Meyer-Bahlburg, Heino F. L.; McKeague, Ian W.	2006	The Development of the Sexual Self-Concept Inventory for Early Adolescent Girls	10.1111/j.1471-6402.2006.00277.x
Inclusion	Grover, Rachel L.; Nangle, Douglas W.; Serwik, Agnieszka; Zeff, Karen R.	2007	Girl friend, boy friend, girlfriend, boyfriend: broadening our understanding of heterosocial competence	10.1080/15374410701651637
Inclusion	Hutchinson, M. Katherine; Jemmott, Loretta Sweet; Wood, Elyssa B.; Hewitt, Herme; Kahwa, Eulalia; Waldron, Norman; Bonaparte, Beverly	2007	Culture-specific factors contributing to HIV risk among Jamaican adolescents	10.1016/j.jana.2007.01.008
Inclusion	Roye, Carol; Perlmutter Silverman, Paula; Krauss, Beatrice	2007	A brief, low-cost, theory-based intervention to promote dual method use by black and Latina female adolescents: a randomized clinical trial	10.1177/1090198105284840
Inclusion	Volpe, E. M.; Nelson, L. E.; Kraus, R. A.; Morrison-Beedy, D.	2007	Adaptation and Refinement of the HIV Knowledge Questionnaire for Use With Adolescent Girls	10.1016/j.jana.2007.07.003
Inclusion	Deardorff, Julianna; Tschann, Jeanne M.; Flores, Elena	2008	Sexual values among Latino youth: Measurement development using a culturally based approach	10.1037/1099-9809.14.2.138
Inclusion	Sales, Jessica McDermott; Milhausen, Robin R.; Wingood, Gina M.; DiClemente, Ralph J.; Salazar, Laura F.; Crosby, Richard A.	2008	Validation of a Parent-Adolescent Communication Scale for use in STD/HIV prevention interventions	10.1177/1090198106293524
Inclusion	Skinner, S. Rachel; Smith, Jennifer; Fenwick, Jennifer; Fyfe, Sue; Hendriks, Jacqueline	2008	Perceptions and experiences of first sexual intercourse in Australian adolescent females	10.1016/j.jadohealth.2008.04.017
Inclusion	Mũkoma, Wanjirũ; Flisher, Alan J.; Helleve, Arnfinn; Aarø, Leif Edvard; Mathews, Catherine; Kaaya, Sylvia; Klepp, Knut-Inge	2009	Development and test-retest reliability of a research instrument designed to evaluate school-based HIV/AIDS interventions in South Africa and Tanzania	10.1177/1403494809103995
Inclusion	Sales, Jessica M.; Spitalnick, Josh; Milhausen, Robin R.; Wingood, Gina M.; DiClemente, Ralph J.; Salazar, Laura F.; Crosby, Richard A.	2009	Validation of the worry about sexual outcomes scale for use in STI/HIV prevention interventions for adolescent females	10.1093/her/cyn006
Inclusion	Skinner, S. Rachel; Smith, Jennifer; Fenwick, Jennifer; Hendriks, Jacqueline; Fyfe, Sue; Kendall, Garth	2009	Pregnancy and protection: perceptions, attitudes and experiences of Australian female adolescents	10.1016/j.wombi.2008.12.001

Reason for Exclusion/ Inclusion	Author(s)	Year	Title	DOI
Inclusion	Wherry, Jeffrey N.; Berres, Ashley K.; Sim, Leslie; Friedrich, William N.	2009	Factor structure of the Adolescent Clinical Sexual Behavior Inventory	10.1080/10538710902881428
Inclusion	Saydam, B. K.; Ceber, E.; Bilge, A.; Can, H. O.; Mermer, G.; Demireloz, M.; Ozenturk, G.	2010	Reliability and Validity of the Reproductive Health Scale for Turkish Adolescents	10.5336/medsci.2008-9459
Inclusion	Aarø, Leif E.; Breivik, Kyrre; Klepp, Knut-Inge; Kaaya, Sylvia; Onya, Hans E.; Wubs, Annegreet; Helleve, Arnfinn; Flisher, Alan J.	2011	An HIV/AIDS knowledge scale for adolescents: Item response theory analyses based on data from a study in South Africa and Tanzania	10.1093/her/cyq086
Inclusion	Bourdeau, Beth; Grube, Joel W.; Bersamin, Melina M.; Fisher, Deborah A.	2011	The role of beliefs in sexual behavior of adolescents: Development and validation of an Adolescent Sexual Expectancies Scale (ASEXS)	10.1111/j.1532-7795.2010.00697.x
Inclusion	Buhi, Eric R.; Goodson, Patricia; Neilands, Torsten B.; Blunt, Heather	2011	Adolescent sexual abstinence: a test of an integrative theoretical framework	10.1177/1090198110375036
Inclusion	Patrick, Megan E.; Maggs, Jennifer L.; Cooper, M. Lynne; Lee, Christine M.	2011	Measurement of motivations for and against sexual behavior	10.1177/1073191110372298
Inclusion	Tobin, Casey T.	2011	Development of the Sexual Attitudes and Experiences Scale (SAES)	10.1037/t06548-000
Inclusion	Vyncke, Johanna D.; Julien, Danielle; Jodoin, Emilie; Jouvin, Emilie	2011	Development and initial validation of the Perceived Heterosexism Scale and the Preoccupation with Disclosure of Parents' Sexual Orientation Scale	10.1080/1550428X.2011.592963
Inclusion	Hendriks, Jacqueline; Fyfe, Sue; Styles, Irene; Skinner, S. Rachel; Merriman, Gareth	2012	Scale construction utilising the Rasch unidimensional measurement model: A measurement of adolescent attitudes towards abortion	10.4066/AMJ.2012.952
Inclusion	Hutchinson, M. K.; Smith, T. K.; Waldron, N.; Kahwa, E.; Hewitt, H. H.; Hamilton, P. I.; Kang, S-Y	2012	Validation of the Jamaican Maternal Sexual Role Modelling Questionnaire	10.7727/wimj.2011.198
Inclusion	Hutchinson, M. Katherine; Kahwa, Eulalia; Waldron, Norman; Hepburn Brown, Cerese; Hamilton, Pansy I.; Hewitt, Hermi H.; Aiken, Joyette; Cederbaum, Julie; Alter, Emily; Sweet Jemmott, Loretta	2012	Jamaican mothers' influences of adolescent girls' sexual beliefs and behaviors	10.1111/j.1547-5069.2011.01431.x
Inclusion	Lusczakoski, Kathryn D.; Rue, Lisa A.	2012	The OPTIONS model of sexual risk assessment for adolescents	10.1177/1524839910385898
Inclusion	Prati, Gabriele	2012	Development and psychometric properties of the Homophobic Bullying Scale	10.1177/0013164412440169
Inclusion	Rye, B. J.; Meaney, Glenn J.; Yessis, Jennifer; McKay, Alexander	2012	Uses of the 'Comfort with Sexual Matters for Young Adolescents' scale: A measure of erotophobia-erotophilia for youth	N/A
Inclusion	Ward, Rose Marie; Matthews, Molly R.; Weiner, Judith; Hogan, Kathryn M.; Popson, Halle C.	2012	Alcohol and sexual consent scale: development and validation	10.5993/AJHB.36.6.3
Inclusion	Zimmer-Gembeck, Melanie J.; Hughes, Nicole; Kelly, Marguerite; Connolly, Jennifer	2012	Intimacy, identity and status: Measuring dating goals in late adolescence and emerging adulthood	10.1007/s11031-011-9253-6
Inclusion	Espada, Jose P.; Ballester, Rafael; Huedo-Medina, Tania; Secades-Villa, Roberto; Orgilés, Mireia; Martínez-Lorca, Manuela	2013	Desarrollo de un nuevo instrumento para evaluar las actitudes hacia el VIH/SIDA en adolescentes españoles	10.6018/analesps.29.1.132301
Inclusion	Wang, Ruey-Hsia; Jian, Shu-Yuan; Yang, Yung-Mei	2013	Psychometric testing of the Chinese version of the Contraceptive Behavior Scale: A preliminary study	10.1111/j.1365-2702.2011.03785.x
Inclusion	Yonas, Michael A.; Burke, Jessica G.; Miller, Elizabeth	2013	Visual voices: a participatory method for engaging adolescents in research and knowledge transfer	10.1111/cts.12028
Inclusion	Masa, Rainier D.; Chowa, Gina A.	2014	HIV risk among young Ghanaians in high school: validation of a multidimensional attitude towards condom use scale	10.1080/02673843.2014.963629
Inclusion	Nelas, Paula; Ferreira, Manuela; Fernandes, Carlos; Duarte, João; Chaves, Cláudia	2014	Scale of knowledge about sexually transmitted infections	10.1016/S0212-6567(14)70092-7
Inclusion	Nickerson, Amanda B.; Aloe, Ariel M.; Livingston, Jennifer A.; Feeley, Thomas Hugh	2014	Measurement of the bystander intervention model for bullying and sexual harassment	10.1016/j.adolescence.2014.03.003
Inclusion	Ullman, Jacqueline	2014	Ladylike/butch, sporty/dapper: exploring 'gender climate' with Australian LGBTQ students using stage–environment fit theory	10.1080/14681811.2014.919912
Inclusion	Edwards, Katie M.; Rodenhizer-Stämpfli, Kara Anne; Eckstein, Robert P.	2015	Bystander Action in Situations of Dating and Sexual Aggression: A Mixed Methodological Study of High School Youth	10.1007/s10964-015-0307-z

Reason for Exclusion/ Inclusion	Author(s)	Year	Title	DOI
Inclusion	Guttersrud, Øystein; Naigaga, Mpolampola Desire Alice Sandra; Pettersen, Kjell Sverre	2015	Measuring Maternal Health Literacy in Adolescents Attending Antenatal Care in Uganda: Exploring the Dimensionality of the Health Literacy Concept Studying a Composite Scale	10.1891/1061-3749.23.2.E50
Inclusion	Mushwana, L.; Monareng, L.; Richter, S.; Muller, H.	2015	Factors influencing the adolescent pregnancy rate in the Greater Giyani Municipality, Limpopo Province - South Africa	10.1016/j.ijans.2015.01.001
Inclusion	Sánchez, Virginia; Muñoz-Fernández, Noelia; Ortega-Ruíz, Rosario	2015	"Cyberdating Q_A": An instrument to assess the quality of adolescent dating relationships in social networks	10.1016/j.chb.2015.01.006
Inclusion	Acharya, Dev Raj; Thomas, Malcolm; Cann, Rosemary	2016	Validation of a questionnaire to measure sexual health knowledge and understanding (Sexual Health Questionnaire) in Nepalese secondary school: A psychometric process	10.4103/2277-9531.184560
Inclusion	Bianchi, Dora; Morelli, Mara; Baiocco, Roberto; Chirumbolo, Antonio	2016	Psychometric properties of the Sexting Motivations Questionnaire for adolescents and young adults	10.4558/8067-01
Inclusion	Biney, Adriana Andrea Ewurabena	2016	A different approach in developing a sexual self-concept scale for adolescents in Accra, Ghana	10.1007/s12119-015-9331-0
Inclusion	Jerman, P.; Berglas, N. F.; Rohrbach, L. A.; Constantine, N. A.	2016	Test-retest reliability of self-reported sexual health measures among US Hispanic adolescents	10.1177/0017896915589420
Inclusion	Berglas, Nancy F.; Constantine, Norman A.; Jerman, Petra; Rohrbach, Louise A.	2017	Development and assessment of measures of adolescents' attitudes about sexual relationship rights	10.1080/19317611.2016.1256364
Inclusion	Emmerink, Peggy M. J.; van den Eijnden, Regina J J M; Ter Bogt, Tom F M; Vanwesenbeeck, Ine	2017	A Scale for the Assessment of Sexual Standards Among Youth: Psychometric Properties	10.1007/s10508-017-1001-x
Inclusion	Escribano, Silvia; Espada, José P.; Morales, Alexandra; Orgilés, Mireia	2017	Psychometric Properties of the Condom Use Barriers Scale for Adolescents	10.1017/sjp.2017.64
Inclusion	Sim-Sim, Maria Margarida; Viana, Elsa	2017	TRADUÇÃO E VALIDAÇÃO DA ESCALA ATTITUDES TOWARD SEX EDUCATION (ATSES) EM ADOLESCENTES PORTUGUESES<br>TRANSLATION AND VALIDATION OF ATTITUDES TOWARD SEX EDUCATION (ATSES) AT PORTUGUESE ADOLESCENTS	10.24902/r.riase.2016.2(2).559
Inclusion	Smith, Haylie; Perez, Marisol; Sladek, Michael R.; Becker, Carolyn Black; Ohrt, Tara K.; Bruening, Amanda B.	2017	Development and validation of makeup and sexualized clothing questionnaires	10.1186/s40337-017-0171-1
Inclusion	Darabi, Fatemeh; Yaseri, Mehdi; Safari, Hossein; Kaveh, Mohammad Hossein; Khalaj Abadi Farahani, Farideh; Shojaeizadeh, Davoud	2018	Developmental and Psychometric Properties of a Belief-based Reproductive Health Behavior Questionnaire for Female Adolescents	N/A
Inclusion	Edwards, Katie M.; Banyard, Victoria L.; Sessarego, Stephanie N.; Stanley, Linda R.; Mitchell, Kimberly J.; Eckstein, Robert P.; Rodenhizer, Kara Anne E.; Leyva, P. Caroline	2018	Measurement Tools to Assess Relationship Abuse and Sexual Assault Prevention Program Effectiveness Among Youth	10.1037/vio0000151
Inclusion	Fefferman, Ann M.; Upadhyay, Ushma D.	2018	Hybrid Masculinity and Young Men's Circumscribed Engagement in Contraceptive Management	10.1177/0891243218763313
Inclusion	Fisher, Celia B.; Fried, Adam L.; Desmond, Margaret; Macapagal, Kathryn; Mustanski, Brian	2018	Perceived Barriers to HIV Prevention Services for Transgender Youth	10.1089/lgbt.2017.0098
Inclusion	Garrido-Hernansaiz, Helena; Martín-Fernández, Manuel; Castaño-Torrijos, Aida; Cuevas, Isabel	2018	Development and Validation of the ADAS Scale and Prediction of Attitudes Toward Affective-Sexual Diversity Among Spanish Secondary Students	10.1080/00918369.2017.1364951
Inclusion	Pulerwitz, Julie; Mathur, Sanyukta; Woznica, Daniel	2018	How empowered are girls/young women in their sexual relationships? Relationship power, HIV risk, and partner violence in Kenya	10.1371/journal.pone.0199733
Inclusion	Apidechkul, T.	2019	Sexual behaviors and seroprevalence of HIV, HBV, and HCV among hill tribe youths of Northern Thailand	10.1186/s12889-019-7459-9
Inclusion	Guerra, Cristina; Del Río, Francisco Javier; Cabello, Francisco; Morales, Isabel María	2019	Creation and validation of a scale of sexuality for adolescents: Scale of Myths about Sexuality	10.1016/j.androl.2018.06.001
Inclusion	Morales, Alexandra; Espada, José P.; Reis, Sibília; Orgilés, Mireia	2019	Portuguese version of the HIV-related Attitudes Scale (HIV-AS) for adolescents: Adaptation, validation, and equivalency factor with the Spanish version	10.1027/1015-5759/a000426
Inclusion	Vongxay, Viengnakhone; Albers, Femke; Thongmixay, Souksamone; Thongsombath, Maliphone; Broerse,	2019	Sexual and reproductive health literacy of school adolescents in Lao PDR	10.1371/journal.pone.0209675

Reason for Exclusion/ Inclusion	Author(s)	Year	Title	DOI
	Jacqueline E. W.; Sychareun, Vanphanom; Essink, Dirk Rombout			
Inclusion	Barros, Teresa Madalena Kraus Brincheiro Hüttel; Ramalho, Sónia Isabel Horta Salvo Moreira de Almeida; Gordo, Clementina Maria Gomes de Oliveira; Frade, João Manuel Graça; Luz, Alexandra; Moleiro, Pascoal; Dixe, Maria Dos Anjos Coelho Rodrigues	2020	ADOLESCENT STUDENTS' ATTITUDES TOWARDS SEXUALITY: THE CONSTRUCTION AND VALIDATION OF A SCALE	10.1590/1984-0462/2021/39/2019372
Inclusion	Gómez-Lugo, Mayra; Morales, Alexandra; Saavedra-Roa, Alejandro; Niebles-Charris, Janivys; García-Roncallo, Paola; Marchal-Bertrand, Laurent; Espada, José Pedro; Vallejo-Medina, Pablo	2020	Psychometric Properties of the Colombian Version of the HIV Attitudes Scale for Adolescents	10.3390/ijerph17134686
Inclusion	Kyegombe, Nambusi; Meiksin, Rebecca; Wamoyi, Joyce; Heise, Lori; Stoebenau, Kirsten; Buller, Ana Maria	2020	Sexual health of adolescent girls and young women in Central Uganda: exploring perceived coercive aspects of transactional sex	10.1080/26410397.2019.1700770
Inclusion	Upadhyay, Ushma D.; Lipkovich, Heather	2020	Using online technologies to improve diversity and inclusion in cognitive interviews with young people	10.1186/s12874-020-01024-9
Inclusion	Yau, Shamsudeen; Wongsawat, Pramote; Songthap, Archin	2020	Knowledge, Attitude and Perception of Risk and Preventive Behaviors toward Premarital Sexual Practice among In-School Adolescents	10.3390/ejihpe10010036
Inclusion	Zakaria, M.; Karim, F.; Mazumder, S.; Cheng, F.; Xu, J.	2020	Knowledge on, attitude towards, and practice of sexual and reproductive health among older adolescent girls in Bangladesh: An institution-based cross-sectional study	10.3390/ijerph17217720
Inclusion	Abello-Luque, Daniella; Espada, José Pedro; García-Montaño, Eileen; Gómez-Lugo, Mayra; Morales, Alexandra; Pérez-Pedraza, Diana; Vallejo-Medina, Pablo	2021	Colombian Adaptation of the HIV and Other Sexually Transmitted Infections Knowledge Scale (KSI) in an Adolescent Population	10.1177/0163278720979621
Inclusion	Hill, Amber L.; Miller, Elizabeth; Switzer, Galen E.; Abebe, Kaleab Z.; Chang, Judy C.; Pulerwitz, Julie; Brush, Lisa D.; Hill, Ashley V.	2021	Gender equitable attitudes among adolescents: A validation study and associations with sexual health behaviors	10.1007/s40894-021-00171-4
Inclusion	Ma, Xuemei; Yang, Yufan; Wei, Qian; Jiang, Hong; Shi, Huijing	2021	Development and validation of the reproductive health literacy questionnaire for Chinese unmarried youth	10.1186/s12978-021-01278-6
Inclusion	Upadhyay, Ushma D.; Danza, Phoebe Y.; Neilands, Torsten B.; Gipson, Jessica D.; Brindis, Claire D.; Hindin, Michelle J.; Foster, Diana Greene; Dworkin, Shari L.	2021	Development and Validation of the Sexual and Reproductive Empowerment Scale for Adolescents and Young Adults	10.1016/j.jadohealth.2020.05.031
Inclusion	Yusufov, Miryam; Orchowski, Lindsay M.	2021	Readiness to engage in assertive responding, self-protective dating behaviors, and sexual communication: A transtheoretical model-based analysis of college women	10.1080/07448481.2020.1719111
Inclusion	Ghiasi, Ashraf; Keramat, Afsaneh; Zayeri, Farid; Farjamfar, Maryam; Vakilian, Katayon; Bagheri, Leila	2022	Design and psychometric properties of a questionnaire for assessing sexual and reproductive health needs of married adolescent women: an exploratory sequential mixed methods study	10.1080/01443615.2022.2109132
Inclusion	Kutner, Bryan A.; Perry, Nicholas S.; Stout, Claire; Norcini Pala, Andrea; Paredes, Christian D.; Nelson, Kimberly M.	2022	The Inventory of Anal Sex Knowledge (iASK): A New Measure of Sexual Health Knowledge Among Adolescent Sexual Minority Males	10.1016/j.jsxm.2021.12.011
Inclusion	Lubis, R.; Hinduan, Z. R.; Jatnika, R.; Baydhowi, B.; Agustiani, H.	2022	The Development and Initial Validation of the Youth Sexual Intention Scale: Indonesian Version	10.1177/00469580221087833
Inclusion	Okumu, Moses; Logie, Carmen H.; Ansong, David; Mwima, Simon; Hakiza, Robert; Newman, Peter A.	2022	Support for Texting-Based Condom Negotiation Among Forcibly Displaced Adolescents in the Slums of Kampala, Uganda: Cross-sectional Validation of the Condom Use Negotiated Experiences Through Technology Scale	10.2196/27792
Inclusion	Ren, Zhengjia; Liu, Yanhong; Deng, Jianjun	2022	Development and Validation of the Chinese Version of The Masturbation Beliefs Scale	10.1016/j.esxm.2022.100501
Inclusion	Stoebenau, Kirsten; Bingenheimer, Jeffrey Bart; Kyegombe, Nambusi; Datar, Reva; Ddumba-Nyanzi, Ismael	2022	Development of the Gender Roles and Male Provision Expectations Scale	10.1007/s10508-022-02479-1

Reason for Exclusion/ Inclusion	Author(s)	Year	Title	DOI
Inclusion	St Lawrence, Janet S.; Sun, Christina J.; Seloilwe, Esther S.; Magowe, Mabel K. M.; Rampa, Shathani; Dithole, Kefalotse S.	2023	Batswana Adolescents' Attitudes Toward Sex With Older Adults: Psychometric Properties of the Attitudes Toward Transactional Sex Scale	10.1177/10731911221146518
Inclusion	Ullman, Jacqueline; Hobby, Lucy; Magson, Natasha R.; Zhong, Hua Flora	2023	Students' perceptions of the rules and restrictions of gender at school: A psychometric evaluation of the Gender Climate Scale (GCS)	10.3389/fpsyg.2023.1095255
Language (Other)	Chang, Soon-Bok; Lee, Mi-Kyeong	2003	Sexual autonomy in college students	10.4040/jkan.2003.33.3.339
Language (Other)	Fernández-Fuertes, Andrés A.; Fuertes, Antonio; Pulido, Ramón F.	2006	Evaluación de la violencia en las relaciones de pareja de los adolescentes Validación del Conflict in Adolescent Dating Relationships Inventory (CADRI) - Versión española = Assessment of violence in adolescent couples Validation of the Conflict in Adolescent Dating Relationships Inventory (CADRI) - Spanish version	10.1037/t00856-000.
Language (Other)	Vega, Verónica Corina	2006	Construccion de un instrumento para la medicion de la conducta sexual en adolescentes femeninas: El CCS = Assessment of a sexual behavior questionnaire for female adolescents: The CCS	10.1037/t19271-000.
Language (Other)	Magalhães, Eunice; Oliveira, Grace K.; Leitão, Filomena; Chaves, Séli; Capela, Susana; Nogueira, Conceição; Martins, Carla	2007	Adaptação do 'Inventário de Sexismo Ambivalente' para uma população de estudantes universitários Portugueses = Adaptation of the Ambivalent Sexism Inventory for a population of a Portuguese students	10.1037/t00700-000.
Language (Other)	Teva, Inmaculada; Bermúdez, María Paz	2008	Adaptación castellana y propiedades psicométricas de la escala de búsqueda de sensaciones sexuales en adolescentes Españoles = Psychometric properties of a Spanish adaptation of the Sexual Sensation Seeking Scale in Spanish adolescents	10.1037/t04803-000
Language (Other)	Sierra, J. C.; Perla, F.; Gutierrez-Quintanilla, R.	2010	Attitudes Toward Masturbation in Adolescents: Psychometric Properties of Spanish Version of Attitudes Toward Masturbation Inventory	N/A
Language (Other)	Vargas, Elvia; Ponsoda, Vicente	2010	Escala de actividad sexual: Un instrumento para predecir el inicio temprano de relaciones sexuales = Sexual Activity Scale: A measure to predict the early onset of sexual intercourse	N/A
Language (Other)	Karaçam, Özgür; Totan, Tarık; Korkmaz, Yeşim Babür; Koyuncu, Mehmet	2012	Hendrick Cinsel Tutum Ölçeği Kısa Formunun Türkçeye uyarlanması, geçerlilik ve güvenilirlik çalışması = Turkish adaptation of the Hendrick Brief Sexual Attitudes Scale, validity and reliability study	N/A
Language (Other)	Martins, Sónia; Machado, Carla; Abrunhosa, Rui; Manita, Celina	2012	Escala de crenças sobre violência sexual (ECVS) = Scale of beliefs about sexual violence (ECVS)	N/A
Language (Other)	Lima-Serrano, M.; Lima-Rodríguez, J. S.; Sáez-Bueno, A.; Cáceres-Rodríguez, B.	2013	Design and validation of scales to measure adolescent attitudes toward sexuality, addictive substances and road safety. Are they related to behaviour?	10.4321/s1137-66272013000200004
Language (Other)	Sierra, J. C.; Iglesias, P. S.; Monge, F. S.	2013	Negative Attitudes Toward Masturbation Inventory: Validity, reliability, and proposal of a reduced version for adolescents	N/A
Language (Other)	Sierra, Juan Carlos; Pablo Santos Iglesias; Monge, Fredy S.	2013	NEGATIVE ATTITUDES TOWARD MASTURBATION INVENTORY: VALIDITY, RELIABILITY, AND PROPOSAL OF A REDUCED VERSION FOR ADOLESCENTS / INVENTARIO DE ACTITUDES NEGATIVAS HACIA LA MASTURBACIÓN: VALIDEZ, FIABILIDAD Y PROPUESTA DE UNA VERSIÓN REDUCIDA PARA POBLACIÓN ADOLESCENTE	N/A
Language (Other)	Yago Simón, Teresa; Tomás Aznar, Concepción	2013	Gender-determinant factors in contraception: design and validation of a questionnaire	10.1016/j.aprim.2013.04.013
Language (Other)	Bello-Villanueva, Ana Mercedes; Oviedo-Trespalac, Oscar; Vera-Villarroel, Pablo; Oviedo, Óscar; Rodriguez-Diaz, Melissa; Celis-Atenas, Karem; Pavez, Paula	2014	Presentación de una escala para evaluar actitudes y creencias sobre la sexualidad reproductiva en adolescentes varones de la región Caribe colombiana = Presentation of an Attitudes and Beliefs Scale to assess adolescent sexuality and reproductive health of young males in the Caribbean region	10.11144/Javeriana.UPSY13-1.peea
Language (Other)	Espada, José Pedro; Guillén-Riquelme, Alejandro; Morales, Alexandra; Orgilés, Mireia; Sierra, Juan Carlos	2014	Validation of an HIV and other sexually transmitted infections knowledge scale in an adolescent population	10.1016/j.aprim.2014.03.007
Language (Other)	Pápay, Nikolett; Rigó, Adrien; Nagybányai Nagy, Olivér; Soltész, Adrienn	2014	A gyermekvállalási attitűdök alakulásának pszichoszociális meghatározói = Psychosocial factors influencing the motivation for parenthood	10.1556/Mental.15.2014.1.1
Language (Other)	Bello-Villanueva, Ana Mercedes; Palacio, Jorge; Vera-Villarroel, Pablo; Oviedo-Trespalacios, Oscar;	2016	Construcción y validación de una escala para evaluar salud sexual y reproductiva en adolescentes mujeres de la Región Caribe Colombiana = The development and	10.1037/t61799-000.

Reason for Exclusion/ Inclusion	Author(s)	Year	Title	DOI
	Rodríguez-Díaz, Melissa Alejandra; Celis-Atenas, Karem; Pavez, Paula		validation of a scale to measure reproductive and sexual health of young women in the Colombian Caribbean region	
Language (Other)	Guerra, C.; Del Rio, F. J.; Im Morales; Cabello, F.	2017	Validation of the reduced version for adolescents of the Revised sexual opinion survey	10.1016/j.androl.2016.10.006
Language (Other)	Vallejo-Medina, P.; Saavedra-Roa, A.; Gomez-Lugo, M.; Morales, A.; Abello-Luque, D.; Garcia-Montano, E.; Garavito-Zamudio, C.; Espada, J. P.	2018	Adaptation, reliability and validity of a Brief Multicomponent AIDS Phobia Scale (MAPS) in Colombian adolescents	10.23923/j.rips.2018.01.014
Population	Bramwell, R. S.; Biswas, E. L.; Anderson, C.	2002	Using the Menstrual Attitude Qestionnaire with a British and an Indian sample	10.1080/026468302760270818
Population	Gregson, S.; Zhuwau, T.; Ndlovu, J.; Nyamukapa, C. A.	2002	Methods to reduce social desirability bias in sex surveys in low-development settings: experience in Zimbabwe	N/A
Population	Khandaker, R. I.; Vereecken, R. L.; Nijs, P.	2002	Psychosexual impacts of contraception on partner relationship	10.1080/14681990220108009
Population	Scandell, D. J.; Wlazelek, B.	2002	A validation study of the AIDS Health Belief Scale	N/A
Population	Abdala, N.; Carney, J. M.; Durante, A. J.; Klimov, N.; Ostrovski, D.; Somlai, A. M.; Kozlov, A.; Heimer, R.	2003	Estimating the prevalence of syringe-borne and sexually transmitted diseases among injection drug users in St Petersburg, Russia	10.1258/095646203322387965
Population	Abel, Elizabeth; Tak, Sung Hee; Gortner, Eva-Maria	2003	Reliability and Validity of Motivation for Sexual Health	10.1177/0193945903252423
Population	Dadds, M. R.; Smallbone, S.; Nisbet, I.; Dombrowski, J.	2003	Willingness, confidence, and knowledge to work with adolescent sex offenders: An evaluation of training workshops	10.1375/bech.20.2.117.24839
Population	Papagrigoriadis, S.; Heyman, B.	2003	Patients' views on follow up of colorectal cancer: Implications for risk communication and decision making	10.1136/pmj.79.933.403
Population	Russell, Glenda M.; Richards, Jeffrey A.	2003	Stressor and resilience factors for lesbians, gay men, and bisexuals confronting antigay politics	10.1023/A:1023919022811
Population	Yancey, Elleen M.; Wang, Min Qi; Goodin, Lisa; Cockrell, Tarisha	2003	HIV/AIDS knowledge scale in relation to HIV risks among African-American women	10.2466/PR0.92.3.991-996
Population	Abell, Neil; Nalavany, Blace A.	2004	An initial validation of a measure of personal and social perceptions of the sexual abuse of males	10.1177/1049731504265836
Population	Guerra, Valeschka Martins; Andrade, Fernando Cezar B. de; Dias, Mardonio Rique	2004	Atitudes de estudantes universitários frente ao consume de materiais pornográficos = University students' attitudes towards pornographic material consumption	10.1590/S1413-294X2004000200008
Population	Hernández, Gabriela Saldívar; Lira, Luciana Ramos; Méndez, María Teresa Saltijeral	2004	Validación de las escalas de aceptación de la violencia y de los mitos de violación en estudiantes universitarios = Validation of the violence acceptance scale and rape myths in university students	10.1037/t01100-000.
Population	Moe, B. K.; King, A. R.; Bailly, M. D.	2004	Retrospective accounts of recurrent parental physical abuse as a predictor of adult laboratory-induced aggression	10.1002/ab.20019
Population	Mongeau, Paul A.; Serewicz, Mary Claire Morr; Therrien, Lona Ficara	2004	Goals for Cross-Sex First Dates: Identification, Measurement, and the Influence of Contextual Factors	10.1080/0363775042331302514
Population	Nalavany, Blace A.; Abell, Neil	2004	An Initial Validation of a Measure of Personal and Social Perceptions of the Sexual Abuse of Males	10.1177/1049731504265836
Population	Speizer, Ilene S.; Santelli, John S.; Afable-Munsuz, Aimee; Kendall, Carl	2004	Measuring Factors Underlying Intendedness Of Women's First and Later Pregnancies	10.1111/j.1931-2393.2004.tb00023.x
Population	Cecil, Heather; Pinkerton, Steven D.; Bogart, Laura M.; Pavlovic, Jelena; Kimball, Allison M.	2005	An empirical study of ordinal condom use measures	10.1080/00224490509552291
Population	Ellis, Lee; Robb, Brian; Burke, Donald	2005	Sexual Orientation in United States and Canadian College Students	10.1007/s10508-005-6283-8
Population	Krook, Katja; Sandnabba, Kenneth N.; Santtila, Pekka; Wannas, Malin	2005	Multivariate structure of sexual behaviors in children: associations with age, social competence, life stressors, and behavioral disorders	10.1080/0300443042000206246
Population	LaBrie, Joseph W.; Quinlan, Thomas; Schiffman, Jason E.; Earleywine, Mitchell E.	2005	Performance of Alcohol and Safer Sex Change Rulers Compared With Readiness to Change Questionnaires	10.1037/0893-164X.19.1.112
Population	Lin, Peter; Simoni, Jane M.; Zemon, Vance	2005	The health belief model, sexual behaviors, and HIV risk among Taiwanese immigrants	10.1521/aeap.2005.17.5.469

Reason for Exclusion/ Inclusion	Author(s)	Year	Title	DOI
Population	Mathes, Eugene W.	2005	Men's Desire For Children Carrying Their Genes and Sexual Jealousy: A Test of Paternity Uncertainty as an Explanation of Male Sexual Jealousy	10.2466/PR0.96.3.791-798
Population	Morrison, Todd G.; Kenny, Paula; Harrington, Aoife	2005	Modern prejudice toward gay men and lesbian women: assessing the viability of a measure of modern homonegative attitudes within an Irish context	10.3200/MONO.131.3.219-250
Population	Nemčić, N.; Novak, S.; Marić, L.; Novosel, I.; Kronja, O.; Hren, D.; Marušić, A.; Marušić, M.	2005	Development and validation of questionnaire measuring attitudes towards sexual health among university students	N/A
Population	Qadir, F.; Silva, P. de; Prince, M.; Khan, M.	2005	Marital satisfaction in Pakistan: A pilot investigation	10.1080/14681990500113260
Population	Sherer, Moshe; Etgar, Talia	2005	Attitudes toward sex and sex offences among Israeli and former Union of Soviet Socialist Republic youth: implication for prevention strategies for new immigrants	10.1177/0886260504271478
Population	Sills, Terrence; Wunderlich, Glen; Pyke, Robert; Segraves, R. Taylor; Leiblum, Sandra; Clayton, Anita; Cotton, Dan; Evans, Kenneth	2005	The Sexual Interest and Desire Inventory--Female (SIDI-F): Item Response Analyses of Data from Women Diagnosed with Hypoactive Sexual Desire Disorder	10.1111/j.1743-6109.2005.00146.x
Population	Stokes, M. A.; Kaur, A.	2005	High-functioning autism and sexuality: A parental perspective	10.1177/1362361305053258
Population	Uribe Rodríguez, A. F.	2005	Evaluation of the knowledge on sexual abuse in minors and their strategies of prevention in teachers	N/A
Population	Birnbaum, Gurit E.; Gillath, Omri	2006	Measuring subgoals of the sexual behavioral system: What is sex good for?	10.1177/0265407506065992
Population	Hamby, Sherry; Sugarman, David B.; Boney-McCoy, Sue	2006	Does questionnaire format impact reported partner violence rates?: An experimental study	10.1891/vivi.21.4.507
Population	Lanier, C. A.; Green, B. A.	2006	Principal component analysis of the College Date Rape Attitude Survey (CDRAS): An instrument for the evaluation of date rape prevention programs	10.1300/J146v13n02_06
Population	Lauby, Jennifer L.; Bond, Lisa; Eroğlu, Dogan; Batson, Heather	2006	Decisional Balance, Perceived Risk and HIV Testing Practices	10.1007/s10461-005-9029-7
Population	Ongen, Demet	2006	Atttudes towards Women: A Study of Gender and Academic Domain Differences in a Sample of Turkish University Students	
Population	Ortega, Virgilio; Zubeidat, Ihab; Sierra, Juan Carlos	2006	Further Examination of Measurement Properties of Spanish Version of the Sexual Desire Inventory with Undergraduates and Adolescent Students	10.2466/PR0.99.5.147-165
Population	Renaud, Cheryl A.; Byers, Sandra E.	2006	Positive and Negative Cognitions of Sexual Submission: Relationship to Sexual Violence	10.1007/s10508-006-9046-2
Population	Rosenbaum, Alan; Rabenhorst, Mandy M.; Reddy, Madhavi K.; Fleming, Matthew T.; Howells, Nicolette L.	2006	A Comparison of Methods for Collecting Self-Report Data on Sensitive Topics	10.1891/vivi.21.4.461
Population	Terry, Paul E.; Mhloyi, Marvelous; Masvaure, Tsitsi; Adlis, Susan	2006	An examination of knowledge, attitudes and practices related to HIV/AIDS prevention in Zimbabwean university students: comparing intervention program participants and non-participants	10.1016/j.ijid.2004.10.007
Population	Burgess, Gerald H.	2007	Assessment of rape-supportive attitudes and beliefs in college men: Development, reliability, and validity of the Rape Attitudes and Beliefs Scale	10.1177/0886260507302993
Population	Green, Lauren Ashley	2007	Undergraduate students' motivations to engage in sexual behaviors after consuming alcohol: A mixed methodological action research approach	N/A
Population	Hess, Rosanna F.; McKinney, Dawn	2007	Fatalism and HIV/AIDS beliefs in rural Mali, West Africa	10.1111/j.1547-5069.2007.00155.x
Population	Horvath, Keith J.; Beadnell, Blair; Bowen, Anne M.	2007	A daily Web diary of the sexual experiences of men who have sex with men: Comparisons with a retrospective recall survey	10.1007/s10461-007-9206-y
Population	Humphreys, Terry; Herold, Ed	2007	Sexual consent in heterosexual relationships: Development of a new measure	10.1007/s11199-007-9264-7
Population	Jaworski, Beth C.; Carey, Michael P.	2007	Development and psychometric evaluation of a self-administered questionnaire to measure knowledge of sexually transmitted diseases	10.1007/s10461-006-9168-5
Population	Lam, C. B.; Chan, D.K.-S.	2007	The use of cyberpornography by young men in Hong Kong: Some psychosocial correlates	10.1007/s10508-006-9124-5
Population	Meston, Cindy M.; Buss, David M.	2007	Why humans have sex	10.1007/s10508-007-9175-2
Population	Sierra, Juan Carlos; Delgado-Domínguez, Carlos J.; Gutiérrez-Quintanilla, José RIcardo	2007	Escala de Actitud Favorable hacia la Violación: Primeras evidencias acerca de su fiabilidad y validez en muestras Salvadoreñas = Rape Supportive Attitude Scale: First evidence on its reliability and validity in the Salvadorian samples	10.1037/t57716-000;

Reason for Exclusion/ Inclusion	Author(s)	Year	Title	DOI
Population	Sierra, Juan Carlos; Rojas, Antonio; Ortega, Virgilio; Ortiz, Juan Domingo Martín	2007	Evaluación de actitudes machistas en universitarios: Primeros datos psicométricos de las versiones españolas de la Double Standard Scale (DSS) y de la Rape Supportive Attitude Scale (RSAS) = Evaluation of chauvinistic attitudes in college students: First psychometric data of the Spanish versions of Double Standard Scale (DSS) y de la Rape Supportive Attitude Scale (RSAS)	10.1037/t06548-000;
Population	Boyer, Cherrie B.; Pollack, Lance M.; Becnel, Jennifer; Shafer, Mary-Ann	2008	Relationships among sociodemographic markers, behavioral risk, and sexually transmitted infections in U. S. female Marine Corps recruits	10.7205/milmed.173.11.1078
Population	Cárdenas, Manuel; Barrientos, Jaime Eduardo	2008	The Attitudes Toward Lesbians and Gay Men Scale (ATLG): Adaptation and Testing the Reliability and Validity in Chile	10.1080/00224490801987424
Population	Cox, Mary Foster; Fasolino, Tracy K.; Tavakoli, Abbas S.	2008	Factor analysis and psychometric properties of the Mother-Adolescent Sexual Communication (MASC) instrument for sexual risk behavior	10.1891/1061-3749.16.3.171
Population	Dunphy, K. P.	2008	The law concerning teenage sex: Do we understand it?	10.1258/ijsa.2007.007130
Population	Feelgood, S.; Schaefer, G. A.; Hoyer, J.	2008	Skala zur Erfassung kognitiver Verzerrungen bei Missbrauchern	10.23668/psycharchives.4745.
Population	Frayman, K. B.; Cerritelli, B.; Wilson, J.; Sawyer, S. M.	2008	Reproductive and sexual health in boys with cystic fibrosis: What do parents know and say?	10.1002/ppul.20911
Population	Galupo, M. Paz; Pearl, Marcia L.	2008	Bisexual attitudes toward same-sex marriage	10.1080/15299710802171357
Population	Hormes, J. M.; La Lytle; Gross, C. R.; Ahmed, R. L.; Troxel, AB; Schmitz, K. H.	2008	The body image and relationships scale: Development and validation of a measure of body image in female breast cancer survivors	10.1200/JCO.2007.14.2661
Population	Marcus, Robert F.	2008	Fight-Seeking Motivation in Dating Partners With an Aggressive Relationship	10.3200/SOCP.148.3.261-276
Population	Odu, O. O.; Asekun-Olarinmoye, E. O.; Bamidele, J. O.; Egbewale, B. E.; Amusan, O. A.; Olowu, A. O.	2008	Knowledge, attitudes to HIV/AIDS and sexual behaviour of students in a tertiary institution in south-western Nigeria	10.1080/13625180701617670
Population	Pulerwitz, Julie; Barker, Gary	2008	Measuring attitudes toward gender norms among young men in Brazil: Development and psychometric evaluation of the GEM scale	10.1177/1097184X06298778
Population	Schuler, Sidney Ruth; Islam, Farzana	2008	Women's acceptance of intimate partner violence within marriage in rural Bangladesh	10.1111/j.1728-4465.2008.00150.x
Population	Vik, Peter W.; Islam-Zwart, Kayleen A.; Ruge, Laura N.	2008	Application of the PTSD-alcohol expectancy questionnaire (P-AEQ) to sexually assaulted college women	10.1080/16066350701867273
Population	Angelone, David J.; Mitchell, Damon; Carola, Kara	2009	Tolerance of sexual harassment: a laboratory paradigm	10.1007/s10508-008-9421-2
Population	Bauermeister, José A.; Carballo-Diéguez, Alex; Ventuneac, Ana; Dolezal, Curtis	2009	Assessing motivations to engage in intentional condomless anal intercourse in HIV risk contexts ('bareback sex') among men who have sex with men	10.1521/aeap.2009.21.2.156
Population	Brennan, David J.; Welles, Seth L.; Miner, Michael H.; Ross, Michael W.; Mayer, Kenneth H.; Rosser, B. R.	2009	Development of a treatment optimism scale for HIV-positive gay and bisexual men	10.1080/09540120802705859
Population	Del Castillo, Cinthia Cruz; Díaz-Loving, Rolando; Nieto, Erika Miranda	2009	Construcción de una escala sobre normas y valores en universitarios Mexicanos = Development of a norms and beliefs scale for Mexican college students	N/A
Population	Doyle, Suzanne R.; Calsyn, Donald A.; Ball, Samuel A.	2009	Factor structure of the Condoms Barriers Scale with a sample of men at high risk for HIV	10.1177/1073191108322259
Population	Gari, Aikaterini; Georgouleas, George; Giotsa, Artemis; Stathopoulou, Eleni-Anna	2009	Greek students' attitudes toward rape	10.1037/t61993-000;
Population	Jones, Rachel; Gulick, Elsie	2009	Reliability and validity of the Sexual Pressure Scale for Women-Revised	10.1002/nur.20297
Population	Morrison, T. G.; McDermott, D. T.	2009	Psychometric properties of the support for lesbian and gay human rights scale	10.3200/SOCP.149.2.263-266
Population	Moyer, Christopher A.; Rounds, James	2009	The attitudes toward massage (ATOM) scale: Reliability, validity, and associated findings	10.1016/j.jbmt.2008.01.002
Population	Ogle, Richard L.; Noel, Nora E.; Maisto, Stephen A.	2009	Assessing acceptance of violence toward women: A factor analysis of Burt's Acceptance of Interpersonal Violence Scale	10.1177/1077801209334444
Population	Prentky, Robert A.; Pimental, Ann; Cavanaugh, Deborah J.; Righthand, Sue	2009	Predicting risk of sexual recidivism in juveniles: Predictive validity of the J-SOAP-II	10.1037/t00800-000.
Population	Rosenberg, Rebecca E.; Ahmed, A S M Nawshad U; Ahmed, Saifuddin; Saha, Samir K.; Chowdhury, M A K Azad; Black, Robert E.; Santosham, Mathuram; Darmstadt, Gary L.	2009	Determining Gestational Age in a Low-resource Setting: Validity of Last Menstrual Period	10.3329/jhpn.v27i3.3375

Reason for Exclusion/ Inclusion	Author(s)	Year	Title	DOI
Population	Sawyer, Steven P.; Metz, Michael E.	2009	The Attitudes Toward Prostitution Scale: Preliminary report on its development and use	10.1177/0306624X08316706
Population	Turner, an; Kock, A. E. de; Meehan-Ritter, A.; Blanchard, K.; Sebola, M. H.; Hoosen, A. A.; Coetzee, N.; Ellertson, C.	2009	Many vaginal microbicide trial participants acknowledged they had misreported sensitive sexual behavior in face-to-face interviews	10.1016/j.jclinepi.2008.07.011
Population	Vanable, Peter A.; Carey, Michael P.; Brown, Jennifer L.; DiClemente, Ralph J.; Salazar, Laura F.; Brown, Larry K.; Romer, Daniel; Valois, Robert F.; Hennessy, Michael; Stanton, Bonita F.	2009	Test-retest reliability of self-reported HIV/STD-related measures among African-American adolescents in four U.S. cities	10.1016/j.jadohealth.2008.09.002
Population	Anwar, Mudassir; Sulaiman, Syed A. Syed; Khan, Tahir M.	2010	A survey of knowledge of sexually transmitted infections among patients at a public hospital in Pulau Pinang, Malaysia	10.1159/000312719
Population	Atherton, H.; Oakeshott, P.; Aghaizu, A.; Hay, P.; Kerry, S.	2010	Use of an online questionnaire for follow-up of young female students recruited to a randomised controlled trial of chlamydia screening	10.1136/jech.2009.098830
Population	Bornovalova, Marina A.; Daughters, Stacey B.; Lejuez, Carl W.	2010	Motivations for sexual risk behavior across commercial and casual partners among male urban drug users: Contextual features and clinical correlates	10.1177/0145445510364414
Population	Chonody, Jill M.	2010	Exploring sexual prejudice in context: History, theory, and measurement	N/A
Population	Fergus, Stevenson	2010	Review of Unequal opportunity: Health disparities affecting gay and bisexual men in the United States	N/A
Population	Humphreys, Terry P.; Brousseau, Mélanie M.	2010	The Sexual Consent Scale-Revised: Development, reliability, and preliminary validity	10.1080/00224490903151358
Population	McRee, A.-L.; Brewer, N. T.; Reiter, P. L.; Gottlieb, S. L.; Smith, J. S.	2010	The Carolina HPV Immunization Attitudes and Beliefs Scale (CHIAS): Scale development and associations with intentions to vaccinate	10.1097/OLQ.0b013e3181c37e15
Population	Meyer-Bahlburg, Heino F. L.; Dolezal, Curtis; Johnson, Laurel L.; Kessler, Suzanne J.; Schober, Justine M.; Zucker, Kenneth J.	2010	Development and validation of the Pregnancy and Infant Orientation Questionnaire	10.1080/00224490903244567
Population	Pratte, Katherine; Whitesell, Nancy; McFarlane, Mary; Bull, Sheana	2010	Factor analyses of condom attitudes, norms, and self-efficacy measures in diverse samples	10.1891/1061-3749.18.3.153
Population	Rama, C. H.; Villa, L. L.; Pagliusi, S.; Andreoli, M. A.; Costa, M. C.; Aoki, A. L.; Longatto-Filho, A.; Eluf-Neto, J.	2010	Awareness and knowledge of HPV, cervical cancer, and vaccines in young women after first delivery in São Paulo, Brazil - a cross-sectional study	10.1186/1472-6874-10-35
Population	Renju, J.; Andrew, B.; Nyalali, K.; Kishamawe, C.; Kato, C.; Changalucha, J.; Obasi, A.	2010	A process evaluation of the scale up of a youth-friendly health services initiative in northern Tanzania	10.1186/1758-2652-13-32
Population	Rye, B. J.; Meaney, Glenn J.	2010	Measuring homonegativity: A psychometric analysis	10.1037/a0018237
Population	Shacham, Enbal; Cottler, Linda B.	2010	Sexual behaviors among club drug users: Prevalence and reliability	10.1007/s10508-009-9539-x
Population	Singh, M.; Kotwal, A.; Gupta, R. M.; Adhya, S.; Chatterjee, K.; Jayaram, J.	2010	Sero-Epidemiological and Behavioural Survey of HIV, HBV and HCV amongst Indian Armed Forces Trainees	10.1016/S0377-1237(10)80093-0
Population	Smolenski, Derek J.; Diamond, Pamela M.; Ross, Michael W.; Rosser, B. R. Simon	2010	Revision, criterion validity, and multigroup assessment of the Reactions to Homosexuality Scale	10.1080/00223891.2010.513300
Population	Tilahun, D.; Assefa, T.; Belachew, T.	2010	Predictors of emergency contraceptive use among regular female students at adama university, central Ethiopia	N/A
Population	Walker, Denise D.; Neighbors, Clayton; Mbilinyi, Lyungai F.; O'Rourke, Allison; Zegree, Joan; Roffman, Roger A.; Edleson, Jeffrey L.	2010	Evaluating the impact of intimate partner violence on the perpetrator: The Perceived Consequences of Domestic Violence Questionnaire	10.1177/0886260509354592
Population	Weston, R. L.; Hopwood, B.; Harding, J.; Sizmur, S.; Ross, J. D. C.	2010	Development of a validated patient satisfaction survey for sexual health clinic attendees	10.1258/ijsa.2010.010159
Population	Agardh, Anette; Tumwine, Gilbert; Östergren, Per-Olof	2011	The impact of socio-demographic and religious factors upon sexual behavior among Ugandan university students	10.1371/journal.pone.0023670
Population	Balogun, J.; Abiona, T.; Lukobo-Durrell, M.; Adefuye, A.; Amosun, S.; Frantz, J.; Yakut, Y.	2011	Readability and test-retest reliability of a psychometric instrument designed to assess HIV/AIDS attitudes, beliefs, behaviours and sources of HIV prevention information of young adults	10.1177/0017896910373022

Reason for Exclusion/ Inclusion	Author(s)	Year	Title	DOI
Population	Balogun, Joseph; Abiona, Titilayo; Lukobo-Durrell, Mainza; Adefuye, Adedeji; Amosun, Seyi; Frantz, Jose; Yakut, Yavuz	2011	Readability and Test-Retest Reliability of a Psychometric Instrument Designed to Assess HIV/AIDS Attitudes, Beliefs, Behaviours and Sources of HIV Prevention Information of Young Adults	10.1177/0017896910373022
Population	Brown, Michael J.; Henriquez, Ernesto	2011	Support for gay and lesbian civil rights: Development and examination of a new scale	10.1080/00918369.2011.555664
Population	Chankapa, Y. D.; Pal, R.; Tsering, D.	2011	Correlates of cervical cancer screening among underserved women	10.4103/0019-509X.75823
Population	Davidovic, Anna; Bell, Kurtis; Ferguson, Colin; Gorski, Elizabeth; Campbell, Anne	2011	Impelling and inhibitory forces in aggression: Sex-of-target and relationship effects	10.1177/0886260510390953
Population	DiClemente, Ralph J.; Sales, Jessica McDermott; Danner, Fred; Crosby, Richard A.	2011	Association between sexually transmitted diseases and young adults' self-reported abstinence	10.1542/peds.2009-0892
Population	Donaldson, Robyn L.	2011	To consult or not to consult? Investigating barriers to dyspareunia treatment-seeking in young women	10.34917/2344937
Population	Hollub, Ariane V.; Reece, Michael; Herbenick, Debby; Hensel, Devon J.; Middlestadt, Susan E.	2011	College students and condom attitude: validation of the Multi-Factor Attitude toward Condoms Scale (MFACS)	10.1080/07448481.2010.546462
Population	Janda, Louis H.; Bazemore, Sharnail D.	2011	The revised Mosher Sex-Guilt Scale: Its psychometric properties and a proposed ten-item version	10.1080/00224499.2010.482216
Population	Lehavot, Keren; King, Kevin M.; Simoni, Jane M.	2011	Development and validation of a gender expression measure among sexual minority women	10.1177/0361684311413554
Population	Li, Qing; Li, Xiaoming; Stanton, Bonita; Wang, Bo	2011	Psychometric properties of a Pictorial Scale Measuring Correct Condom use	10.1007/s10461-010-9838-1
Population	Liss, Miriam; Erchull, Mindy J.; Ramsey, Laura R.	2011	Empowering or oppressing? Development and exploration of the Enjoyment of Sexualization Scale	10.1177/0146167210386119
Population	Mohr, Jonathan J.; Kendra, Matthew S.	2011	Revision and extension of a multidimensional measure of sexual minority identity: the Lesbian, Gay, and Bisexual Identity Scale	10.1037/a0022858
Population	Mohr, Jonathan J.; Kendra, Matthew S.	2011	Revision and extension of a multidimensional measure of sexual minority identity: the Lesbian, Gay, and Bisexual Identity Scale	10.1037/a0022858
Population	Mullens, Amy B.; Young, Ross McD; Dunne, Michael P.; Norton, Graham	2011	The Drinking Expectancy Questionnaire for Men who have Sex with Men (DEQ-MSM): a measure of substance-related beliefs	10.1111/j.1465-3362.2010.00225.x
Population	Nöstlinger, Christiana; Nideröst, Sibylle; Platteau, Tom; Müller, Matthias C.; Staneková, Danica; Gredig, Daniel; Roulin, Christophe; Rickenbach, Martin; Colebunders, Robert	2011	Sexual protection behavior in HIV-positive gay men: Testing a modified information-motivation-behavioral skills model	10.1007/s10508-010-9682-4
Population	Reid, Rory C.; Li, Desiree S.; Gilliland, Randy; Stein, Judith A.; Fong, Timothy	2011	Reliability, validity, and psychometric development of the Pornography Consumption Inventory in a sample of hypersexual men	10.1080/0092623X.2011.607047
Population	Royer, Heather Rhea	2011	Young women's representations of sexually transmitted infections and sexually transmitted infection testing	10.1002/nur.21452
Population	Rull, Marco Antonio Pulido; Cardona, Verónica Carazo; Sicilia, Gabriela Orta González; Villalobos, Mauricio Coronel; García, Fernando Vera	2011	Conducta sexual de riesgo en los estudiantes de licenciatura de la Universidad Intercontinental = Risky sexual behavior in undergraduate students of the Intercontinental University	N/A
Population	Summers, Bryce B.	2011	Factor structure and validity of the Lesbian, Gay, Bisexual Knowledge and Attitude Scale for Heterosexuals (LGB-KASH)	N/A
Population	Tafuri, S.; Martinelli, D.; Germinario, C.; Prato, R.	2011	A study on the sexual and contraception behaviours of the pre-university students in Puglia (South-Italy)	N/A
Population	Travison, Thomas G.; Sand, Michael S.; Rosen, Raymond C.; Shabsigh, Ridwan; Eardley, Ian; McKinlay, John B.	2011	The natural progression and regression of erectile dysfunction: follow-up results from the MMAS and MALES studies	10.1111/j.1743-6109.2011.02294.x
Population	Wilson, Karen; Mattingly, Brent A.; Clark, Eddie M.; Weidler, Daniel J.; Bequette, Amanda W.	2011	The gray area: Exploring attitudes toward infidelity and the development of the Perceptions of Dating Infidelity Scale	10.1080/00224540903366750
Population	Yi, Huso; Shidlo, Ariel; Sandfort, Theo	2011	Assessing maladaptive responses to the stress of being at risk of HIV infection among HIV-negative gay men in New York City	10.1080/00224490903487570

Reason for Exclusion/ Inclusion	Author(s)	Year	Title	DOI
Population	Berg, R. C.; Ross, M. W.; Schmidt, A. J.; Weatherburn, P.	2012	Structural determinants in MSM HIV preventionenvironmental and structural factors predict internalised homonegativity in men who have sex with men (MSM): Findings from the European MSM internet survey (EMIS) in 38 countries	10.7448/IAS.15.5.18438
Population	Brase, Gary L.; Brase, Sandra L.	2012	Emotional regulation of fertility decision making: What is the nature and structure of 'baby fever'?	10.1037/a0024954
Population	Brewster, Melanie E.; Velez, Brandon; DeBlaere, Cirleen; Moradi, Bonnie	2012	Transgender individuals' workplace experiences: The applicability of sexual minority measures and models	10.1037/a0025206
Population	Callahan, Rebecca L.; Becker, Stan	2012	The reliability of calendar data for reporting contraceptive use: Evidence from rural Bangladesh	10.1111/j.1728-4465.2012.00319.x
Population	Carlson, Thomas Stone; McGeorge, Christi R.; Toomey, Russell B.	2012	Establishing the Validity of the Affirmative Training Inventory: Assessing the Relationship between Lesbian, Gay, and Bisexual Affirmative Training and Students' Clinical Competence	10.1111/j.1752-0606.2012.00286.x
Population	Delgado, J. E.B.; Castro, M. C.	2012	A confirmatory factor analysis of the spanish language version of the Attitudes Toward Lesbians and Gay Men Scale (ATLG)	N/A
Population	Gowda, C.; Carlos, R. C.; Butchart, A. T.; Singer, D. C.; Davis, M. M.; Clark, S. J.; Dempsey, A. F.	2012	CHIAS: A standardized measure of parental HPV immunization attitudes and beliefs and its associations with vaccine uptake	10.1097/OLQ.0b013e318248a6d5
Population	Huang, Cheng-Yi; Tsai, Li-Ya; Liao, Wen-Chun; Lee, Sheuan	2012	Nursing interventions on sexual health: validation of the NISH Scale in baccalaureate nursing students in Taiwan	10.1111/j.1743-6109.2012.02784.x
Population	Jong, M. G. de; Pieters, R.; Stremersch, S.	2012	Analysis of sensitive questions across cultures: an application of multigroup item randomized response theory to sexual attitudes and behavior	10.1037/a0029394
Population	Krishnamurti, Tamar; Loewenstein, George	2012	The partner-specific sexual liking and sexual wanting scale: Psychometric properties	10.1007/s10508-011-9785-6
Population	Lingiardi, Vittorio; Baiocco, Roberto; Nardelli, Nicola	2012	Measure of internalized sexual stigma for lesbians and gay men: A new scale	10.1080/00918369.2012.712850
Population	Mandarelli, Gabriele; Zangaro, Stefania; Raja, Michele; Azzoni, Antonella; Tatarelli, Roberto; Ferracuti, Stefano	2012	Competence to consent to sexual activity in bipolar disorder and schizophrenic spectrum disorders	10.1007/s10508-011-9840-3
Population	McCallum, Ethan B.; Peterson, Zoë D.; Mueller, Tiffany M.	2012	Validation of the traumatic sexualization survey for use with heterosexual men	10.1080/00224499.2011.585524
Population	Napper, Lucy E.; Fisher, Dennis G.; Reynolds, Grace L.	2012	Development of the Perceived Risk of HIV scale	10.1007/s10461-011-0003-2
Population	Nunes, Kevin L.; Babchishin, Kelly M.	2012	Construct validity of Stable-2000 and Stable-2007 scores	10.1177/1079063211404921
Population	Oliveira, João Manuel de; Lopes, Diniz; Costa, Carlos Gonçalves; Nogueira, Conceição	2012	Lesbian, Gay, and Bisexual Identity Scale (LGBIS): Construct validation, sensitivity analyses and other psychometric properties	10.5209/rev_SJOP.2012.v15.n1.37340
Population	Riley, Bettina Hornbuckle	2012	Parental influences on late adolescents' autonomous motivation and sexual risk knowledge and behavior	N/A
Population	Royer, Heather R.; Heidrich, Susan M.; Brown, Roger L.	2012	Young women's Representations of Sexually Transmitted Diseases (RoSTD): a psychometric study	10.1002/nur.21452
Population	Tebbe, Esther N.; Moradi, Bonnie	2012	Anti-transgender prejudice: A structural equation model of associated constructs	10.1037/a0026990
Population	Warthe, Diane Gaye Watson	2012	The development of the Dating Relationship Scales for young adults	N/A
Population	Arseneau, Julie R.; Grzanka, Patrick R.; Miles, Joseph R.; Fassinger, Ruth E.	2013	Development and initial validation of The Sexual Orientation Beliefs Scale (SOBS)	10.1037/a0032799
Population	Asare, Matthew; Sharma, Manoj; Bernard, Amy L.; Rojas-Guyler, Liliana; Wang, Lihshing Leigh	2013	Using the health belief model to determine safer sexual behavior among African immigrants	10.1353/hpu.2013.0020
Population	Aubrey, Jennifer Stevens; Smith, Siobhan E.	2013	Development and validation of the endorsement of the hookup culture index	10.1080/00224499.2011.637246
Population	Baggio, S.; Studer, J.; Daeppen, J-B; Gmel, G.	2013	Adaptation of a peer pressure scale in French and German: the Peer Pressure Inventory	10.1016/j.respe.2012.12.016
Population	Barrientos, Jaime; Cárdenas, Manuel; Gómez, Fabiola; Frías-navarro, Dolores	2013	Assessing the Dimensionality of Beliefs About Children's Adjustment in Same-Sex Families Scale (BCASSFS) in Chile	10.1007/s13178-012-0107-0
Population	Carey, M. P.; La Scott-Sheldon; Senn, T. E.; Carey, K. B.	2013	Attitudes toward sexual partner concurrency: development and evaluation of a brief, self-report measure for field research	10.1007/s10461-012-0346-3
Population	Chonody, Jill M.	2013	Measuring sexual prejudice against gay men and lesbian women: development of the Sexual Prejudice Scale (SPS)	10.1080/00918369.2013.774863

Reason for Exclusion/ Inclusion	Author(s)	Year	Title	DOI
Population	Davidson, M. Meghan; Gervais, Sarah J.; Canivez, Gary L.; Cole, Brian P.	2013	A psychometric examination of the Interpersonal Sexual Objectification Scale among college men	10.1037/a0032075
Population	Dickstein, Jodi B.; Goldstein, Sue W.; Tkachenko, Natalia; Kreppner, Wayne	2013	Correlation of question 15 of the FSDS–DAO with clinician evaluation of female orgasmic disorder	10.1111/jsm.12218
Population	Gorbach, Pamina M.; Mensch, Barbara S.; Husnik, Marla; Coly, Astou; Mâsse, Benoit; Makanani, Bonus; Nkhoma, Chiwawa; Chinula, Lameck; Tembo, Tchangani; Mierzwa, Stan; Reynolds, Kimberly; Hurst, Stacey; Coletti, Anne; Forsyth, Andrew	2013	Effect of computer-assisted interviewing on self-reported sexual behavior data in a microbicide clinical trial	10.1007/s10461-012-0302-2
Population	Grover, Rachel L.; Nangle, Douglas W.; Serwik, Agnieszka K.; Fales, Jessica; Prenoveau, Jason M.	2013	The measure of heterosocial competence: Development and psychometric investigation	10.1177/0265407512458658
Population	Hayes, Eden-Reneé; Swim, Janet K.	2013	African, Asian, Latina/o, and European Americans' responses to popular measures of sexist beliefs: Some cautionary notes	10.1177/0361684313480044
Population	Kruger, Daniel J.; Fisher, Maryanne L.; Edelstein, Robin S.; Chopik, William J.; Fitzgerald, Carey J.; Strout, Sarah L.	2013	Was that cheating? Perceptions vary by sex, attachment anxiety, and behavior	10.1177/147470491301100115
Population	Krupp, Kerstin; Brunner, Franziska; Fliegner, Maike; Rall, Katharina; Brucker, Sara; Briken, Peer; Richter-Appelt, Hertha	2013	Fragebogen zum Erleben der eigenen Weiblichkeit (FB-W): Ergebnisse von Frauen mit Mayer-Rokitansky-Küster-Hauser Syndrom und Frauen mit polyzystischem Ovarsyndrom = A questionnaire for the assessment of women's perception of their own femininity: A study on women with Mayer-Rokitansky-Kuster-Hauser-syndrome and women with polycystic ovary syndrome	10.1055/s-0032-1333286
Population	Maleki, Azam; Mazloomzadeh, Saeideh	2013	Women's perceived internal control of future pregnancy outcomes and its related factors	N/A
Population	Mitchell-Box, Kristen; Braun, Kathryn L.; Hurwitz, Eric L.; Hayes, Donald K.	2013	Breastfeeding attitudes: Association between maternal and male partner attitudes and breastfeeding intent	10.1089/bfm.2012.0135
Population	Mousavi, Abbas; Keramat, Afsaneh; Vakilian, Katayon; Esmaeili Vardanjani, Safar Ali	2013	Development and adaptation of Iranian youth reproductive health questionnaire	10.1155/2013/950278
Population	Mullany, Britta; Barlow, Allison; Neault, Nicole; Billy, Trudy; Hastings, Ranelda; Coho-Mescal, Valerie; Lorenzo, Sherilyn; Walkup, John T.	2013	Consistency in the reporting of sensitive behaviors by adolescent American Indian women: A comparison of interviewing methods	10.5820/aian.2002.2013.42
Population	Riggio, Heidi R.; Weiser, Dana A.; Valenzuela, Ann Marie; Lui, P. Priscilla; Montes, Roberto; Heuer, Julie	2013	Self-efficacy in romantic relationships: Prediction of relationship attitudes and outcomes	10.1080/00224545.2013.801826
Population	Rocca, Corinne H.; Harper, Cynthia C.; Raine-Bennett, Tina R.	2013	Young women's perceptions of the benefits of childbearing: associations with contraceptive use and pregnancy	10.1363/4502313
Population	Ugarte, William J.; Högberg, Ulf; Valladares, Eliette C.; Essén, Birgitta	2013	Measuring HIV-and AIDS-related stigma and discrimination in Nicaragua: Results from a community-based study	10.1521/aeap.2013.25.2.164
Population	van Overveld, Mark; Jong, Peter J. de; Peters, Madelon L.; van Lankveld, Jacques; Melles, Reinhilde; Kuile, Moniek M. ter	2013	The Sexual Disgust Questionnaire; A psychometric study and a first exploration in patients with sexual dysfunctions	10.1111/j.1743-6109.2012.02979.x
Population	Varkovitzky, Ruth Luba	2013	Assimilation, accommodation, and overaccommodation: An examination of information processing styles in female victims of adolescent and adult sexual assault	N/A
Population	Versteegh, Hendt Paul; Bakia, Affuenti; Koopman, Hendrik Maria; Kraaij, Vivian; Versteegh, Florens Gerard Adriaan	2013	Evaluation of HIV and AIDS knowledge in rural Cameroon men with the use of a questionnaire	10.11604/pamj.2013.16.141.2964
Population	White, Jacquelyn W.; Yuan, Nicole P.; Cook, Sarah L.; Abbey, Antonia	2013	Ethnic minority women’s experiences with intimate partner violence: Using community-based participatory research to ask the right questions	10.1007/s11199-012-0237-0
Population	Alexander, Angel M.; Flynn, Kathryn E.; Hahn, Elizabeth A.; Jeffery, Diana D.; Keefe, Francis J.; Reeve, Bryce B.; Schultz, Wesley; Reese, Jennifer Barsky; Shelby, Rebecca A.; Weinfurt, Kevin P.	2014	Improving patients' understanding of terms and phrases commonly used in self-reported measures of sexual function	10.1111/jsm.12599
Population	Armstrong, Heather L.	2014	Sexual attitudes and motivations in same-sex and mixed-sex relationships	N/A

Reason for Exclusion/ Inclusion	Author(s)	Year	Title	DOI
Population	Banyard, Victoria L.; Moynihan, Mary M.; Cares, Alison C.; Warner, Rebecca	2014	How do we know if it works? Measuring outcomes in bystander-focused abuse prevention on campuses	10.1037/a0033470
Population	Bastings, L.; Baysal, Ö.; Beerendonk, C. C. M.; IntHout, J.; Traas, M. A. F.; Verhaak, C. M.; Braat, D. D. M.; Nelen, W L D M	2014	Deciding about fertility preservation after specialist counselling	10.1093/humrep/deu136
Population	Birnbaum, Gurit E.; Mikulincer, Mario; Szepsenwol, Ohad; Shaver, Phillip R.; Mizrahi, Moran	2014	When sex goes wrong: A behavioral systems perspective on individual differences in sexual attitudes, motives, feelings, and behaviors	10.1037/a0036021
Population	Gallo, Agatha M.; Wilkie, Diana J.; Wang, Edward; Labotka, Richard J.; Molokie, Robert E.; Stahl, Christiane; Hershberger, Patricia E.; Zhao, Zhongsheng; Suarez, Marie L.; Johnson, Bonnye; Pullum, Cherese; Angulo, Rigoberto; Thompson, Alexis	2014	Evaluation of the SCKnowIQ tool and reproductive CHOICES intervention among young adults with sickle cell disease or sickle cell trait	10.1177/1054773813479377
Population	Gato, Jorge; Fontaine, Anne Marie; Leme, Vanessa B. R. L.	2014	Validação e adaptação transcultural da Escala Multidimensional de Atitudes Face a Lésbicas e a Gays = Validation and transcultural adaptation of the Multidimensional Scale of Attitudes toward Lesbians and Gay Men	10.1590/1678-7153.201427206
Population	Grover, Sandeep; Avasthi, Ajit; Aneja, Jitender; Shankar, Gauri; Mohan M, Ravi; Nehra, Ritu; Padhy, Sushanta K.	2014	Comprehensive questionnaire for assessment of dhat syndrome: Development and use in patient population	10.1111/jsm.12241
Population	Hans, Jason D.; Kimberly, Claire	2014	Abortion attitudes in context: A multidimensional vignette approach	10.1016/j.ssresearch.2014.06.001
Population	Kenney, Shannon R.; Lac, Andrew; Hummer, Justin F.; LaBrie, Joseph W.	2014	Development and validation of the Hookup Motives Questionnaire (HMQ)	10.1037/a0037131
Population	Kimberly, Claire; Werner-Wilson, Ronald; Motes, Zachary	2014	Brief report: Expanding the Brief Sexual Attitudes Scale	10.1007/s13178-013-0124-7
Population	Leonard, L. E.; Germain, A.; White-Jones, K.; Poncia, A.; Vannice, S.	2014	The voices of Ottawa youth who smoke crack: Urgent need for age-specific HIV- and HCV related prevention interventions	N/A
Population	Lick, David J.; Johnson, Kerri L.	2014	'You can't tell just by looking!': Beliefs in the diagnosticity of visual cues explain response biases in social categorization	10.1177/0146167214549323
Population	Liou, W.-Y.	2014	An illustrated scale measuring the sexual-abuse prevention knowledge of female high school students with intellectual disabilities in Taiwan	10.1007/s11195-013-9312-x
Population	McMahon, Sarah; Allen, Christopher T.; Postmus, Judy L.; McMahon, Sheila M.; Peterson, N. Andrew; Lowe Hoffman, Melanie	2014	Measuring bystander attitudes and behavior to prevent sexual violence	10.1080/07448481.2013.849258
Population	Morris, Jessica L.; Short, Samm; Robson, Laura; Andriatsihosena, Mamy Soafaly	2014	Maternal health practices, beliefs and traditions in southeast Madagascar	N/A
Population	Zhou, Yongkang; Zeng, Weixi; Hu, Yalin	2014	Sexual-moral attitudes of Chinese college students: Two moral criteria systems?	10.4236/psych.2014.52018
Population	Chelli, L.; Riquet, S.; Perrin, J.; Courbiere, B.	2015	Should we better inform young women about fertility? A state-of-knowledge study in a student population	10.1016/j.gyobfe.2015.01.002
Population	Delgado, Jaime Barrientos; Cárdenas, Manuel; Estrada, Claudia; Adaos, Romina; Carvajal, Juan; Peña, Mitzio; Villar, Javiera	2015	Adaptation and validation of the Polymorphous Prejudice Scale (PPS)-A short form in a sample of heterosexual Chilean college students	10.1080/19317611.2014.957795
Population	Dias, H. M.D.; Sim-Sim, MMSF	2015	Validation of the Sexuality Attitudes and Beliefs Survey (SABS) for the Portuguese population	10.1590/1982-0194201500034
Population	Dillon, Frank R.; Alessi, Edward J.; Craig, Shelley; Ebersole, Ryan C.; Kumar, Snehal M.; Spadola, Christine	2015	Development of the Lesbian, Gay, and Bisexual Affirmative Counseling Self-Efficacy Inventory – Short Form (LGB-CSI-SF)	10.1037/sgd0000087
Population	Doshi, Dolar; Reddy, B. Srikanth; Karunakar, P.; Deshpande, Kopparesh	2015	HPV, Cervical Cancer and Pap Test Related Knowledge Among a Sample of Female Dental Students in India	10.7314/apjcp.2015.16.13.5415
Population	Erchull, Mindy J.; Liss, Miriam	2015	Clinical outcomes of enjoying sexualization among lesbian women	10.1080/00918369.2014.972808
Population	Grauvogl, Andrea; Peters, Madelon L.; Evers, Silvia M A A; van Lankveld, Jacques J D M	2015	A new instrument to measure sexual competence and interaction competence in youth: psychometric properties in female adolescents	10.1080/0092623X.2014.933461
Population	Habarth, Janice M.	2015	Development of the Heteronormative Attitudes and Beliefs Scale	10.1080/19419899.2013.876444

Reason for Exclusion/ Inclusion	Author(s)	Year	Title	DOI
Population	Hailemariam, T. G.; Tesfaye, T.; Melese, T.; Alemayehu, W.; Kenore, Y.; Lelamo, Y.; Saul, T.; Seifu, C. N.	2015	Sexual experiences and emergency contraceptive use among female university students: a cross-sectional study at Wachamo University, Ethiopia	10.1186/s13104-015-1070-7
Population	Jami, Humaira; Kamal, Anila	2015	Measuring attitudes toward Hijras in Pakistan: Gender and religiosity in perspective	N/A
Population	Kahler, C. W.; Wray, T. B.; Pantalone, D. W.; Mastroleo, N. R.; Kruis, R. D.; Mayer, K. H.; Monti, P. M.	2015	Assessing sexual motives for drinking alcohol among HIV-positive men who have sex with men	10.1037/adb0000006
Population	Kraus, Shane W.; Rosenberg, Harold; Tompsett, Carolyn J.	2015	Assessment of self-efficacy to employ self-initiated pornography use-reduction strategies	10.1016/j.addbeh.2014.09.012
Population	Moreno, Alexander; Herazo, Edwin; Oviedo, Heidi; Campo-Arias, Adalberto	2015	Measuring homonegativity: psychometric analysis of Herek's attitudes toward lesbians and gay men scale (ATLG) in Colombia, South America	10.1080/00918369.2014.1003014
Population	Páez, José; Hevia, Guillermo; Pesci, Florencia; Rabbia, Hugo H.	2015	Construcción y validación de una escala de actitudes negativas hacia personas trans = Construction and validation of a Negative Attitudes toward Trans People Scale	N/A
Population	Pitpitan, Eileen V.; Strathdee, Steffanie A.; Semple, Shirley J.; Wagner, Karla D.; Chavarin, Claudia V.; Earnshaw, Valerie A.; Patterson, Thomas L.	2015	Perceived Stigma of Purchasing Sex Among Latino and Non-Latino Male Clients of Female Sex Workers in Tijuana, Mexico	10.1007/s10903-013-9895-4
Population	Priest, Hannah Marie	2015	Development and validation of a theory of planned behavior-based instrument to predict Human Papillomavirus vaccination intentions of college males at a southeastern university	N/A
Population	Rehman, Khaleeq Ur; Asif Mahmood, Mohammad; Sheikh, Saba Shabbir; Sultan, Tipu; Khan, M. Amanullah	2015	The Female Sexual Function Index (FSFI): Translation, Validation, and Cross-Cultural Adaptation of an Urdu Version "FSFI-U"	10.1002/sm2.77
Population	Rogers, Darrin L.; Cervantes, Emanuel; Espinosa, Joanna C.	2015	Development and validation of the Belief in Female Sexual Deceptiveness Scale	10.1177/0886260514536282
Population	Schensul, Stephen L.; Singh, Rajendra; Schensul, Jean J.; Verma, Ravi K.; Burleson, Joseph A.; Nastasi, Bonnie K.	2015	Community gender norms change as a part of a multilevel approach to sexual health among married women in Mumbai, India	10.1007/s10464-015-9731-1
Population	Sligar, K.; Belfy, A.; Barnett, M.	2015	The relationship between disclosure of virginity status and dysfunctional sexual beliefs	N/A
Population	Söderberg, Malin; Christensson, Kyllike; Lundgren, Ingela; Hildingsson, Ingegerd	2015	Women's attitudes towards fertility and childbearing - A study based on a national sample of Swedish women validating the Attitudes to Fertility and Childbearing Scale (AFCS)	10.1016/j.srhc.2015.01.002
Population	Starosta, Amy J.; Berghoff, Christopher R.; Earleywine, Mitch	2015	Factor structure and gender stability in the Multidimensional Condom Attitudes Scale	10.1177/1073191114547887
Population	Talboys, Sharon Louise	2015	The public health impact of eve teasing: Public sexual harassment and its association with common mental disorders and suicide ideation among young women in rural Punjab, India	N/A
Population	Vaughn, Allison A.; Teeters, Stacy A.	2015	Development and psychometric properties of the Attitudes Towards Heterosexuals Scale (ATHS): A useful tool alone or in combination	10.1080/19419899.2014.1000949
Population	Woodford, Michael R.; Chonody, Jill M.; Kulick, Alex; Brennan, David J.; Renn, Kristen	2015	The LGBQ microaggressions on campus scale: A scale development and validation study	10.1080/00918369.2015.1078205
Population	Baldwin-White, Adrienne; Thompson, Marilyn S.; Gray, Amanda	2016	Pre- and postintervention factor analysis of the Illinois Rape Myth Acceptance Scale	10.1080/10926771.2015.1107173
Population	Bird, Yelena; Solis, Luis H.; Mbonu, Chinaedu Anulika	2016	Sexual attitudes, norms, condom use, and adherence of Hispanic and non-Hispanic undergraduate students: a cross-sectional study of three community colleges in southwestern US	10.2147/PPA.S108688
Population	Cunningham-Erves, Jennifer; Talbott, Laura L.; O'Neal, Marcia R.; Ivankova, Nataliya V.; Wallston, Kenneth A.	2016	Development of a Theory-based, Sociocultural Instrument to Assess Black Maternal Intentions to Vaccinate Their Daughters Aged 9 to 12 Against HPV	10.1007/s13187-015-0867-3

Reason for Exclusion/ Inclusion	Author(s)	Year	Title	DOI
Population	Cuturilo, Goran; Vucinic, Olivera Kontic; Novakovic, Ivana; Ignjatovic, Svetlana; Mijovic, Marija; Sulovic, Nenad; Vukolic, Dusan; Komnenic, Milica; Tadic, Jasmina; Cetkovic, Aleksandar; Belic, Aleksandra; Ljubic, Aleksandar	2016	Clients' perception of outcome of team-based prenatal and reproductive genetic counseling in Serbian service using the Perceived Personal Control (PPC) questionnaire	10.1007/s10897-015-9857-1
Population	Da Costa, Filipa Alves; Ribeiro, Manuel Castro; Braga, Sofia; Carvalho, Elisabete; Francisco, Fátima; Miranda, Ana Costa; Moreira, António; Fallowfield, Lesley	2016	Sexual Dysfunction in Breast Cancer Survivors: Cross-Cultural Adaptation of the Sexual Activity Questionnaire for Use in Portugal	10.20344/amp.7389
Population	Dargie, Emma; Holden, Ronald R.; Pukall, Caroline F.	2016	The Vulvar Pain Assessment Questionnaire inventory	10.1097/j.pain.0000000000000682
Population	Dyar, Christina; Feinstein, Brian A.; Eaton, Nicholas R.; London, Bonita	2016	Development and initial validation of the Sexual Minority Women Rejection Sensitivity Scale	10.1177/0361684315608843
Population	Ellis, Lee; Hoskin, Anthony W.; Ratnasingam, Malini	2016	Testosterone, risk taking, and religiosity: Evidence from two cultures	10.1111/jssr.12248
Population	Ellis, Lee; Hoskin, Anthony W.; Ratnasingam, Malini	2016	'Testosterone, Risk Taking, and Religiosity: Evidence from Two Cultures': Erratum	10.1111/jssr.12295
Population	Houston-Kolnik, Jaclyn D.; Todd, Nathan R.; Wilson, Midge	2016	Preliminary validation of the Sex Trafficking Attitudes Scale	10.1177/1077801215621178
Population	Jackson, Skyler D.; Mohr, Jonathan J.	2016	Conceptualizing the closet: Differentiating stigma concealment and nondisclosure processes	10.1037/sgd0000147
Population	Jawed-Wessel, Sofia; Herbenick, Debby; Schick, Vanessa; Fortenberry, J. Dennis; Cattelona, Georg'Ann; Reece, Michael	2016	Development and validation of the Maternal and Partner Sex During Pregnancy Scales	10.1080/0092623X.2015.1113587
Population	Khan, Tahir Mehmood; Buksh, Malik Allah; Rehman, Inayat Ur; Saleem, Ahsan	2016	Knowledge, attitudes, and perception towards human papillomavirus among university students in Pakistan	10.1016/j.pvr.2016.06.001
Population	Kraus, Shane W.; Rosenberg, Harold	2016	Lights, camera, condoms! Assessing college men's attitudes toward condom use in pornography	10.1080/07448481.2015.1085054
Population	Malmborg, Agota; Persson, Elin; Brynhildsen, Jan; Hammar, Mats	2016	Hormonal contraception and sexual desire: A questionnaire-based study of young Swedish women	10.3109/13625187.2015.1079609
Population	Neels, Hedwig; Wyndaele, Jean-Jacques; Tjalma, Wiebren A. A.; Wachter, Stefan de; Wyndaele, Michel; Vermandel, Alexandra	2016	Knowledge of the pelvic floor in nulliparous women	10.1589/jpts.28.1524
Population	Nguyen, Trang Quynh; Poteat, Tonia; Bandeen-Roche, Karen; German, Danielle; Nguyen, Yen Hai; Vu, Loan Kieu-Chau; Nguyen, Nam Thi-Thu; Knowlton, Amy R.	2016	The Internalized Homophobia Scale for Vietnamese Sexual Minority Women: Conceptualization, Factor Structure, Reliability, and Associations With Hypothesized Correlates	10.1007/s10508-016-0694-6
Population	Page, Thomas E.; Pina, Afroditi; Giner-Sorolla, Roger	2016	"It was only harmless banter!" The development and preliminary validation of the moral disengagement in sexual harassment scale	10.1002/ab.21621
Population	Patra, Shraboni; Arokiasamy Perianayagam; Goli, Srinivas	2016	Mother's health knowledge and its links with the illness and medical care of their children in India	10.1108/HE-06-2014-0069
Population	Salameh, P.; Zeenny, R.; Salamé, J.; Waked, M.; Barbour, B.; Zeidan, N.; Baldi, I.	2016	ATTITUDES TOWARDS AND PRACTICE OF SEXUALITY AMONG UNIVERSITY STUDENTS IN LEBANON	10.1017/S0021932015000139
Population	Sanchez, Delida; Whittaker, Tiffany A.; Hamilton, Emma; Zayas, Luis H.	2016	Perceived discrimination and sexual precursor behaviors in Mexican American preadolescent girls: The role of psychological distress, sexual attitudes, and marianismo beliefs	10.1037/cdp0000066
Population	Shokoohi, Mostafa; Karamouzian, Mohammad; Mirzazadeh, Ali; Haghdoost, AliAkbar; Rafierad, Ali-Ahmad; Sedaghat, Abbas; Sharifi, Hamid	2016	HIV Knowledge, Attitudes, and Practices of Young People in Iran: Findings of a National Population-Based Survey in 2013	10.1371/journal.pone.0161849
Population	Steinke, Elaine E.; Barnason, Susan; Mosack, Victoria; Hill, Twyla J.	2016	Baccalaureate nursing students' application of social-cognitive sexual counseling for cardiovascular patients: A web-based educational intervention	10.1016/j.nedt.2016.05.015
Population	Swann, Gregory; Minshew, Reese; Newcomb, Michael E.; Mustanski, Brian	2016	Validation of the Sexual Orientation Microaggression Inventory in Two Diverse Samples of LGBTQ Youth	10.1007/s10508-016-0718-2
Population	Zeng, Xianglong; Pan, Yiqin; Zhou, Han; Yu, Shi; Liu, Xiangping	2016	Exploring Different Patterns of Love Attitudes among Chinese College Students	10.1371/journal.pone.0166410

Reason for Exclusion/ Inclusion	Author(s)	Year	Title	DOI
Population	Zhao, Yanping; Wong, Carlos King Ho; Miu, Heidi Yin Hai; Yuen, Winnie Wing Yan; Chin, Weng Yee; Luo, Tongyong; Wong, William Chi Wai	2016	Translation and validation of a Condom Self-Efficacy Scale (CSES) Chinese version	10.1521/aeap.2016.28.6.499
Population	Ahuja, Kanika K.	2017	Development of Attitudes Toward Homosexuality Scale for Indians (AHSI)	10.1080/00918369.2017.1289006
Population	Alladio, Y.; Moran, V.; Olaz, F.	2017	Argentinean validation of the Rape Supportive Attitude Scale	10.18800/psico.201701.008
Population	Badenes-Ribera, Laura; Frias-Navarro, Dolores; Berrios-Riquelme, Jose; Longobardi, Claudio	2017	Italian validation of the Queer/Liberationist scale (short version) in a sample of university students: Confirmatory factor analysis	10.1007/s13178-016-0256-7
Population	Bendixen, Mons; Kennair, Leif Edward Ottesen	2017	When less is more: Psychometric properties of Norwegian short-forms of the Ambivalent Sexism Scales (ASI and AMI) and the Illinois Rape Myth Acceptance (IRMA) Scale	10.1111/sjop.12392
Population	Bidell, Markus P.	2017	The Lesbian, Gay, Bisexual, and Transgender Development of Clinical Skills Scale (LGBT-DOCSS): Establishing a new interdisciplinary self-assessment for health providers	10.1080/00918369.2017.1321389
Population	Crosby, R. A.; Sanders, S. A.; Graham, C. A.; Milhausen, R.; Yarber, W. L.; Mena, L.	2017	Evaluation of the Condom Barriers Scale for Young Black Men Who Have Sex With Men: reliability and Validity of 3 Subscales	10.1097/OLQ.0000000000000562
Population	DeBlaere, Cirleen; Brewster, Melanie E.	2017	A confirmation of the Drive for Muscularity Scale with sexual minority men	10.1037/sgd0000224
Population	DeBlaere, Cirleen; Chadwick, Caleb N.; Zelaya, David G.; Bowie, Jhodi-Ann; Bass, Melanie F.; Finzi-Smith, Zoeann	2017	The feminist identity composite: An examination of structural validity with sexual minority women	10.1177/0361684316676046
Population	Frias-Navarro, Dolores; Badenes-Ribera, Laura; Monterde-I-Bort, Hector	2017	Evidence of validity of the Beliefs about Children’s Adjustment in Same-Sex Families Scale	10.1007/s13178-016-0246-9
Population	Golub, Sarit A.; Gamarel, Kristi E.	2017	Psychometric evaluation of the Condom Barriers and Motivations Scale (CBMS)	10.1007/s10865-016-9815-x
Population	Górska, Paulina; Bilewicz, Michał; Winiewski, Mikołaj; Waszkiewicz, Agata	2017	On old-fashioned versus modern homonegativity distinction: Evidence from Poland	10.1080/00918369.2016.1179029
Population	Haynes, Meagan Campol; Ryan, Nessa; Saleh, Mona; Winkel, Abigail Ford; Ades, Veronica	2017	Contraceptive Knowledge Assessment: validity and reliability of a novel contraceptive research tool	10.1016/j.contraception.2016.09.002
Population	Hogben, Matthew; Harper, Christopher; Habel, Melissa A.; Brookmeyer, Kathryn; Friedman, Allison	2017	Attitudes to sexual health in the United States: results from a national survey of youth aged 15-25 years	10.1071/SH16164
Population	Horne, Sharon G.; Maroney, Meredith R.; Geiss, Meghan L.; Dunnavant, Bridget R.	2017	The reliability and validity of a Russian version of the Lesbian Internalized Homophobia Scale	10.11621/pir.2017.0201
Population	Jafari, Fatemeh; Rashidi, Samaneh	2017	Iranian women's knowledge and attitude regarding preconception health: 12 years after integration into the primary health care network	10.4103/JNMS.JNMS_14_17
Population	Johnson, S. M.; Murphy, M. J.; Gidycz, C. A.	2017	Reliability and Validity of the Sexual Experiences Survey-Short Forms Victimization and Perpetration	10.1891/0886-6708.VV-D-15-00110
Population	Kanamori, Yasuko; Cornelius-White, Jeffrey H. D.; Pegors, Teresa K.; Daniel, Todd; Hulgus, Joseph	2017	Development and validation of the Transgender Attitudes and Beliefs Scale	10.1007/s10508-016-0840-1
Population	Kujawa, Lindsay Frances	2017	Development and validation of the partners' approval of Nonsexual Extradyadic Behaviors Scale	N/A
Population	Lee, K. E.; Jung, S.-A.; Yoon, H.; Park, S. H.; Moon, C. M.; Kim, E. S.; Kim, S.-E.; Yang, S.-K.	2017	Factors associated with pregnancy-related knowledge in reproductive-aged women with inflammatory bowel disease	N/A
Population	McDermott, Ryon C.; Levant, Ronald F.; Hammer, Joseph H.; Hall, Rosalie J.; McKelvey, Daniel K.; Jones, Zachary	2017	Further examination of the factor structure of the Male Role Norms Inventory-Short Form (MRNI-SF): Measurement considerations for women, men of color, and gay men	10.1037/cou0000225
Population	Philippe, Frederick L.; Vallerand, Robert J.; Bernard-Desrosiers, Léa; Guilbault, Valérie; Rajotte, Guillaume	2017	Understanding the cognitive and motivational underpinnings of sexual passion from a dualistic model	10.1037/pspp0000116
Population	Puckett, Jae A.; Newcomb, Michael E.; Ryan, Daniel T.; Swann, Greg; Garofalo, Robert; Mustanski, Brian	2017	Internalized homophobia and perceived stigma: A validation study of stigma measures in a sample of young men who have sex with men	10.1007/s13178-016-0258-5
Population	Rhoads, Kelley	2017	Social media and perceptions of sexual consent: Development and psychometric assessment of two consent measures	N/A
Population	Rodriguez-Santero, J.; Munoz, MAGC; Galvez, A. M.P.	2017	LOVE ATTITUDES STYLES AMONGST COLLEGE STUDENTS. Differences by sex-gender	10.3989/ris.2017.75.3.15.171

Reason for Exclusion/ Inclusion	Author(s)	Year	Title	DOI
Population	Rominski, Sarah D.; Darteh, Eugene; Dickson, Kwamena Sekyi; Munro-Kramer, Michelle	2017	Attitudes toward abortion among students at the University of Cape Coast, Ghana	10.1016/j.srhc.2016.10.002
Population	Simbar, M.; Khalesi, Z. B.; Azin, S. A.	2017	The development and validation of Sexual Health Education Needs Assessment Questionnaire of Iranian engaged coup	10.22086/gmj.v6i4.854
Population	Thakur, Meghna Basu; Paul, Priscilla	2017	Sexual harassment in academic institutions: A conceptual review	N/A
Population	Xu, Wenjian; Zheng, Lijun; Xu, Yin; Zheng, Yong	2017	Internalized homophobia, mental health, sexual behaviors, and outness of gay/bisexual men from Southwest China	10.1186/s12939-017-0530-1
Population	Abou El-Ola, M. J.; Rajab, M. A.; Abdallah, D. I.; Fawaz, I. A.; Awad, L. S.; Tamim, H. M.; Ibrahim, A. O.; Mugharbil, A. M.; Moghnieh, R. A.	2018	Low rate of human papillomavirus vaccination among schoolgirls in Lebanon: Barriers to vaccination with a focus on mothers' knowledge about available vaccines	10.2147/TCRM.S152737
Population	Alavi-Arjas, F.; Farnam, F.; Granmayeh, M.; Haghani, H.	2018	The Effect of Sexual and Reproductive Health Education on Knowledge and Self-Efficacy of School Counselors	10.1016/j.jadohealth.2018.05.031
Population	Alharbi, Kholoud K.; Alkharan, Afnan A.; Abukhamseen, Doha A.; Altassan, Maryam A.; Alzahrani, Wareef; Fayed, Amel	2018	Knowledge, readiness, and myths about menstruation among students at the Princess Noura University	10.4103/jfmpc.jfmpc_279_18
Population	Ali, A. N.; Jie, J. S.; Prajapati, S. K.; Iqbal, M. Z.; Ahmed, N. Z.; Alshammari, T. M.	2018	A longitudinal KAP study on HPV immunised adolescents' in Malaysia	10.18311/jnr/2018/22249
Population	Areskoug-Josefsson, Kristina; Thidell, Fredrik; Rolander, Bo; Ramstrand, Nerrolyn	2018	Prosthetic and orthotic students' attitudes toward addressing sexual health in their future profession	10.1177/0309364618775444
Population	Costa, E.C.V.; McIntyre, T.; Ferreira, D.	2018	Safe-Sex Knowledge, Self-Assessed HIV Risk, and Sexual Behaviour of Young Portuguese Women	10.1159/000486466
Population	Czerwinski, Fabian; Finne, Emily; Alfes, Jana; Kolip, Petra	2018	Effectiveness of a school-based intervention to prevent child sexual abuse—Evaluation of the German IGEL program	10.1016/j.chiabu.2018.08.023
Population	Fino, Emanuele; Giuliani, Marta; Pierleoni, Luca; Gambino, Gaetano; Cosmi, Valentina; Simonelli, Chiara	2018	Factor Structure and Psychometric Properties of the Italian Version of the Homosexuality Scale of the Trueblood Sexual Attitudes Questionnaire	10.1080/00918369.2017.1364558
Population	Franc, Elisabeth; Khazaal, Yasser; Jasiowka, Katarzyna; Lepers, Thibault; Bianchi-Demicheli, Francesco; Rothen, Stéphane	2018	Factor structure of the Cybersex Motives Questionnaire	10.1556/2006.7.2018.67
Population	Galupo, M. Paz; Mitchell, Renae C.; Davis, Kyle S.	2018	Face validity ratings of sexual orientation scales by sexual minority adults: Effects of sexual orientation and gender identity	10.1007/s10508-017-1037-y
Population	Gholamfarkhani, S.; Khoori, E.; Derakhshanpour, F.; Aryaie, M.; Wurtele, S. K.	2018	PSYCHOMETRIC PROPERTIES OF THE PERSONAL SAFETY QUESTIONNAIRE AND "WHAT IF" SITUATIONS TEST: PERSIAN VERSIONS	10.24195/2414-4665-2018-1-2
Population	Hall, Kelli Stidham; Manu, Abubakar; Morhe, Emmanuel; Harris, Lisa H.; Loll, Dana; Ela, Elizabeth; Kolenic, Giselle; Dozier, Jessica L.; Challa, Sneha; Zochowski, Melissa K.; Boakye, Andrew; Adanu, Richard; Dalton, Vanessa K.	2018	Development and Validation of a Scale to Measure Adolescent Sexual and Reproductive Health Stigma: Results From Young Women in Ghana	10.1080/00224499.2017.1292493
Population	Hall, Kelli Stidham; Morhe, Emmanuel; Manu, Abubakar; Harris, Lisa H.; Ela, Elizabeth; Loll, Dana; Kolenic, Giselle; Dozier, Jessica L.; Challa, Sneha; Zochowski, Melissa K.; Boakye, Andrew; Adanu, Richard; Dalton, Vanessa K.	2018	Factors associated with sexual and reproductive health stigma among adolescent girls in Ghana	10.1371/journal.pone.0195163
Population	Holloway, Jacqueline L.; Pulido, Mary L.	2018	Sexual Abuse Prevention Concept Knowledge: Low Income Children Are Learning but Still Lagging	10.1080/10538712.2018.1496506
Population	Jalambadani, Z.; Garmaroudi, G.; Tavousi, M.	2018	Education Based on Theory of Planned Behavior over Sexual Function of Women with Breast Cancer in Iran	10.4103/apjon.apjon_67_17
Population	Leon-Larios, Fátima; Gómez-Baya, Diego	2018	Design and validation of a brief questionnaire to assess young´s sexual knowledge	N/A
Population	Levant, Ronald F.; McCurdy, Eric R.	2018	Toward diversifying research participants: Measurement invariance of the Male Role Norms Inventory–Short Form (MRNI-SF) across recruitment method	10.1037/men0000138

Reason for Exclusion/ Inclusion	Author(s)	Year	Title	DOI
Population	Magnus, Maria C.; Anderson, Emma L.; Howe, Laura D.; Joinson, Carol J.; Penton-Voak, Ian S.; Fraser, Abigail	2018	Childhood psychosocial adversity and female reproductive timing: A cohort study of the ALSPAC mothers	10.1136/jech-2017-209488
Population	Marcantonio, Tiffany L.; Jozkowski, Kristen N.; Lo, Wen-Juo	2018	Beyond 'just saying no': A preliminary evaluation of strategies college students use to refuse sexual activity	10.1007/s10508-017-1130-2
Population	McRae, Lamerial; Gonzalez, Jennifer E.; Dominguez, Vanessa; Daire, Andrew Patrick; Liu, Xun	2018	Factor Analysis and Construction of the Acceptance of Couple Violence Scale	10.1080/07481756.2017.1308225
Population	Nascimento, Bruna S.; Hanel, Paul P. H.; Monteiro, Renan P.; Gouveia, Valdiney V.; Little, Anthony C.	2018	Sociosexuality in Brazil: Validation of the SOI-R and its correlates with personality, self-perceived mate value, and ideal partner preferences	10.1016/j.paid.2017.12.007
Population	Nunes, Kevin L.; Hermann, Chantal A.; White, Kristen; Pettersen, Cathrine; Bumby, Kurt	2018	Attitude May Be Everything, But Is Everything an Attitude? Cognitive Distortions May Not Be Evaluations of Rape	10.1177/1079063215625489
Population	Ortiz, Amanda M.	2018	Developing a Measure of Purity Culture: Sexual messages in Evangelical Christian culture	N/A
Population	Perera, U.A.P.; Abeysena, C.	2018	Prevalence and associated factors of risky sexual behaviors among undergraduate students in state universities of Western Province in Sri Lanka: A descriptive cross sectional study	10.1186/s12978-018-0546-z
Population	Purewal, Satvinder; Chapman, Sarah; Czuber-Dochan, Wladyslawa; Selinger, Christian; Steed, Helen; Brookes, Matthew J.	2018	Systematic review: the consequences of psychosocial effects of inflammatory bowel disease on patients' reproductive health	10.1111/apt.15019
Population	Ross, M. W.; Leshabari, S.; Rosser, B. R. S.; Trent, M.; Mgopa, L.; Wadley, J.; Kohli, N.; Agardh, A.	2018	Evaluation of an assessment instrument for a sexual health curriculum for nurses and midwifery students in Tanzania: The sexual health education for professionals scale (SHEPS)	10.1016/j.apnr.2018.01.005
Population	Simons, Jack D.	2018	School Counselor Sexual Minority Advocacy Competence Scale (SCSMACS): Development, validity, and reliability	10.1037/t69044-000.
Population	Stone, Nicole; Graham, Cynthia; Anstee, Sydney; Brown, Katherine; Newby, Katie; Ingham, Roger	2018	Enhancing condom use experiences among young men to improve correct and consistent condom use: feasibility of a home-based intervention strategy (HIS-UK)	10.1186/s40814-018-0257-9
Population	Thongnopakun, S.; Pumpaibool, T.; Somrongthong, R.	2018	The association of sociodemographic characteristics and sexual risk behaviors with health literacy toward behaviors for preventing unintended pregnancy among university students	10.2147/JMDH.S156264
Population	Tran, Ha; Ross, Michael W.; Diamond, Pamela M.; Berg, Rigmor C.; Weatherburn, Peter; Schmidt, Axel J.	2018	Structural Validation and Multiple Group Assessment of the Short Internalized Homonegativity Scale in Homosexual and Bisexual Men in 38 European Countries: Results From the European MSM Internet Survey	10.1080/00224499.2017.1380158
Population	Tran, N.N.Y.; Vo, T. Q.	2018	Knowledge, perceptions, and attitudes toward contraceptive medicine among undergraduate students in Southern Vietnam	10.22377/ajp.v12i01.2350
Population	Tross, S.; Pinho, V.; Lima, J. E.; Ghiroli, M.; Elkington, K. S.; Strauss, D. H.; Wainberg, M. L.	2018	Participation in HIV Behavioral Research: Unanticipated Benefits and Burdens	10.1007/s10461-018-2114-5
Population	Wyman Battalen, Adeline	2018	Beliefs, perceptions, and socialization practices of lesbian, gay, and heterosexual adoptive parents	N/A
Population	Zsila, Ágnes; Pagliassotti, Dru; Urbón, Rábert; Orosz, Gábor; Király, Orsolya; Demetrovics, Zsolt	2018	Loving the love of boys: Motives for consuming yaoi media	10.1371/journal.pone.0198895
Population	Abel, Gene G.; Jordan, Alan; Harlow, Nora; Hsu, Yu-Sheng	2019	Preventing Child Sexual Abuse: Screening for Hidden Child Molesters Seeking Jobs in Organizations That Care for Children	10.1177/1079063218793634
Population	Ajayi, Anthony Idowu; Somefun, Oluwaseyi Dolapo	2019	Transactional sex among Nigerian university students: The role of family structure and family support	10.1371/journal.pone.0210349
Population	Areskoug-Josefsson, Kristina; Sjökvist, Michael; Bülow, Pia H.; Rolander, Bo	2019	Psychometrics of the students´ attitudes towards addressing sexual health scale for students in social work	10.1080/02615479.2019.1582619
Population	Bojan, Kelly; Westfall, Andrew O.; Fernandez, M. Isabel; Martinez, Jaime; Oyedele, Temitope; Wilson, Craig M.; Hosek, Sybil	2019	A Measure to Assess HIV Treatment Readiness among Adolescents and Young Adults	10.1080/17450128.2019.1595798
Population	Convertino, Alexandra D.; Gonzales, Manuel [4th.]; Malcarne, Vanessa L.; Blashill, Aaron J.	2019	A psychometric investigation of the Sociocultural Attitudes Towards Appearance Questionnaire-4-Revised among sexual minority adults in the U.S	10.1016/j.bodyim.2019.08.013

Reason for Exclusion/ Inclusion	Author(s)	Year	Title	DOI
Population	Dyar, Christina; Feinstein, Brian A.; Davila, Joanne	2019	Development and Validation of a Brief Version of the Anti-Bisexual Experiences Scale	10.1007/s10508-018-1157-z
Population	Evcili, Funda	2019	Sexual Orientation Myhts Scale (SOMS): Development, validity and reliability in Turkey	10.1111/ppc.12371
Population	Gangos, Christina Jane; Nega, Chrysanthi; Fotini-Sonia Apergi	2019	Adaptation and Psychometric Evaluation of the Children's Knowledge of Abuse Questionnaire (CKAQ-RIII) in Greek Elementary School Children	10.1080/10538712.2018.1538175
Population	Gil-Llario, M. D.; Ruiz-Palomino, E.; Morell-Mengual, V.; Giménez-García, C.; Ballester-Arnal, R.	2019	Validation of the AIDS Prevention Questionnaire: A Brief Self-Report Instrument to Assess Risk of HIV Infection and Guide Behavioral Change	10.1007/s10461-018-2224-0
Population	Ingram, Lucy A.; Macauda, Mark; Lauckner, Carolyn; Robillard, Alyssa	2019	Sexual Behaviors, Mobile Technology Use, and Sexting Among College Students in the American South	10.1177/0890117118779008
Population	Kiss, Mark J.; Morrison, Todd G.; McDonagh, Lorraine K.	2019	Male Body Image Self-Consciousness during physical intimacy (M-BISC): Validating the M-BISC with gay men	10.1016/j.jsxm.2018.12.004
Population	Martinez, Larry R.; Smith, Nicholas A.	2019	Development of a scale to measure heterosexual identity	10.1037/sgd0000313
Population	Motedayen, Mahsa; Kalantarkousheh, Seyed Mohammad; Scheier, Lawrence M.; Komarc, Martin	2019	Psychometric validation of the Sexual Knowledge and Attitudes Test –Adolescents (SKAT-A) in an Iranian sample	10.1080/23311908.2019.1585505
Population	Nadal, Kevin L.	2019	Measuring LGBTQ microaggressions: The Sexual Orientation Microaggressions Scale (SOMS) and the Gender Identity Microaggressions Scale (GIMS)	10.1080/00918369.2018.1542206
Population	Najmabadi, K. M.; Karimi, F. Z.; Roudsari, R. L.; Abdollahi, M.; Zarifnejad, G.	2019	Evaluation of validity and reliability of Persian version of the gender-equitable men-scale (GEM-scale) in male students, Iran	10.7860/JCDR/2019/34280.12523
Population	Oliveira, Renato de; Montagna, Erik; Zaia, Victor; Torres, Bayardo Baptista; Barbosa, Caio Parente	2019	The Development of Cognitive and Affective Skills Through a Sexual and Reproductive Health Medical Education Unit	10.1016/j.esxm.2019.06.008
Population	Ordonez, E. F.M.; Hernandez, V. V.M.	2019	ADAPTATION AND VALIDATION OF THE SEXUAL MYTHS SCALE IN SPANISH COLLAGE STUDENTS	N/A
Population	Pablo, Vallejo-Medina; Carlos Eduardo, Ramírez; Alejandro, Saavedra-Roa Diego; Mayra, Gómez-Lugo; Claudia, Pérez-Durán	2019	Spanish validation of female condom attitude scale and female condom use in Colombian young women	10.1186/s12905-019-0825-z
Population	Raju, Dheeraj; Beck, Lisa; Azuero, Andres; Azuero, Casey; Vance, David; Allen, Rebecca	2019	A Comprehensive Psychometric Examination of the Lesbian, Gay, and Bisexual Knowledge and Attitudes Scale for Heterosexuals (LGB-KASH)	10.1080/00918369.2018.1491705
Population	Roth, Alexis; Felsher, Marisa; Tran, Nguyen; Bellamy, Scarlett; Martinez-Donate, Ana; Krakower, Douglas; Szep, Zsofia	2019	Drawing from the Theory of Planned Behaviour to examine pre-exposure prophylaxis uptake intentions among heterosexuals in high HIV prevalence neighbourhoods in Philadelphia, Pennsylvania, USA: an observational study	10.1071/SH18081
Population	Sanz-Martos, Sebastian; López-Medina, Isabel M.; Álvarez-García, Cristina; Álvarez-Nieto, Carmen	2019	Sexuality and contraceptive knowledge in university students: instrument development and psychometric analysis using item response theory	10.1186/s12978-019-0791-9
Population	Satari, E.; Akbari Kamrani, M.; Farid, M.	2019	Necessity for redesigning premarital counseling classes based on marriage readiness from the perspective of adolescents and specialists: A need assessment based on the Bourich model and quadrant analysis	10.1515/ijamh-2019-0042
Population	Walsh, Kate; Honickman, Sara; Valdespino-Hayden, Zerbrina; Lowe, Sarah R.	2019	Dual Measures of Sexual Consent: A Confirmatory Factor Analysis of the Internal Consent Scale and External Consent Scale	10.1080/00224499.2019.1581882
Population	Wyman Battalen, Adeline; Dow-Fleisner, Sarah J.; Brodzinsky, David M.; McRoy, Ruth G.	2019	Lesbian, Gay, and Heterosexual Adoptive Parents' Attitudes Towards Racial Socialization Practices	10.1080/23761407.2019.1576565
Population	Wyman Battalen, Adeline; Farr, Rachel H.; Brodzinsky, David M.; McRoy, Ruth G.	2019	Socializing children about family structure: Perspectives of lesbian and gay adoptive parents	10.1080/1550428X.2018.1465875
Population	Baker, Nicholas A.; W Kim Halford	2020	Assessment of Couple Relationships Standards in Same-Sex Attracted Adults	10.1111/famp.12447
Population	Bekele, Delayehu; Surur, Feiruz; Nigatu, Balkachew; Teklu, Alula; Getinet, Tewodros; Kassa, Munir; Gebremedhin, Merhawi; Gebremichael, Berhe; Abesha, Yonas	2020	Knowledge and Attitude Towards Family Planning Among Women of Reproductive Age in Emerging Regions of Ethiopia	10.2147/JMDH.S277896
Population	Bismpas, Lazaros; Athanasiadis, Loukas; Papathanasiou, Nestor; Papadopoulos, Dimitrios; Konsta, Anastasia; Diakogiannis, Ioannis	2020	Psychopathology, psychosocial factors and sexuality of incarcerated sexual offenders in Greek prison	10.1016/j.jflm.2020.102031

Reason for Exclusion/ Inclusion	Author(s)	Year	Title	DOI
Population	Brasini, Maurizio; Tanzilli, Annalisa; Pistella, Jessica; Gentile, Daniela; Di Marco, Ivan; Mancini, Francesco; Lingiardi, Vittorio; Baiocco, Roberto	2020	The Social Mentalities Scale: A new measure for assessing the interpersonal motivations underlying social relationships	10.1016/j.paid.2020.110236
Population	Craig, Shelley L.; Iacono, Gio; Austin, Ashley; Eaton, Andrew D.; Pang, Nelson; Leung, Vivian W. Y.; Frey, Cressida J.	2020	The role of facilitator training in intervention delivery: Preparing clinicians to deliver affirmative group cognitive behavioral therapy to sexual and gender minority youth	10.1080/10538720.2020.1836704
Population	Do, M.; Hutchinson, P.; Omoluabi, E.; Akinyemi, A.; Akano, B.	2020	Partner Discussion as a Mediator of the Effects of Mass Media Exposure to FP on Contraceptive Use among Young Nigerians: Evidence from 3 Urban Cities	10.1080/10810730.2020.1716279
Population	Gebeyehu, Natnael Atnafu; Chanko, Kebreab Paulos; Yesigat, Yibeltal Mesfin	2020	Factors Associated with Condom Use Self-Efficacy Among Preparatory School Students in Sodo Town, Southern Ethiopia 2020: A Cross-Sectional Study	10.2147/HIV.S256683
Population	Giroux, Stacey A.; Gesselman, Amanda N.; Garcia, Justin R.; Luetke, Maya; Rosenberg, Molly	2020	The magnitude and potential impact of missing data in a sexual violence campus climate survey	10.1080/07448481.2019.1577865
Population	Hashemi, Maryam; Kohan, Shahnaz; Abdishahshahani, Mahshid	2020	Reproductive health self-care for female students: Educational needs assessment, Isfahan University of Medical Sciences 2018	10.4103/jehp.jehp_378_19
Population	Jain, S.; Zulfeen, M.; Goyal, S.; Sethi, N.; Pandey, D.	2020	Reproductive health care information seeking behaviour among educated Indian youth- A cross-sectional analysis of an Indian university	10.7860/JCDR/2020/43864.13833
Population	Leavitt, Chelom E.; Allsop, David B.; Busby, Dean M.; Driggs, Shayla M.; Johnson, Heather M.; Saxey, Matthew T.	2020	Associations of mindfulness with adolescent outcomes and sexuality	10.1016/j.adolescence.2020.04.008
Population	Lippa, Richard A.	2020	Interest, personality, and sexual traits that distinguish heterosexual, bisexual, and homosexual individuals: Are there two dimensions that underlie variations in sexual orientation?	10.1007/s10508-020-01643-9
Population	Lourenço, Danilo B.; Amaral, Breno Santos; Alfer-Junior, Wladimir; Vasconcellos, Ana; Russo, Fernanda; Sanchez-Salas, Rafael; Bianco, Bianca; Wagner, Andrew A.; Chang, Peter; Moschovas, Marcio Covas; Lemos, Gustavo Caserta; Carneiro, Arie	2020	Portuguese version of the Expanded Prostate Cancer Index Composite for Clinical Practice (EPIC-CP): psychometric validation and prospective application for early functional outcomes at a single institution	10.1186/s12894-020-00734-y
Population	Maletsky, Lisa D.	2020	Gender role strain, hookup culture, and sexual violence	N/A
Population	Marí-Ytarte, Rosa; Moreno-López, Roberto; Barranco-Barroso, Rut	2020	Sex and Relationship Education for the Autonomy and Emotional Well-Being of Young People	10.3389/fpsyg.2020.01280
Population	Moseson, Heidi; Lunn, Mitchell R.; Katz, Anna; Fix, Laura; Durden, Mary; Stoeffler, Ari; Hastings, Jen; Cudlitz, Lyndon; Goldberg, Eli; Lesser-Lee, Bori; Letcher, Laz; Reyes, Aneidys; Flentje, Annesa; Capriotti, Matthew R.; Lubensky, Micah E.; Obedin-Maliver, Juno	2020	Development of an affirming and customizable electronic survey of sexual and reproductive health experiences for transgender and gender nonbinary people	10.1371/journal.pone.0232154
Population	Mullens, Amy B.; Fein, Erich C.; Young, Ross McD; Dunne, Michael P.; Norton, Graham; Daken, Kirstie	2020	Stimulant expectancy questionnaire for men who have sex with men: A measure of substance-related beliefs	10.1002/hpja.271
Population	Pebdani, R. N.; Saeki, E.	2020	Validation of the Knowledge, Comfort, Approach, and Attitudes Towards Sexuality Scale for Use with Rehabilitation Counseling Students: An Exploratory Factor Analysis	10.1007/s11195-019-09611-5
Population	Pickett, Andrew C.; Valdez, Danny; Barry, Adam E.	2020	Measurement implications associated with refinement of sexual and gender identity survey items: A case study of the National College Health Assessment	10.1080/07448481.2019.1598421
Population	Preuß, Sabine; Ottenstein, Charlotte; Kachel, Sven; Steffens, Melanie C.	2020	Using Scenarios for Measuring the Affective and Behavioral Components of Attitudes Toward Lesbians and Gay Men: Validation of the SABA Scale	10.1007/s10508-020-01653-7
Population	Rahman, Azriani Ab; Ibrahim, Mohd Ismail; Rahman, Razlina Abdul; Arifin, Wan Nor; Ahmad, Mokhtarrudin	2020	Development and Validation of a Malay Version of the Questionnaire on Pornography Attitudes and Exposure for Youth in Kelantan	10.21315/mjms2020.27.2.14
Population	Rueda, Ma Mar; Cobo, Beatriz; López-Torrecillas, Francisca	2020	Measuring inappropriate sexual behavior among university students: Using the randomized response technique to enhance self-reporting	10.1177/1079063219825872
Population	Russell, Douglas; Higgins, Daryl	2020	Safeguarding Capabilities in Preventing Child Sexual Abuse: Exploratory Factor Analysis of a Scale Measuring Safeguarding Capabilities in Youth-Serving Organizations Workers	10.1177/1077559519870253

Reason for Exclusion/ Inclusion	Author(s)	Year	Title	DOI
Population	Salehi Moghaddam, F.; TorkZahrani, S.; Moslemi, A.; Azin, S. A.; Ozgoli, G.; Joulaee Rad, N.	2020	Effectiveness of Sexual Skills Training Program on Promoting Sexual Intimacy and Satisfaction in Women in Tehran (Iran): a randomized clinical Trial Study	10.22037/uj.v0i0.4690
Population	Schudson, Zachary C.	2020	Gender/sex diversity beliefs: Heterogeneity, links to prejudice, and diversity-affirming interventions	N/A
Population	Schudson, Zachary C.	2020	Gender/Sex Diversity Beliefs: Heterogeneity, Links to Prejudice, and Diversity-Affirming Interventions	N/A
Population	Smith, Erin R.; Perrin, Paul B.; Sutter, Megan E.	2020	Factor analysis of the Heterosexist Harassment, Rejection, and Discrimination Scale in lesbian, gay, bisexual, transgender, and queer people of colour	10.1002/ijop.12585
Population	Tesfaye, Y.; Agenagnew, L.	2020	Knowledge, Attitude, and Practices of Jimma Teacher Training College Students Toward Risky Sexual Behaviors, Jimma, Ethiopia	10.1016/j.esxm.2020.04.006
Population	van Ham, Kirsten; Brilleslijper-Kater, Sonja; van der Lee, Hanneke; van Rijn, Rick; van Goudoever, Hans; Teeuw, Rian	2020	Validation of the Sexual Knowledge Picture Instrument as a diagnostic instrument for child sexual abuse: study protocol	10.1136/bmjpo-2020-000799
Population	Yeater, Elizabeth A.; Leiting, Kari A.; Witkiewitz, Katie	2020	Assessing college women's perception of putative risk for being sexually victimized by a man: Development of the Sexual Assault Script Scale (SASS)	10.1007/s11199-019-01081-5
Population	Algarin, Angel B.; Hee Shrader, Cho; Hackworth, Benjamin T.; Varas-Diaz, Nelson; Fennie, Kristopher P.; Sheehan, Diana M.; Ibañez, Gladys E.	2021	Development and Validation of the Community PrEP-Related Stigma Scale (Community-PSS)	10.1521/aeap.2021.33.2.120
Population	Al-Saadi, Aisha N.; Al-Muqbali, Aisha H.; Dawi, Eihab	2021	Women's Knowledge of Cervical Cancer: A cross-sectional study in Al Buraimi Governorate, Oman	10.18295/squmj.4.2021.022
Population	Andrzejewski, J.; Dunville, R.; Johns, M. M.; Michaels, S.; Reisner, S. L.	2021	Medical Gender Affirmation and HIV and Sexually Transmitted Disease Prevention in Transgender Youth: Results from the Survey of Today's Adolescent Relationships and Transitions, 2018	10.1089/lgbt.2020.0367
Population	Bőthe, Beáta; Tóth-Király, István; Bella, Nóra; Potenza, Marc N.; Demetrovics, Zsolt; Orosz, Gábor	2021	Why do people watch pornography? The motivational basis of pornography use	10.1037/adb0000603
Population	Byrne, Christina A.; Petri, Jessica M.; Oh, Jin K.	2021	Changes in female rape myth acceptance among college students: A 20-year perspective	10.1007/s11199-021-01231-8
Population	Dina, G. D.; Debelo, B. T.; Belema, D.; Danusa, K. T.; Muleta, R.	2021	Fertility desire, knowledge of prevention of MCT of HIV and associated factors among men and women attending ART clinic at public health institutions of west shoa zone, oromia, Ethiopia, 2020	10.2147/HIV.S328565
Population	Esplin, Charlotte R.; Gabe, Hatch S.; Dorian, Hatch H.; Deichman, Conner L.; Braithwaite, Scott R.	2021	What Motives Drive Pornography Use?	10.1177/1066480720956640
Population	Ferrer-Perez, V. A.; Delgado-Alvarez, C.; Sánchez-Prada, A.; Bosch-Fiol, E.; Ferreiro-Basurto, V.	2021	Street sexual harassment: Experiences and attitudes among young spanish people	10.3390/ijerph181910375
Population	Grova, Monica M.; Donohue, Sean J.; Bahnson, Matthew; Meyers, Michael O.; Bahnson, Edward M.	2021	Allyship in Surgical Residents: Evidence for LGBTQ Competency Training in Surgical Education	10.1016/j.jss.2020.11.072
Population	Habte, Aklilu; Dessu, Samuel; Haile, Dereje	2021	Determinants of practice of preconception care among women of reproductive age group in southern Ethiopia, 2020: content analysis	10.1186/s12978-021-01154-3
Population	Hanna-Walker, Veronica; Busby, Dean M.; Leavitt, Chelom E.; James, Spencer L.	2021	Missing Piece of the Puzzle: Evaluating a General Meaning of Sex Measure	10.1111/famp.12631
Population	Herrero-Villoria, Cristina; Picornell-Lucas, Antonia; Patino-Alonso, Carmen	2021	Cultural Adaptation and Validation into Spanish of the Scale to Measure Attitudes Towards the Sex Trafficking of Women and Girls in Students of the University of Salamanca	10.1177/10778012211038971
Population	Jackman, Kevon-Mark P.; Hightow-Weidman, Lisa; Poteat, Tonia; Wirtz, Andrea L.; Kane, Jeremy C.; Baral, Stefan D.	2021	Evaluating psychometric determinants of willingness to adopt sexual health patient portal services among black college students: A mixed-methods approach	10.1080/07448481.2019.1660352
Population	Jackman, Kevon-Mark P.; Kane, Jeremy; Kharrazi, Hadi; Johnson, Renee M.; Latkin, Carl	2021	Using the Patient Portal Sexual Health Instrument in Surveys and Patient Questionnaires Among Sexual Minority Men in the United States: Cross-sectional Psychometric Validation Study	10.2196/18750
Population	Johnson, Nicole L.; Johnson, Dawn M.	2021	An Empirical Exploration Into the Measurement of Rape Culture	10.1177/0886260517732347

Reason for Exclusion/ Inclusion	Author(s)	Year	Title	DOI
Population	Kamalikhah, T.; Akbari, M.; Parhode, M.; Sabzmakan, L.; Khosrorad, R.; Mehri, A.	2021	Assessment of the relationship between demographic variables and knowledge, attitude and practice of Azad Islamic University students about sexually transmitted diseases in 2018	10.5114/hivar.2021.109558
Population	Karimi, L.; Rahmati, F.; Parandeh, A.	2021	Development and validation of psychometric properties of a questionnaire for sexual health literacy related to HIV/AIDS and sexually transmitted diseases among Iranian young men	10.5114/hivar.2021.105106
Population	Kashefi, Fatemeh; Bakhtiari, Afsaneh; Pasha, Hajar; Amiri, Fatemeh Nasiri; Bakouei, Fatemeh	2021	Student Attitudes About Reproductive Health in Public Universities: A Cross-Sectional Study	10.1177/0272684X20916599
Population	Leitch, Judith; Gandy-Guedes, Megan; Messinger, Lori	2021	The Psychometric Properties of the Competency Assessment Tool for Lesbian, Gay, Bisexual, and Transgender Clients	10.1080/00918369.2020.1712138
Population	Lopez-Saez, M. A.; Garcia-Dauder, D.; Montero, I.; Lecuona, O.	2021	Adaptation and validation of the LGBQ Ally Identity Measure (Adaptacion y validacion de la Medida de Identificacion Aliada LGBQ)	10.1080/02109395.2021.1989888
Population	López-Sáez, Miguel Ángel; García-Dauder, Dau; Montero, Ignacio; Lecuona, Óscar	2021	Adaptation and Validation of the Evasive Attitudes of Sexual Orientation Scale into Spanish	10.1080/00918369.2021.1898803
Population	Mesko, N.; Szatmari, D.; Lang, A.; Meston, C. M.; Buss, D. M.	2021	Why Hungarians Have Sex (YSEX?-HSF)	10.1007/s10508-021-02072-y
Population	Nunez, J. G.	2021	DESIGN AND VALIDATION OF CONTENT OF LITERACY SCALE IN SEXUAL AND REPRODUCTIVE HEALTH	10.31052/1853.1180.v26.n2.34244
Population	Padovese, V.; Farrugia, A.; Almabrok Ali Ghath, S.; Rossoni, I.	2021	Sexually transmitted infections’ epidemiology and knowledge, attitude and practice survey in a set of migrants attending the sexual health clinic in Malta	10.1111/jdv.16949
Population	Sanchez, B. P.; Concha-Salgado, A.; Fernandez-Suarez, A.; Juarros-Basterretxea, J.; Rodriguez-Diaz, F. J.	2021	The Gender Role Attitude Scale (GRAS) as an alternative for the crisis in the measurement of attitudes towards gender roles in Latin America: A study in Chilean university students	10.6018/analesps.438431
Population	Sang, Jordan M.; Egan, James E.; Meanley, Steven P.; Hawk, Mary E.; Markovic, Nina; Bear, Todd M.; Matthews, Derrick D.; Bauermeister, José A.	2021	Expectations and beliefs: How single young gay, bisexual and other men who have sex with men envision romantic relationships	10.1002/jcop.22522
Population	Siyez, D. M.; Ercan, G. S.; Esen, E.; Soylu, Y.; Baran, B.; Firuzan, E.; Belkis, O.; Sezer, T. D.; Guney, M. E.; Ergonen, A. T.; Gunay, T.; Tezcan, S.; Ozturk, B.	2021	Attitudes of the Turkish University Students and Staff Toward Sexual Harassment and Assault: Preliminary Psychometric Evaluation	10.1177/21582440211050374
Population	Suto, Maiko; Mitsunaga, Haruhiko; Honda, Yuka; Maeda, Eri; Ota, Erika; Arata, Naoko	2021	Development of a health literacy scale for preconception care: a study of the reproductive age population in Japan	10.1186/s12889-021-12081-0
Population	Torres, T. S.; Luz, P. M.; Bezerra, D. R.B.; Almeida-Brasil, C. C.; Marins, L. M.S.; Veloso, V. G.; Grinsztejn, B.; Harel, D.; Thombs, B. D.	2021	Translation and validation in Brazilian Portuguese of the reactions to homosexuality scale	10.1080/13548506.2021.1936580
Population	Wade, Ryan M.; Harper, Gary W.	2021	Toward a multidimensional construct of racialized sexual discrimination (RSD): Implications for scale development	10.1037/sgd0000443
Population	Wei, Ying; Chen, Qingsong	2021	Development of a Chinese College Students' Attitudes Toward Sexual Swear Words Scale	10.3389/fpsyg.2021.664065
Population	Wesson, Paul D.; Lippman, Sheri A.; Neilands, Torsten B.; Ahern, Jennifer; Kahn, Kathleen; Pettifor, Audrey	2021	Evaluating the Validity and Reliability of the Gender Equitable Men's Scale Using a Longitudinal Cohort of Adolescent Girls and Young Women in South Africa	10.1007/s10461-021-03436-0
Population	Wickham, Robert E.; Gutierrez, Renee; Giordano, Brenna L.; Rostosky, Sharon S.; Riggle, Ellen D. B.	2021	Gender and Generational Differences in the Internalized Homophobia Questionnaire: An Alignment IRT Analysis	10.1177/1073191119893010
Population	Xia, Xiaoyang	2021	An alternative measure for attitudes toward bisexual people	10.1037/sgd0000471
Population	Zimmermann, H.M.L.; van Bilsen, W.P.H.; Boyd, A.; Prins, M.; van Harreveld, F.; Davidovich, U.	2021	Prevention challenges with current perceptions of HIV burden among HIV-negative and never-tested men who have sex with men in the Netherlands: a mixed-methods study	10.1002/jia2.25715
Population	Baiocco, Roberto; Antoniucci, Chiara; Basili, Emanuele; Pistella, Jessica; Favini, Ainzara; Martin, Carol; Pastorelli, Concetta	2022	Perceived similarity to Gender Groups Scale: Validation in a sample of Italian LGB+ and heterosexual young adults	10.1007/s13178-021-00631-5
Population	Bergenfeld, Irina; Sales, Jessica M.; Minh, Tran Hung; Yount, Kathryn M.	2022	Measuring sexual communication in adolescent dating relationships in vietnam: Development and validation of the sexual communications scales for attitudes, self-efficacy, and behavior	10.1080/10510974.2022.2094981

Reason for Exclusion/ Inclusion	Author(s)	Year	Title	DOI
Population	Ferreira, Rayanne C.; Torres, Thiago S.; Ceccato, Maria Das Graças B; Bezerra, Daniel Rb; Thombs, Brett D.; Luz, Paula M.; Harel, Daphna	2022	Development and Evaluation of Short-Form Measures of the HIV/AIDS Knowledge Assessment Tool Among Sexual and Gender Minorities in Brazil: Cross-sectional Study	10.2196/30676
Population	Fradelos, Evangelos C.; Montegrico, James; Cornelius, Judith; Bakalis, Vissarion; Malliarou, Maria; Papathanasiou, Ioanna V.; Fasoi, Georgia; Kelesi, Martha; Kaba, Evridiki; Alikari, Victoria	2022	Translation and Validation of Nursing Students' Knowledge and Attitudes of Lesbian, Gay, Bisexual, Transgender Health Concerns Survey in the Greek Language	10.3390/healthcare10122547
Population	Gradellini, Cinzia; Kaleci, Shaniko; Sim-Sim, Margarida; Dias, Hélia; Mecugni, Daniela; Aaberg, Vicki; Gómez-Cantarino, Sagrario	2022	Adaptation and Validation of the Sexuality Attitudes and Beliefs Scale for the Italian Context	10.3390/ijerph192114162
Population	Habets, Petra; Jeandarme, Inge; Luk Gijs; Dombert, Beate; Mokros, Andreas	2022	Measuring sexual interest in persons who have sexually offended against children: investigating the Choice Reaction Time task using the Virtual People Set	10.1080/13552600.2021.1896808
Population	Kalichman, Seth C.; Shkembi, Bruno; Eaton, Lisa A.	2022	A novel psychometric approach to assessing intersectional hiv stigma: The geometric intersectional stigma scales	10.1007/s10865-022-00331-4
Population	Krivoshchekov, Vladislav; Gulevich, Olga; Ostroverkhova, Mariia	2022	The Conformity to Masculine Norms Inventory-30: Validity and measurement invariance of a Russian-language version	10.1037/men0000379
Population	Lee, Jungmin; Montegrico, Jhordin James C.	2022	Psychometric Evaluation of a Korean Version of the Sexual Risk Behavior Beliefs and Self-Efficacy Scale Among Female College Students: A Secondary Analysis	10.2147/PRBM.S387296
Population	Matsuzaka, Sara; Jamison, Laura; Avery, Lanice R.; Schmidt, Karen M.; Stanton, Alexis G.; Debnam, Katrina	2022	Gendered Racial Microaggressions Scale: Measurement invariance across sexual orientation	10.1177/03616843221118339
Population	Moussaoui, L. S.; Law, E.; Claxton, N.; Itaemaeki, S.; Siogope, A.; Virtanen, H.; Desrichard, O.; Consortium Sierra Leone Red Cross Soc	2022	Sexual and Reproductive Health: How Can Situational Judgment Tests Help Assess the Norm and Identify Target Groups? A Field Study in Sierra Leone	10.3389/fpsyg.2022.866551
Population	Ross, Jody M.; Machette, Anthony T.; Gonzalez, Reagan	2022	Testing the reliability of sexual aggression self-reports	10.1080/13552600.2022.2100933
Population	Canan, Sasha N.; Cozzolino, Lauren; Myers, Jaime L.; Jozkowski, Kristen N.	2023	Does gender inclusive language affect psychometric properties of the Illinois Rape Myth Acceptance Scale-Short Form? A two-sample validation study	10.1177/08862605221106144
Publication Type (Other)	Murray-Swank, Nichole A.; Pargament, Kenneth I.; Mahoney, Annette	2005	At the Crossroads of Sexuality and Spirituality: The Sanctification of Sex by College Students	
Publication Type (Other)	Ang, A.; Hu, C.	2006	Validating the Results of a Web-Based Behavioral Intervention	N/A
Publication Type (Other)	Goodson, Patricia; Buhi, Eric R.	2007	Behavioral skills and adolescent sexual risk behavior: The authors reply	10.1016/j.jadohealth.2007.05.014
Publication Type (Other)	Dunsmore, Sarah Catherine	2008	Why abstain from sex? Building and psychometric testing of the Sexual Abstinence Motivation Scale (SAMS)	N/A
Publication Type (Other)	Epstein, Marina	2008	Adolescents in conflict: Associations between gender socialization, gender conflict, and well-being	N/A
Publication Type (Other)	Arrington-Sanders, R.; Ellen, J.; Leonard, L.	2009	The impact of HIV self-efficacy on attitudes toward routine HIV testing among sexually active adolescents	10.1016/j.jadohealth.2008.10.074
Publication Type (Other)	Wolf, S. T.; Cooper, M. L.	2009	Validating an alcohol-intimacy expectancy scale	10.1111/j.1530-0277.2009.00957.x
Publication Type (Other)	Aleena, W.	2010	"Missed-conceptions": An intervention to increase knowledge of fertility and the effectiveness of IVF	10.1080/02646838.2010.519880
Publication Type (Other)	Brown, K.; Bayley, J.; Wallace, L.	2010	Towards improvement of planned and emergency contraceptive use in adolescents: Findings from a questionnaire study // The European Journal of Contraception & Reproductive Health Care	10.3109/13625181003733194
Publication Type (Other)	Case, S.; Williams, R.; Hensel, D.; Fortenberry, J. D.	2010	Family, friend, and sex partner influences on adolescent women's attitudes toward use of depot medroxyprogesterone	10.1016/j.jadohealth.2009.11.138
Publication Type (Other)	Elena, C.; Stefano, S.; Maurizio, S.; Mara, Z.; Gabriele, S.; Silvia, M.; Pierfrancesco, B.	2010	Is symptomatic vaginal prolapse related to descending severity and/or any vaginal compartment?	10.1002/nau.20930

Reason for Exclusion/ Inclusion	Author(s)	Year	Title	DOI
Publication Type (Other)	Gordon, Brian Cordell	2010	Development of a theory based instrument to predict the influence of sexually-oriented music lyrics on the sexual attitudes, beliefs, and intentions of African-American adolescents	N/A
Publication Type (Other)	Kumar, A.	2010	HIV/aids knowledge, attitudes and behaviors assessment of uttar pradesh students: A questionnaire study	10.1016/j.jomh.2010.09.010
Publication Type (Other)	Minnick, Dorlisa J.	2010	The factors influencing the sexual practices of adolescents in three African nations	N/A
Publication Type (Other)	Obasi, E. M.; Brooks, J. J.; Richards, J. L.; Ingram, J.	2010	Implicit alcohol-related cognitions: Testing the convergent validity of the Go/No-go association task and the implicit association test	10.1111/j.1530-0277.2010.01210.x
Publication Type (Other)	Oscos-Sanchez, M. A.; Lesser, J.; Berndt, A.; Pineda, D.; Oscos-Flores, L. D.; Tran, A.; Tinitigan, M.	2010	El Joven Noble concepts are correlated with violence-related attitudes and behaviors	10.1016/j.jadohealth.2009.11.150
Publication Type (Other)	Roos, A.; Thakar, R.; Sultan, A. H.	2010	Sexual problems in the gynaecology clinic: Are we making a mountain out of a molehill?	10.1002/nau.20973
Publication Type (Other)	Roos, A.; Thakar, R.; Sultan, A. H.	2010	Sexual problems in the gynaecology clinic: Are we making a mountain out of a molehill?	10.1007/s00192-010-1192-3
Publication Type (Other)	Stupiansky, N.; Hensel, D.; Fortenberry, J. D.	2010	Association between young womens perceptions of sexual partners and subsequent pregnancy or STI diagnosis	10.1016/j.jadohealth.2009.11.018
Publication Type (Other)	Teixeira, F.	2010	Sexuality, gender and media	N/A
Publication Type (Other)	Trent, M.; Chung, S.; Vyas, A.; Jennings, J.; Ellen, J.	2010	Are childbearing motivations getting in the way of STI prevention in young women?	10.1016/j.jadohealth.2009.11.015
Publication Type (Other)	Vvedenskaya, E. S.; Bykova, L. K.; Petrushov, P. K.	2010	High risk sexual behavior and reproductive health of Russian female students in the era of HIV/AIDS	N/A
Publication Type (Other)	Calderon, Y.; Chin, R.; Cowan, E.; Brusalis, C.; Zhan, C.; Leider, J.	2011	A validation study of high volume, rapid HIV testing in a south bronx municipal hospital	10.1111/j.1553-2712.2011.01073.x
Publication Type (Other)	Gannon, K.; Moreno, M.; Becker, T.	2011	Religion and sex in college freshmen: A longitudinal study of facebook	10.1203/PDR.0b013e3182304121
Publication Type (Other)	Garland, S. M.; Wark, J. D.; Tabrizi, S. N.; Jayasinghe, Y.; Moore, E.; Fletcher, A.; Gunasekaran, B.; Ahmed, N.; Fenner, Y.	2011	Recruiting via social networking sites for sexual health research (assessing chlamydia and HPV knowledge)	10.1136/sextrans-2011-050108.176
Publication Type (Other)	Hensel, D.; Dennis Fortenberry, J.	2011	Constructing an empirical model of sexual health and sexual decision making among adolescent women	10.1016/j.jadohealth.2010.11.028
Publication Type (Other)	Krawczyk, A.; Lau, E.; Perez, S.; Rosberger, Z.	2011	What do they know and what do they think they know? The influence of knowledge and perceived knowledge on human papillomavirus (HPV) vaccination decision-making	10.1002/pon.2078
Publication Type (Other)	Lou, J.-H.; Chen, S.-H.; Fu, C.-M.; Huang, S.-L.	2011	Development of sexual self-efficacy scale for adolescents in Taiwan	N/A
Publication Type (Other)	Martinez-Garcia, Genevieve	2011	'Sin querer queriendo': Exploring the factors associated with pregnancy prevention and pregnancy intention among Latino youth in montgomery county	N/A
Publication Type (Other)	NCT01310543	2011	Trial of the Teens and Toddlers Intervention	N/A
Publication Type (Other)	Otuonye, N. M.; Onwuatuelo, I. R.; Okwuzu, J. O.; Onwuamah, C. K.; Adeneye, A. K.; Oparaugo, C. T.; Akintude, G. B.; Uwandu, M. O.; Fowora, M. A.	2011	Adolescents' willingness to participate in HIV vaccine clinical trial preparedness in Nigeria	10.1097/01.qai.0000397386.44136.10
Publication Type (Other)	Overbeek, A.; van den Berg, M. H.; Louwé, L.; Hilders, C.; Veening, M. A.; Lambalk, C. B.; Stiggelbout, A. M.; van Dulmen-den Broeder, E.; Kuile, M. M. ter	2011	Practice, attitude and knowledge regarding fertility preservation techniques for women in the Netherlands (the PAK-study): Reports of the pilot study	10.1093/humrep/26.s1.87
Publication Type (Other)	Saewyc, E.; Marshall, S.	2011	Reducing homophobia in high school: The effects of “The Laramie Project” play and an integrated curriculum	10.1016/j.jadohealth.2010.11.227
Publication Type (Other)	Urech, C.; Alder, J.; Zanetti, R.; Rochlitz, C.; Tschudin, S.	2011	Fertility preservation in young female cancer patients: A study to assess knowledge, attitudes and intentions to use fertility preservation	10.1002/pon.2078

Reason for Exclusion/ Inclusion	Author(s)	Year	Title	DOI
Publication Type (Other)	Young, J.; Brown, A.; Carr, L.; Korte, J.; Modesitt, S.	2011	Television and Internet sources of information negatively associated with human papillomavirus vaccination among college-age women	10.1016/j.ygyno.2010.12.298
Publication Type (Other)	Bankovic, A.; Sedlecky, K.	2012	Sex education of adolescents in Belgrade, Serbia	10.3109/13625187.2012.673963
Publication Type (Other)	Gerhard, R. S.; Hsiao, W.; Ritenour, C.W.M.	2012	Sexual health education-where do men get their information?	10.1111/j.1743-6109.2012.02863.x
Publication Type (Other)	Gonc¸alves, H.; González, D.; Menezes, A.M.B.; Hallal, P. C.; Araújo, C.; Dumith, S.	2012	HIV/AIDS transmission knowledge among adolescents aged 11 years from Southern Brazil	10.1007/s10654-012-9722-6
Publication Type (Other)	Kettunen, C.; Robinson, R.; McIntrye, K.; Anderson, C.	2012	Survey to determine compliance with center for disease control recommendation for vaccination of adolescents	10.1016/j.ajic.2012.04.298
Publication Type (Other)	Lidaka, L.; Viberga, I.	2012	Risk factors for unwanted pregnancy that results in legal abortion in the age group 16-25 years in Latvia	10.3109/13625187.2012.673963
Publication Type (Other)	Lou, J.-H.; Chen, S.-H.; Eng, C.-J.; Lin, Y.-C.; Lin, F.-C.	2012	Development of sexual self-concept scale for adolescents	10.1111/j.1743-6109.2012.02645.x
Publication Type (Other)	NTR3692	2012	“Young people, adult worries”: RCT of an internet-based self-support method “Feel the ViBe” for children, adolescents and young adults exposed to family violence	N/A
Publication Type (Other)	Polan, J.; Sieving, R.; Pettingell, S.; Bearinger, L.; McMorris, B.	2012	Relationships between adolescent girls' socialemotional intelligence and their involvement in relational aggression and physical fighting	10.1016/j.jadohealth.2011.10.216
Publication Type (Other)	Sander, P.; Chung, S.; Ellen, J.; Matson, P.	2012	Missing data in a mobile phone daily diary study of adolescents	10.1093/aje/kws258
Publication Type (Other)	Williams, R. L.; Hensel, D. J.; Fortenberry, J. D.	2012	Predicting continuation of depot medroxyprogesterone acetate: Pregnancy prevention, pregnancy intention, and contraceptive beliefs among adolescent women	10.1016/j.jadohealth.2011.10.068
Publication Type (Other)	Bachorik, A. E.; Friedman, J. O.; Nucci-Sack, A. T.; Horowitz, C. R.; Diaz, A.	2013	Adolescent women's knowledge of and attitudes toward etonogestrel implants	10.1016/j.jadohealth.2012.10.211
Publication Type (Other)	Berglas, Nancy Faye	2013	Conceptualizing and measuring a rights-based approach to sexuality education	N/A
Publication Type (Other)	Brincat, A. M.; Azzopardi, L.	2013	Human papillomavirus awareness	10.1007/s11096-013-9801-0
Publication Type (Other)	Bumbul, E.; Starek, M.; Pietrzak, B.; Szymusik, I.; Wielgos, M.; Bumbuliene, Z.	2013	Usage patterns and accessibility of Emergency Contraception other-thecounter versus prescription-only	10.3109/13625187.2013.771860
Publication Type (Other)	Chen, Yu-ning; Hou, Chan-juan; Yao, Shu-qiao	2013	Reliability and validity of The Attitudes Toward Sexuality Scale for Chinese adolescents	10.1037/t06548-000;
Publication Type (Other)	Del Carmen Saeteros Hernández, R.; Ramos, G. S.; Piñero, J. P.	2013	Educational needs in sexual and reproductive health of college students. Polytechnic school of chimborazo-Ecuador	10.1111/jsm.12304
Publication Type (Other)	Hensel, D. J.; Fortenberry, J. D.	2013	Understanding the association of sexual health to health equity and well-being in adolescent women	10.1016/j.jadohealth.2012.10.034
Publication Type (Other)	Hernandez, Natalie	2013	Studying motivations to avoid pregnancy	10.1363/4522413
Publication Type (Other)	Jaruseviciene, L.; Meyer, S. de; Decat, P.; Zaborskis, A.; Auquilla, N.; Hagens, A.; Lazarus, J.	2013	Gender attitudes and sexual behaviour in Bolivian and Ecuadorian adolescents	10.3109/13625187.2013.793038
Publication Type (Other)	Javadnoori, M.; Latifinejad Rudsari, R.; Hasanpour, M.; Hazavehei, S.M.M.; Taghipour, A.	2013	Female adolescents attitudes toward sexual and reproductive health education in Iran	N/A
Publication Type (Other)	Lazarus, L.; Marshall, Z.; LeBlanc, S.; Shaw, A.; Martin, A.; Dalton, C.; White, A.; Schreiber, Y. S.; Tyndall, M. W.	2013	Community engagement and ownership in the development and design of PROUD, an Ottawa CBPR study	N/A
Publication Type (Other)	Le Den, M.	2013	Public authorities and teenage pregnancy in France: The perception of the phenomena at the centre of prevention campaigns	10.3109/13625187.2013.793038
Publication Type (Other)	Marinho, S.; Anastacio, Z.	2013	The impact of a sex education program for students of 2nd and 3rd cycle of basic education	10.1016/S0212-6567(13)70033-7

Reason for Exclusion/ Inclusion	Author(s)	Year	Title	DOI
Publication Type (Other)	NCT01772628	2013	Parenting Styles and Parent-Offspring Communication on Sexuality Issues	N/A
Publication Type (Other)	NCT01795885	2013	Trial of the Effect of "16 and Pregnant" on Teen Girls' Attitudes About Pregnancy	N/A
Publication Type (Other)	Nordin, R.; Chong, C. Y.; Low, Z. M.; Chung, C. M.; Wong, C.Y.T.	2013	Validity and reliability of the Sexual Health Education for Adolescents (SHEA) questionnaire	10.3109/13625187.2013.771860
Publication Type (Other)	Reis, M.; Ramiro, L.; Matos, M. G. de	2013	Sexual victimization in portuguese women	10.1111/jsm.12304
Publication Type (Other)	Sagor, R. S.; Golding, J.; Blake, D. R.	2013	Knowledge is power: Effect of educational interventions on readiness for chlamydia screening	10.1016/j.jadohealth.2012.10.058
Publication Type (Other)	Sebastian, R.; Ramos, M. M.; Fairbrother, G.; McGrath, J.	2013	Assessing youth engagement and quality of care in school-based health centers: The youth engagement with health services (yehs!) survey	10.1016/j.jadohealth.2012.10.025
Publication Type (Other)	Ucar, T.; Golbasi, Z.; Senturk Erenel, A.	2013	Sexuality and the internet: A study on the perspectives of Turkish university students	10.3109/13625187.2013.793038
Publication Type (Other)	Ur Rehman, K.; Shabbir Sheikh, S.; Asif Mahmood, M.; Sultan, T.	2013	The female sexual function index FSFI: Translation, validation and crosscultural adaptation of an urdu version for use in Pakistan	10.1111/jsm.12414
Publication Type (Other)	Williams, R. L.; Hensel, D. J.; Fortenberry, J. D.	2013	The influence of sexual health on contraceptive method use: A longitudinal analysis of monogamous adolescent sexual relationships	10.1016/j.jadohealth.2012.10.210
Publication Type (Other)	Barbour, W.; Tapley, A.; Rodgers, J.; Prickett, C.; Galbraith, J. W.	2014	Comparison of HIV risky behaviors and knowledge reported by adolescents and parents presenting to a pediatric emergency department	10.1111/acem.12365
Publication Type (Other)	Richter, Diana; Brähler, Elmar; Strauß, Bernhard	2014	Diagnostische Verfahren in der Sexualwissenschaft	N/A
Publication Type (Other)	Cavalcanti, M.; Lopes, M. H.	2015	Use of the portuguese version of the educational material “your pelvic floor” among Brazilian teenagers	10.1002/nau.22830
Publication Type (Other)	Delbaere, I.; Vanderplancke, T.; Bogaerts, A.; Provoost, V.; Sutter, P. de; Tyden, T.	2015	Fertility awareness in the Flemish population: Optimism can be disadvantageous	10.1093/humrep/30.Supplement-1.1
Publication Type (Other)	Denison, H. J.; Dennison, E. M.; Jutel, A.	2015	Recruiting to sexual health studies: Possible strategies identified through focus group discussions	10.1136/sextrans-2015-052270.240
Publication Type (Other)	Garcia De La Borbolla Sanchez, V.; Hermoso Limón, A.; Gómez Gutiérrez, M.; Noa Hortal, M.; Núñez Garcés, M.	2015	Adolescence and groups: An experience following pichon-rivière's operative group model with adolescent population in a community mental health centre in Southern Spain	10.1007/s00787-015-0714-4
Publication Type (Other)	Hendriks, J.; Fyfe, S.; Doherty, D. A.; Styles, I.; Jacques, A.; Burns, S.; Hickey, M.; Skinner, S. R.	2015	Importance of adolescents' attitudes towards contraception, early parenthood and abortion	N/A
Publication Type (Other)	Ibeneme, S.; Nnaji, R.; Ibeneme, G.	2015	Awareness and compliance to screening (pap smear) test for cervical cancer among sexually active undergraduate female students	10.1016/j.physio.2015.03.1996
Publication Type (Other)	Kondapalli, L. A.; Barnhart, K. B.	2015	Prospective cohort study of autism, neurodevelopment, and behavior in young children conceived by Assisted Reproductive Technology (ART): Cause for concern or reassurance?	10.1002/bdra.23387
Publication Type (Other)	Kudesia, R.; Chernyak, E.; McAvey, B.	2015	Creation & validation of the fertility and infertility treatment knowledge survey (FIT-KS)	N/A
Publication Type (Other)	Kudesia, R.; Petti, M.; Talib, H. J.; Pollack, S. E.	2015	Fertility counseling for adolescent and adult gynecologic patients	10.1097/01.AOG.0000463717.44698.6a
Publication Type (Other)	Maguire, L.; Aventin, A.; Lohan, M.; Clarke, M.	2015	What do young people really understand when completing questionnaires? Lessons learnt from developing a questionnaire to measure behavioural outcomes in a sexual health trial	N/A
Publication Type (Other)	Robinson, K.	2015	Respecting gender and sexual difference to promote sexual health: Making schools safe spaces for all	10.1136/sextrans-2015-052270.24
Publication Type (Other)	Shipp, M.; Gregson, S.	2015	The victorian aboriginal health service (VAHS) conducted a young people's sexual health and sexually transmissible infections and blood borne virus (STI/BBV) knowledge, attitudes and behaviour survey in november 2014	10.1136/sextrans-2015-052270.245
Publication Type (Other)	Yuruk, E.; Gul, A.; Serefoglu, E.	2015	Frequency of nocturnal emissions and its psychological consequences among sexually Naïve religious teenagers	10.1111/jsm.12826/abstract

Reason for Exclusion/ Inclusion	Author(s)	Year	Title	DOI
Publication Type (Other)	Alvis Zakzuk, J.; Salcedo Mejía, F.; Alvis Guzman, N.; Alvis Zakzuk, J. Y.; Alvis Zakzuk, N. R.	2016	Prevalence of use of contraceptive methods in young poor people from a region of Colombia	N/A
Publication Type (Other)	Baird, Sierra Marie	2016	Expected profiles and temporal stability of the LOOK	N/A
Publication Type (Other)	Brase, Gary L.	2016	The relationship between positive and negative attitudes towards children and reproductive intentions	10.1016/j.paid.2015.10.053
Publication Type (Other)	Hall, K.; Manu, A.; Morhe, E.; Loll, D.; Ela, E.; Kolenic, G.; Dozier, J.; Challa, S.; Harris, L.; Adanu, R.; Zochowski, M.; Boayke, A.; Dalton, V.	2016	Development of a scale tomeasure adolescent sexual and reproductive health stigma	10.1016/j.contraception.2016.07.151
Publication Type (Other)	Kenny, L. J.; Bostock, N. J.; Parkhurst, A.	2016	Exploring young people's attitudes towards routine health screening in a UK secondary school	10.1136/archdischild-2016-310863.129
Publication Type (Other)	Kumar, A.; Bhardwaj, P.; Gupta, P.; Srivastava, J. P.	2016	A study of HIV/AIDS related knowledge and attitudes amongst the engineering College students	10.5958/0976-5506.2016.00114.5
Publication Type (Other)	Parekh, Jenita	2016	Implementation and replication of evidence based sexual reproductive health programs	N/A
Publication Type (Other)	Saleh, A.; Morgan, A.; Arrington-Sanders, R.	2016	Sources, type and use of social support during early sexual development of black gay and bisexual adolescent males	N/A
Publication Type (Other)	Slater, Holli M.	2016	Predicting condom use behavior in sexually active adolescents: Application of the health belief model and developmental assets framework	N/A
Publication Type (Other)	Weaver, Racquel D.	2016	An assessment of sexually transmitted disease knowledge among seventh grade students	N/A
Publication Type (Other)	Ahmed, Z. D.; Ibrahim, B. S.; Bolaji, M. L.; Mohammed, Y.; Nguku, P.	2017	Knowledge and utilization of contraceptive devices among unmarried undergraduate students ofa tertiary institution in Kano State, Nigeria 2016	10.1111/jog.13393
Publication Type (Other)	Clark, B.; Jarin, J.; Strang, J.; Call, D.; Solages, M.; Gomez-Lobo, V.	2017	Transgender adolescent attitudes towards their future fertility	10.1016/j.jpag.2017.03.127
Publication Type (Other)	Fava, V.; Melis, I.; Colombo, M.; Silvaggi, M.; Rossetto, C.; Nanini, C.; Artioli, C.; Simone, S.; Malandrino, C.; Di Santo, S. G.	2017	Condom negotiation and sexual health in young Italian women	N/A
Publication Type (Other)	Hurley, L.; Wilkins, R.	2017	Measuring abortion stigma as it affects young women in Benin, Burkina Faso, India and Pakistan	N/A
Publication Type (Other)	Lohan, Maria; Aventin, Áine; Maguire, Lisa; Curran, Rhonda; McDowell, Clíona; Agus, Ashley; Donaldson, Cam; Clarke, Mike; Linden, Mark; Kelly, Carmel; McDaid, Lisa; Dunne, Laura; O'Halloran, Peter	2017	Increasing boys' and girls' intentions to avoid teenage pregnancy: a cluster randomised controlled feasibility trial of an interactive video drama-based intervention in post-primary schools in Northern Ireland	10.3310/phr05010
Publication Type (Other)	Michael, K.	2017	Adolescents' sexual communication with their parents, peers, and dating partners	N/A
Publication Type (Other)	Michael, K.; Yakhnich, L.	2017	The contribution of relations with parents and peers to adolescents' sexual risk-taking behavior	N/A
Publication Type (Other)	Murgatroyd-Giffen, A.; Bishopp, D.	2017	The influence of pornography on young people's sexual health and behaviour	N/A
Publication Type (Other)	NCT03065842	2017	A Pre and Post Test Intervention Design to Prevent Abortion and Contraceptive-use Stigma Among School Youths in Kenya	N/A
Publication Type (Other)	NCT03065842	2017	A Pre and Post Test Intervention Design to Prevent Abortion and Contraceptive-use Stigma Among School Youths in Kenya	N/A
Publication Type (Other)	NCT03304015	2017	HERrespect Evaluation	N/A
Publication Type (Other)	NCT03348813	2017	HIV/STI Prevention Among Black Adolescents With Mental Illnesses (Project GOLD)	N/A
Publication Type (Other)	NCT03384251	2017	Assessing an Educational Intervention Program on Knowledge, Attitude and Behaviour Towards Pregnancy Prevention Based on Health Belief Model Amongst Adolescent Girls in Northern Ghana	N/A

Reason for Exclusion/ Inclusion	Author(s)	Year	Title	DOI
Publication Type (Other)	Rashid, A.	2017	Assessment of comprehensive HIV/AIDS knowledge level among in-school & community level adolescents of noakhali and Lakshmipur District, Bangladesh	10.1136/sextrans-2017-053264.498
Publication Type (Other)	Rosenthal, S. L.; Roche, A. M. de; Catallozzi, M.; Breitkopf, C. R.; Lisa, S.I.P.P.; Chang, J.; Francis, J. K.; Mauro, C. M.	2017	Adolescents' and their parents' attitudes over time about parental involvement in clinical research	10.1136/sextrans-2017-053264.596
Publication Type (Other)	Sorpreso, I.C.E.; Takiuti, A. D.; Baracat, E. C.; Júnior, J.M.S.; Da Silva, A.T.M.; Abreu, L. C. de	2017	Acceptability and knowledge HPV vaccine: Preliminar results	10.1089/jwh.2017.29011.abstracts
Publication Type (Other)	Abu-Zaineh, Mohammad; Woode, Maame Esi; Giacaman, Rita	2018	Youth wellbeing through the lens of the Senian capability approach: insights from the occupied Palestinian territory: a cross-sectional study	10.1016/S0140-6736(18)30676-7
Publication Type (Other)	Alphonso, A.; Olezeski, C.; Kallen, A.; Cron, J.	2018	Fertility knowledge and preferences among transgender and gender non-conforming adolescents	N/A
Publication Type (Other)	Brown, J. L.; Marais, L.; Sharp, C.; Cloete, J.; Lenka, M.; Rani, K.; Sales, J. M.	2018	Understanding the family planning and HIV prevention needs of south african adolescent girls: A cultural consensus modeling approach	N/A
Publication Type (Other)	Ehrbar, V.; Urech, C.; Rochlitz, C.; Zanetti Daellenbach, R.; Moffat, R.; Stiller, R.; Germeyer, A.; Nawroth, F.; Dangel, A.; Findeklee, S.; Tschudin, S.	2018	Randomized controlled trial on the effect of an online, decision aid for young female cancer patients considering fertility preservation	10.1093/humrep/33.Supplement_1.1
Publication Type (Other)	Gaur, N.	2018	Sexual and reproductive health awareness among urban adolescent girls in western India	10.1002/ijgo.12583
Publication Type (Other)	MacCormick, H. K.; George, R.	2018	Sexual and gender minorities curriculum within obstetrical anesthesia fellowship programs	10.1007/s12630-018-1223-y
Publication Type (Other)	Meiksin, R.; Ponsford, R.; Bonell, C.	2018	Assessment of survey items on social norms relating to sexual behaviour and dating and relationship violence among young adolescents in England: cognitive interviews within two pilot studies	10.1016/S0140-6736(18)32888-5
Publication Type (Other)	NCT03484169	2018	Effects of Pelvic Patterns of Proprioceptive Neuromuscular Facilitation in the Pelvic Floor Muscles	N/A
Publication Type (Other)	NCT03715335	2018	Adolescent Sexually Transmitted Infection Screening in the Emergency Department	N/A
Publication Type (Other)	Olson, R. M.; García-Moreno, C.	2018	Virginity testing: A systematic review of medical reliability and health impacts	10.1002/ijgo.12582
Publication Type (Other)	Pulerwitz, J.; Mathur, S.; Woznica, D.	2018	How does relationship power influence HIV risk among adolescent girls and young women in Kenya?	10.1089/aid.2018.5000.abstracts
Publication Type (Other)	Ramaiya, Astha	2018	'Time of the month': A mixed-method study to understand and improve menstrual health and hygiene management in rural North India	10.17918/D8N370
Publication Type (Other)	Thoma, Brian C.	2018	Parent-adolescent communication about sex and condom use among young men who have sex with men: An examination of the theory of planned behavior	10.1093/abm/kay002
Publication Type (Other)	Wachsmann, H.	2018	Addressing abortion stigma as it affects young people in South Asia and West Africa	10.1080/13625187.2018.1442911
Publication Type (Other)	Walker, S. A.	2018	Review of Current Risk Assessment Practices	10.1016/j.jaac.2018.07.227
Publication Type (Other)	Win, S. S.; Lasimbang, H. B.; Hossain, T.; Myint, T.; Win, M.M.C.; Aung, S.N.L.	2018	Comprehensive sexuality education (CSE) among first year students of University Malaysia Sabah (UMS)	10.1002/ijgo.12582
Publication Type (Other)	Zimcikova, E.; Regnerova, V.; Kubena, A.; Mala-Ladova, K.	2018	Knowledge and attitudes regarding cervical cancer and human papillomavirus vaccination in Czech secondary school students	10.1002/pds.4629
Publication Type (Other)	Bain, Luchuo Engelbert; Zweekhorst, Marjolein B. M.; Amoakoh-Coleman, Mary; Muftugil-Yalcin, Seda; Omolade, Abejirinde Ibukun-Oluwa; Becquet, Renaud; Buning, Tjard de Cock	2019	To keep or not to keep? Decision making in adolescent pregnancies in Jamestown, Ghana	10.1371/journal.pone.0221789
Publication Type (Other)	Handschuh, Caroline	2019	Peer and parental relationships and their association with adolescent sexual behaviors	10.7916/d8-y2ge-4s28
Publication Type (Other)	McDonagh, L.; Hunt, E.; Naidoo, C.; Saunders, J.; Dunbar, K.; Amlot, R.; Weston, D.	2019	Psychosocial factors associated with chlamydia retesting among young people in the UK	10.1136/sextrans-2019-sti.453

Reason for Exclusion/ Inclusion	Author(s)	Year	Title	DOI
Publication Type (Other)	NCT03912753	2019	Building Mobile HIV Prevention and Mental Health Support in Low-resource Settings	N/A
Publication Type (Other)	NCT03995953	2019	Integrated Care Delivery of HIV Prevention and Treatment in AGYW in Zambia	N/A
Publication Type (Other)	NCT04131465	2019	Home HIV Testing for Older Adults in South Africa	N/A
Publication Type (Other)	Svab, A.; Kogovsek, T.; Kuhar, R.	2019	HP-07-003 First sexual intercourse among high school students in Slovenia	10.1016/j.jsxm.2019.03.149
Publication Type (Other)	TCTR20191205001	2019	Effect of An Intervention to Improve the Sexual and Reproductive Health Literacy of University Students in Magway City of Myanmar	N/A
Publication Type (Other)	Wilkinson, T. A.; Meredith, A. H.; Vielott, T. L.; Stewart, J. J.; Kaur, J.; Meagher, C. G.; Ott, M. A.	2019	Assessment of Adolescent Decision-Making Capacity For Pharmacist Access To Hormonal Contraception	10.1016/j.jadohealth.2018.10.035
Publication Type (Other)	Woods, J. L.; Hensel, D. J.	2019	Male Genitourinary Exam Assessment: A Tool Validation	10.1016/j.jadohealth.2018.10.178
Publication Type (Other)	Conlon, J. L.; Monaghan, M.; Malcolm, S.	2020	199. Design of a Survey Instrument to Evaluate Primary Care Provider Behavior in the Diagnosis and Management of PCOS in Adolescents	10.1016/j.jadohealth.2019.11.202
Publication Type (Other)	Divya, K. V.; Deepika, M.; Kalaivani, S.; Priyadharshini, A.; Vanitha, K.	2020	Knowledge on maternal and child health service among women in reproductive age group in mahabalipuram, kanchipuram district, tamil nadu, india	N/A
Publication Type (Other)	Guss, C. E.; Assefa, I.; Stamoulis, C.; Pilcher, S.	2020	153. HIV Knowledge and Acceptability of Rapid HIV Testing Among Transgender Adolescents in a Multi-Disciplinary Gender Clinic	10.1016/j.jadohealth.2019.11.156
Publication Type (Other)	Guss, Carly E, MD, MPH; Assefa, Ida, BA; Stamoulis, Catherine, MS, PhD; Pilcher, Sarah, MSN, RN, CPNP	2020	HIV KNOWLEDGE AND ACCEPTABILITY OF RAPID HIV TESTING AMONG TRANSGENDER ADOLESCENTS IN A MULTI-DISCIPLINARY GENDER CLINIC	
Publication Type (Other)	Minnis, A. M.; Browne, E. N.; Chavez, M.; McGlone, L.; Raymond-Flesch, M.; Auerswald, C.	2020	224. Neighborhood Social Environment Associations with Initiation of Sexual Activity in Early and Middle Adolescence: Opportunities for Promoting Positive Sexual Health Outcomes	10.1016/j.jadohealth.2019.11.227
Publication Type (Other)	NCT04307849	2020	Youth-friendly Sexual and Reproductive Healthcare Pilot in Mumbai, India	N/A
Publication Type (Other)	Secor-Turner, M.; Owino, J.; Randall, B.	2020	33. Meeting the Sexual Health Needs of Immigrant and Refugee Youth	10.1016/j.jadohealth.2019.11.036
Publication Type (Other)	Camenga, Deepa R, MD, MHS; Berry, Amanda, PhD, CRNP; Constantine, Melissa, PHD, MPA; Gahagan, Sheila, MD, MPH; Newman, Diane, DNP; Rudser, Kyle, PhD; Coyne-Beasley, Tamera, MD, MPH; Kane-Low, Lisa, PhD, RN, CNM; Shoham, David, PhD; Scal, Peter, MD, MPH	2021	CREATING A RESEARCH PROGRAM TO PROMOTE BLADDER HEALTH IN ADOLESCENT WOMEN	
Publication Type (Other)	Domogauer, J. D.; Ganey-Aquino, S.; Cabrera, A.; Pietrzyk-Busta, B.; Huppert, N. E.; Schiff, P. B.; Lymberis, S. C.	2021	A Sexual and Gender Minority Inclusive Tool to Identify and Reduce Psychological Distress Related to Vaginal Brachytherapy Treatment	10.1016/j.ijrobp.2021.07.1014
Publication Type (Other)	Khetarpal, S. K.; Szoko, N.; Culyba, A. J.; Ragavan, M.	2021	97. The Role of Parental Monitoring as a Protective Factor Against Youth Violence Victimization	10.1016/j.jadohealth.2020.12.106
Publication Type (Other)	NCT04716400	2021	Effectiveness Study of the Intervention "Stop Sexual Harassment" in Secondary School	N/A
Publication Type (Other)	Nelson, K.; Magut, F.; Mulwa, S.; Khagayi, S.; Ziraba, A.; Kwaro, D.; Floyd, S.; Birdthistle, I.	2021	Association between DREAMS invitation and attitudes towards gender norms amongst young women in urban and rural Kenya, measured using an adapted and validated version of the GEM Scale	10.1002/jia2.25659
Publication Type (Other)	Panton, C.; Zhang, A. Y.; Dalomba, N. F.; Taliaferro, E.; Bourne, S.; Lee, C.; Roberts, M.; Magee, S.	2021	Sexed by brown med: Evaluating the impact of medical student-led sexual health curriculum on middle schoolers' attitudes, beliefs, and knowledge	10.1542/peds.147.3-MeetingAbstract.220-a
Publication Type (Other)	Quinn, G. P.; Pratt-Chapman, M. L.; Meersman, S. C.; Chang, S.; Kamen, C. S.; Maingi, S.; Merrill, J. K.; Schabath, M. B.	2021	Barriers and facilitators to sexual orientation and gender identity (SOGI) data collection	10.1200/JCO.2021.39.suppl.e18520
Publication Type (Other)	SLCTR/2021/014	2021	A Cluster-Randomized Trail (CRT) to compare the effectiveness of a Facebook-based intervention to improve knowledge, attitude, and skills on sexual and	N/A

Reason for Exclusion/ Inclusion	Author(s)	Year	Title	DOI
			reproductive health among youth in vocational training institutions in the Western province	
Study Aim	Holland-Hall, C. M.; Wiesenfeld, H. C.; Murray, P. J.	2002	Self-collected vaginal swabs for the detection of multiple sexually transmitted infections in adolescent girls	10.1016/s1083-3188(02)00197-3
Study Aim	Jennings, Terri E.; Lucenko, Barbara A.; Malow, Robert M.; Dévieux, Jessy G.	2002	Audio-CASI vs interview method of administration of an HIV/STD risk of exposure screening instrument for teenagers	10.1258/095646202320753754
Study Aim	Nwokolo, N.; McOwan, A.; Hennebry, G.; Chislett, L.; Mandalia, S.	2002	Young people's views on provision of sexual health services	10.1136/sti.78.5.342
Study Aim	Olsen, Christie L.; Santarsiero, Elizabeth C.; Spatz, Diane	2002	Qualitative analysis of African-American adolescent females' beliefs about emergency contraceptive pills	10.1016/s1083-3188(02)00194-8
Study Aim	Wingood, G. M.; Diclemente, R. J.; Harrington, K.; Davies, S. L.	2002	Body image and African American females' sexual health	10.1089/15246090260137608
Study Aim	Betts, S. C.; Peterson, D. J.; Huebner, A. J.	2003	Zimbabwean adolescents' condom use: What makes a difference? Implications for intervention	10.1016/S1054-139X(02)00565-7
Study Aim	Bhalwar, R.; Jayaram, J.	2003	Community based study of AIDS awareness and attitudes among school and college-going teenagers from rural background	10.1016/S0377-1237(03)80094-1
Study Aim	Holschneider, S.O.M.; Alexander, C. S.	2003	Social and psychological influences on HIV preventive behaviors of youth in Haiti	10.1016/S1054-139X(02)00418-4
Study Aim	Smith, Thomas E.; Steen, Julie A.; Spaulding-Givens, Jennifer; Schwendinger, Andrea	2003	Measurement in abstinence education: Critique and Recommendations	10.1177/0163278703026002004
Study Aim	Taffa, N.; Sundby, J.; Bjune, G.	2003	Reproductive health perceptions, beliefs and sexual risk-taking among youth in Addis Ababa, Ethiopia	10.1016/S0738-3991(02)00090-3
Study Aim	Tavakol, Mohsen; Torabi, Sima; Gibbons, Cathy	2003	A quantitative survey of knowledge of reproductive health issues of 12-14-year-old girls of different ethnic and religious backgrounds in Iran: Implications for education	10.1080/1468181032000119113
Study Aim	Farr, C.; Brown, J.; Beckett, R.	2004	Ability to empathise and masculinity levels: Comparing male adolescent sex offenders with a normative sample of non-offending adolescents	10.1080/10683160310001597153
Study Aim	Helweg-Larsen, Karin; Sundaram, Vanita; Curtis, Tine; Larsen, Helmer Bøving	2004	The Danish Youth Survey 2002: asking young people about sensitive issues	10.3402/ijch.v63i0.17880
Study Aim	Hewett, Paul C.; Erulkar, Annabel S.; Mensch, Barbara S.	2004	The Feasibility of Computer-Assisted Survey Interviewing in Africa: Experience from Two Rural Districts in Kenya	10.1177/0894439304263114
Study Aim	Plummer, Mary L.; Wight, Daniel; Ross, David A.; Balira, Rebecca; Anemona, Alessandra; Todd, Jim; Salamba, Zachayo; Obasi, Angela I. N.; Grosskurth, Heiner; Changalunga, John; Hayes, Richard J.	2004	Asking semi-literate adolescents about sexual behaviour: the validity of assisted self-completion questionnaire (ASCQ) data in rural Tanzania	10.1111/j.1365-3156.2004.01254.x
Study Aim	Podlinski, K.; Porsch, B.; Krüssel, J. S.; Bender, H. G.; Beckmann, M. W.; Binder, H.	2004	Knowledge about contraception of women in the reproductive age: The German results of a survey initiated by the European Group of Contraception (EGOC)	10.1055/s-2004-821163
Study Aim	Stallworth, JoAna; Roofe, Michele; Clark, Leslie F.; Ehiri, John E.; Mukherjee, Snigdha; Person, Sharina; Jolly, Pauline E.	2004	Predictors of sexual involvement among adolescents in rural Jamaica	10.1515/ijamh.2004.16.2.165
Study Aim	Walker, D. M.; Torres, P.; Gutierrez, J. P.; Flemming, K.; Bertozzi, S. M.	2004	Emergency contraception use is correlated with increased condom use among adolescents: Results from Mexico	10.1016/j.jadohealth.2004.07.001
Study Aim	Wang, R.-H.; Hsu, M.-T.; Wang, H.-H.	2004	Potential Factors Associated with Contraceptive Intention among Adolescent Males in Taiwan	10.1016/s1607-551x(09)70094-4
Study Aim	Adams-Curtis, Leah E.; Bloesch, Emily; Forbes, Gordon B.; Jobe, Rebecca L.; White, Kay B.	2005	Perceptions of dating violence following a sexual or nonsexual betrayal of trust: effects of gender, sexism, acceptance of rape myths, and vengeance motivation	10.1007/s11199-005-1292-6
Study Aim	Buhi, Eric R.	2005	Reliability reporting practices in rape myth research	10.1111/j.1746-1561.2005.tb00012.x
Study Aim	Hazarika, N. C.; Mahanta, J.	2005	Perceptions of unmarried young women regarding family size, sexually transmitted diseases at residential regional institutes in northeastern India	N/A
Study Aim	Potdar, Rukmini; Koenig, Michael A.	2005	Does Audio-CASI improve reports of risky behavior? Evidence from a randomized field trial among young urban men in India	10.1111/j.1728-4465.2005.00048.x
Study Aim	Skowron, Christine	2005	Differentiation and predictive factors in adolescent sexual offending	N/A

Reason for Exclusion/ Inclusion	Author(s)	Year	Title	DOI
Study Aim	Swim, Janet K.; Mallett, Robyn; Russo-Devosa, Yvonne; Stangor, Charles	2005	JUDGMENTS OF SEXISM: A COMPARISON OF THE SUBTLETY OF SEXISM MEASURES AND SOURCES OF VARIABILITY IN JUDGMENTS OF SEXISM	10.1111/j.1471-6402.2005.00240.x
Study Aim	Wilson, Kelly L.; Goodson, Patricia; Pruitt, B. E.; Buhi, Eric; Davis-Gunnels, Emily	2005	A Review of 21 Curricula for Abstinence-Only-Until-Marriage Programs	10.1111/j.1746-1561.2005.00003.x
Study Aim	Afable-Munsuz, Aimee; Brindis, Claire D.	2006	Acculturation and the sexual and reproductive health of Latino youth in the United States: a literature review	10.1363/psrh.38.208.06
Study Aim	Brown, Necole; Naman, Priya; Homel, Peter; Fraser-White, Marilyn; Clare, Richard; Browne, Ruth	2006	Assessment of Preventive Health Knowledge and Behaviors of African-American and Afro-Caribbean Women in Urban Settings	N/A
Study Aim	Deptula, Daneen P.; Henry, David B.; Shoeny, Michael E.; Slavick, John T.	2006	Adolescent sexual behavior and attitudes: a Costs and Benefits approach	10.1016/j.jadohealth.2004.08.026
Study Aim	Ferguson, Alan G.; Morris, Chester N.; Kariuki, Cecilia W.	2006	Using diaries to measure parameters of transactional sex: An example from the Trans-Africa highway in Kenya	10.1080/13691050600665006
Study Aim	Le, Linh Cu; Blum, Robert W.; Magnani, Robert; Hewett, Paul C.; Do, Hoa Mai	2006	A pilot of audio computer-assisted self-interview for youth reproductive health research in Vietnam	10.1016/j.jadohealth.2005.07.008
Study Aim	Montijo, S. R.; Rodriguez, D. M.; Arroyo, B. F.; Cervantes; Villegas, R. B.; Anaya, EDGY; Campuzano, M. D.R.; Pozo, R. H.	2006	Behavioural training in partner communication and correct condom use skills	N/A
Study Aim	Mpofu, Elias; Caldwell, Linda; Smith, Edward; Flisher, Alan J.; Mathews, Catherine; Wegner, Lisa; Vergnani, Tania	2006	Rasch Modeling of the Structure of Health Risk Behavior in South African Adolescents	N/A
Study Aim	Reddy, M. K.; Fleming, M. T.; Howells, N. L.; Rabenhorst, M. M.; Casselman, R.; Rosenbaum, A.	2006	Effects of method on participants and disclosure rates in research on sensitive topics	N/A
Study Aim	Rohner, Jean Christophe; Björklund, Fredrik	2006	Do self-presentation concerns moderate the relationship between implicit and explicit homonegativity measures?	10.1111/j.1467-9450.2006.00522.x
Study Aim	Salako, A. A.; Iyaniwura, C. A.; Jeminusi, O. A.; Sofowora, R.	2006	Sexual behaviour, contraception and fertility among in-school adolescents in Ikenne Local Government, south-western Nigeria	N/A
Study Aim	Aras, Sahbal, MD; Semin, Semih, MD, PhD; Gunay, Turkan, MD; Orcin, Esmahan, MS; Ozan, Sema, MD	2007	Sexual Attitudes and Risk-Taking Behaviors of High School Students in Turkey*	10.1111/j.1746-1561.2007.00220.x
Study Aim	Bruine De Bruin, W.; Downs, J. S.; Fischhoff, B.; Palmgren, C.	2007	Development and evaluation of an HIV/AIDS knowledge measure for adolescents focusing on misconceptions	10.1300/J499v08n01_03
Study Aim	Hansen, T.; Skjeldestad, F. E.	2007	Adolescents: Is there an association between knowledge of oral contraceptives and profession of provider?	10.1080/13625180701475558
Study Aim	Holmberg, Lars I.; Hellberg, Dan	2007	Health, health-compromising behavior, risk-taking behavior and sexuality in female and male high school students in vocational and theoretical programs in Sweden	10.1515/IJAMH.2007.19.4.459
Study Aim	Iriyama, Shigemi; Nakahara, Shinji; Jimba, Masamine; Ichikawa, Masao; Wakai, Susumu	2007	AIDS health beliefs and intention for sexual abstinence among male adolescent students in Kathmandu, Nepal: a test of perceived severity and susceptibility	10.1016/j.puhe.2006.08.016
Study Aim	Kugler, Kari C.; Komro, Kelli A.; Stigler, Melissa H.; Mnyika, Kagoma S.; Masatu, Melkiory; Aastrom, Anne N.; Klepp, Knut-Inge	2007	The reliability and validity of self-report measures used to evaluate adolescent HIV/AIDS prevention programs in sub-Saharan Africa	10.1521/aeap.2007.19.5.365
Study Aim	Lakshmi, P.V.M.; Gupta, N.; Kumar, R.	2007	Psychosocial predictors of adolescent sexual behavior	10.1007/s12098-007-0170-3
Study Aim	Lederman, Regina P.; Chan, Wenyaw; Roberts-Gray, Cynthia	2007	Predictors of middle school youth educational aspirations: Health risk attitudes, parental interactions, and parental disapproval of risk	N/A
Study Aim	Thomas, Charles L.; Dimitrov, Dimiter M.	2007	Effects of a teen pregnancy prevention program on teens' attitudes toward sexuality: A latent trait modeling approach	10.1037/0012-1649.43.1.173
Study Aim	Zimmerman, Rick S.; Noar, Seth M.; Feist-Price, Sonja; Dekthar, Olga; Cupp, Pamela K.; Anderman, Eric; Lock, Sharon	2007	Longitudinal test of a multiple domain model of adolescent condom use	10.1080/00224490701629506
Study Aim	Baker, Amy J. L.; Gries, Len; Schneiderman, Mel; Parker, Rob; Archer, Marc; Friedrich, Bill	2008	Children with problematic sexualized behaviors in the child welfare system	N/A
Study Aim	Conron, Kerith Jane; Scout; Austin, S. Bryn	2008	"Everyone has a right to, like, check their box:" findings on a measure of gender identity from a cognitive testing study with adolescents	10.1080/15574090802412572

Reason for Exclusion/ Inclusion	Author(s)	Year	Title	DOI
Study Aim	Everson, Mark D.; Smith, Jamie B.; Hussey, Jon M.; English, Diana; Litrownik, Alan J.; Dubowitz, Howard; Thompson, Richard; Knight, Elizabeth Dawes; Runyan, Desmond K.	2008	Concordance between adolescent reports of childhood abuse and child protective service determinations in an at-risk sample of young adolescents	10.1177/1077559507307837
Study Aim	Gray, Susan H.; Austin, S. Bryn; Huang, Bin; Frazier, A. Lindsay; Field, Alison E.; Kahn, Jessica A.	2008	Predicting sexual initiation in a prospective cohort study of adolescents	10.1001/archpediatrics.2007.12
Study Aim	Hennessy, Michael; Bleakley, Amy; Fishbein, Martin; Jordan, Amy	2008	Validating an index of adolescent sexual behavior using psychosocial theory and social trait correlates	10.1007/s10461-007-9272-1
Study Aim	Ma, Mindy; Kibler, Jeffrey L.; Dollar, Katherine M.; Sly, Kaye; Samuels, Deanne; Benford, Melissa White; Coleman, Melissa; Lott, LaTonya; Patterson, Kevin; Wiley, Floyd	2008	The relationship of character strengths to sexual behaviors and related risks among African American adolescents	10.1080/10705500802365573
Study Aim	Mavhu, Webster; Langhaug, Lisa; Manyonga, Bothwell; Power, Robert; Cowan, Frances	2008	What is 'sex' exactly? Using cognitive interviewing to improve the validity of sexual behaviour reporting among young people in rural Zimbabwe	10.1080/13691050801948102
Study Aim	Vannier, Sarah A.; O'Sullivan, Lucia F.	2008	The feasibility and acceptability of handheld computers in a prospective diary study of adolescent sexual behaviour	N/A
Study Aim	Younge, Sinead N.; Salazar, Laura F.; Crosby, Richard F.; DiClemerite, Ralph J. ..; Wingood, Gina M.; Rose, Eve	2008	Condom use at last sex as a proxy for other measures of condom use: Is it good enough?	N/A
Study Aim	Bamidele, James Olusegun; Abodunrin, Olugbemiga Lanre; Adebimpe, Wasiu Olalekan	2009	Sexual behavior and risk of HIV/AIDS among adolescents in public secondary schools in Osogbo, Osun State, Nigeria	10.1515/ijamh.2009.21.3.387
Study Aim	Dariotis, Jacinda K.; Pleck, Joseph H.; Sonenstein, Freya L.; Astone, Nan M.; Sifakis, Frangiscos	2009	What are the consequences of relying upon self-reports of sexually transmitted diseases? Lessons learned about recanting in a longitudinal study	10.1016/j.jadohealth.2008.12.024
Study Aim	Fortenberry, J. Dennis	2009	Beyond validity and reliability: Meaning-in-context of adolescents' self-reports of sexual behavior	10.1016/j.jadohealth.2008.12.018
Study Aim	Ghosh, P.; Tu, W. Z.	2009	Assessing Sexual Attitudes and Behaviors of Young Women: A Joint Model with Nonlinear Time Effects, Time Varying Covariates, and Dropouts	10.1198/jasa.2009.0013
Study Aim	Giannotta, Fabrizia; Ciairano, Silvia; Spruijt, Rob; Spruijt-Metz, Donna	2009	Meanings of sexual intercourse for Italian adolescents	10.1016/j.adolescence.2008.05.009
Study Aim	Igartua, Karine; Thombs, Brett D.; Burgos, Giovani; Montoro, Richard	2009	Concordance and discrepancy in sexual identity, attraction, and behavior among adolescents	10.1016/j.jadohealth.2009.03.019
Study Aim	Roth, Marcus; Hammelstein, Philipp; Brähler, Elmar	2009	Towards a multi-methodological approach in the assessment of sensation seeking	10.1016/j.paid.2008.09.023
Study Aim	White, Stuart F.; Cruise, Keith R.; Frick, Paul J.	2009	Differential correlates to self-report and parent-report of callous-unemotional traits in a sample of juvenile sexual offenders	10.1002/bsl.911
Study Aim	Anwar, Mudassir; Sulaiman, Syed Azhar S.; Ahmadi, Keivan; Khan, Tahir M.	2010	Awareness of school students on sexually transmitted infections (STIs) and their sexual behavior: a cross-sectional study conducted in Pulau Pinang, Malaysia	10.1186/1471-2458-10-47
Study Aim	Corliss, Heather L.; Rosario, Margaret; Wypij, David; Wylie, Sarah A.; Frazier, A. Lindsay; Austin, S. Bryn	2010	Sexual orientation and drug use in a longitudinal cohort study of US adolescents	10.1016/j.addbeh.2009.12.019
Study Aim	Ibekwe, P. C.; Obuna, J. A.	2010	Awareness and practice of emergency contraception among university students in Abakaliki, Southeast Nigeria	N/A
Study Aim	Moore, Elya E.; Romaniuk, Helena; Olsson, Craig A.; Jayasinghe, Yasmin; Carlin, John B.; Patton, George C.	2010	The prevalence of childhood sexual abuse and adolescent unwanted sexual contact among boys and girls living in Victoria, Australia	10.1016/j.chiabu.2010.01.004
Study Aim	Ab Rahman, Azriani; Ab Rahman, Razlina; Ibrahim, Mohd Ismail; Salleh, Halim; Ismail, Shaiful Bahri; Ali, Siti Hawa; Muda, Wan Manan Wan; Ishak, Maizun; Ahmad, Amaluddin	2011	Knowledge of sexual and reproductive health among adolescents attending school in Kelantan, Malaysia	N/A
Study Aim	Anglewicz, Philip; Chintsanya, Jesman	2011	Disclosure of HIV status between spouses in rural Malawi	10.1080/09540121.2010.542130
Study Aim	Eastwick, Paul W.; Eagly, Alice H.; Finkel, Eli J.; Johnson, Sarah E.	2011	Implicit and explicit preferences for physical attractiveness in a romantic partner: A double dissociation in predictive validity	10.1037/a0024061
Study Aim	Langhaug, Lisa F.; Cheung, Yin Bun; Pascoe, Sophie J. S.; Chirawu, Petronella; Woelk, Godfrey; Hayes, Richard J.; Cowan, Frances M.	2011	How you ask really matters: randomised comparison of four sexual behaviour questionnaire delivery modes in Zimbabwean youth	10.1136/sti.2009.037374

Reason for Exclusion/ Inclusion	Author(s)	Year	Title	DOI
Study Aim	Onyeonoro, U. U.; Oshi, D. C.; Ndimele, E. C.; Chuku, N. C.; Onyemuchara, I. L.; Ezekwere, S. C.; Oshi, S. N.; Emelumadu, O. F.	2011	Sources of Sex Information and its Effects on Sexual Practices among In-school Female Adolescents in Osisioma Ngwa LGA, South East Nigeria	10.1016/j.jpag.2011.05.002
Study Aim	Shulman, Shmuel; Davila, Joanne; Shachar-Shapira, Lital	2011	Assessing romantic competence among older adolescents	10.1016/j.adolescence.2010.08.002
Study Aim	Asekun-Olarinmoye, E. O.; Dairo, M. D.; Abodurin, O. L.; Asekun-Olarinmoye, I. O.	2012	Practice and content of sex education among adolescents in a family setting in rural southwest Nigeria	10.2190/IQ.32.1.f
Study Aim	Durongritichai, Vanida	2012	Knowledge, attitudes, self-awareness, and factors affecting HIV/AIDS prevention among Thai university students	N/A
Study Aim	Jeffe, K. K.; Deterding, K.; Hüppe, M. K.; Jeffe, F.; Feyerabend, S.; Potthoff, A.; Manns, M. P.; Wedemeyer, H.	2012	Are teenagers aware of the risk of a hepatitis B virus infection as a sexually transmitted disease?	10.1007/s00112-011-2559-3
Study Aim	Meraz, Melissa García; García, Alejandra Salvador; Guzmán Saldaña, Rebeca Ma. Elena	2012	Actitudes hacia la transformación de la vida en pareja: Soltería, matrimonio y unión libre = Attitudes towards transformation of couple live: singlehood, marriage and cohabitation	N/A
Study Aim	Mullins, T. K.; Braverman, P. K.; Dorn, L. D.; Kollar, L. M.; Kahn, J. A.	2012	Adolescents' agreement to test for HIV when different testing methods are offered	10.1258/ijsa.2009.009035
Study Aim	Peter, Jochen; Valkenburg, Patti M.	2012	Do questions about watching Internet pornography make people watch Internet pornography? A comparison between adolescents and adults	10.1093/ijpor/edr052
Study Aim	Pullman, Lesleigh; Seto, Michael C.	2012	Assessment and treatment of adolescent sexual offenders: Implications of recent research on generalist versus specialist explanations	10.1016/j.chiabu.2011.11.003
Study Aim	Richter, Diana; Spangenberg, Lena; Matthes, Anke; Brähler, Elmar; Straußß, Bernhard	2012	Standardisierte Verfahren in der empirischen Sexualforschung—Ein Update = Standardized measures in empirical sex research—An update	10.1055/s-0031-1284040
Study Aim	Bergamini, M.; Cucchi, A.; Guidi, E.; Stefanati, A.; Bonato, B.; Lupi, S.; Gregorio, P.	2013	Risk perception of sexually transmitted diseases and teenage sexual behaviour: Attitudes towards in a sample of Italian adolescents	N/A
Study Aim	Grey, Jeremy A.; Robinson, Beatrice Bean E.; Coleman, Eli; Bockting, Walter O.	2013	A systematic review of instruments that measure attitudes toward homosexual men	10.1080/00224499.2012.746279
Study Aim	Inyang, M. P.; Inyang, O. P.	2013	Nigerian secondary school adolescents' perspective on abstinence-only sexual education as an effective tool for promotion of sexual health	10.12688/f1000research.2-86.v2
Study Aim	Mitchell, Kirstin R.; Mercer, Catherine H.; Ploubidis, George B.; Jones, Kyle G.; Datta, Jessica; Field, Nigel; Copas, Andrew J.; Tanton, Clare; Erens, Bob; Sonnenberg, Pam; Clifton, Soazig; Macdowall, Wendy; Phelps, Andrew; Johnson, Anne M.; Wellings, Kaye	2013	Sexual function in Britain: Findings from the third National Survey of Sexual Attitudes and Lifestyles (Natsal-3)	10.1016/S0140-6736(13)62366-1
Study Aim	Moreau, C.; Hall, K.; Trussell, J.; Barber, J.	2013	Effect of prospectively measured pregnancy intentions on the consistency of contraceptive use among young women in Michigan	10.1093/humrep/des421
Study Aim	Pai, Hsiang-Chu; Lee, Sheuan; Yen, Wen-Jiuan; Lee, Ming-Yung	2013	Testing of the factor structure of the Sexual Health Knowledge Measure with young adolescent Taiwanese girls	10.1080/07399332.2012.700357
Study Aim	Raheel, H.; Mahmood, M. A.; Binsaeed, A.	2013	Sexual practices of young educated men: Implications for further research and health education in Kingdom of Saudi Arabia (KSA)	10.1093/pubmed/fds055
Study Aim	Sim-Sim, M.; Viana, E.	2013	Validation of attitudes toward sex education scale (ATSES) in portuguese adolescents	10.1016/S0212-6567(13)70034-9
Study Aim	van Rosmalen-Nooijens, Karin A. W. L.; Prins, Judith B.; Vergeer, Marianne; Lo Wong, Sylvie H. Fo; Lagro-Janssen, Antoine L. M.	2013	"Young people, adult worries": RCT of an internet-based self-support method "Feel the ViBe" for children, adolescents and young adults exposed to family violence, a study protocol	10.1186/1471-2458-13-226
Study Aim	Whitfield, Clare; Jomeen, Julie; Hayter, Mark; Gardiner, Eric	2013	Sexual health information seeking: a survey of adolescent practices	10.1111/jocn.12192
Study Aim	Abajobir, Amanuel Alemu; Seme, Assefa	2014	Reproductive health knowledge and services utilization among rural adolescents in east Gojjam zone, Ethiopia: a community-based cross-sectional study	10.1186/1472-6963-14-138
Study Aim	Ballonoff Suleiman, Ahna; Brindis, Claire D.	2014	Adolescent School-Based Sex Education: Using Developmental Neuroscience to Guide New Directions for Policy and Practice	10.1007/s13178-014-0147-8

Reason for Exclusion/ Inclusion	Author(s)	Year	Title	DOI
Study Aim	Carrera-Fernández, María Victoria; Lameiras-Fernández, María; Rodríguez-Castro, Yolanda; Vallejo-Medina, Pablo	2014	Spanish adolescents' attitudes toward transpeople: proposal and validation of a short form of the Genderism and Transphobia Scale	10.1080/00224499.2013.773577
Study Aim	Degroote, S.; Vogelaers, D.; Liefhooghe, G.; Vermeir, P.; Vandijck, D. M.	2014	Sexual experience and HIV-related knowledge among Belgian university students: a questionnaire study	10.1186/1756-0500-7-299
Study Aim	Farih, Manal; Khan, Khalid; Della Freeth; Meads, Catherine	2014	Protocol study: sexual and reproductive health knowledge, information-seeking behaviour and attitudes among Saudi women: a questionnaire survey of university students	10.1186/1742-4755-11-34
Study Aim	Fentahun, Netsanet; Mamo, Abebe	2014	Risky sexual behaviors and associated factors among male and female students in Jimma Zone preparatory schools, South West Ethiopia: comparative study	10.4314/ejhs.v24i1.8
Study Aim	Jaruseviciene, L.; Valius, L.; Decat, P.; Meyer, S. de; Cordova, K.; Auquilla, N.	2014	Gender attitudes and sexual behavior: Cross-sectional study of bolivian and ecuadorian adolescents	10.1111/(ISSN)1743-6109
Study Aim	Monto, Martin A.; Supinski, Jessica	2014	Discomfort with homosexuality: a new measure captures differences in attitudes toward gay men and lesbians	10.1080/00918369.2014.870816
Study Aim	Norris, Anne E.; Torres-Thomas, Sylvia; Williams, Ellita T.	2014	Adapting cognitive interviewing for early adolescent Hispanic girls and sensitive topics	10.1891/1540-4153.12.3.111
Study Aim	Bauermeister, José A.; Pingel, Emily S.; Jadwin-Cakmak, Laura; Meanley, Steven; Alapati, Deepak; Moore, Michael; Lowther, Matthew; Wade, Ryan; Harper, Gary W.	2015	The use of mystery shopping for quality assurance evaluations of HIV/STI testing sites offering services to young gay and bisexual men	10.1007/s10461-015-1174-z
Study Aim	Dunkel, Curtis S.; Summerville, Lauren A.; Mathes, Eugene W.; Kesserling, Sean N.	2015	Using the California Q-sort measure of life history strategy to predict sexual behavioral outcomes	10.1007/s10508-014-0445-5
Study Aim	Eggers, Sander M.; Taylor, Myra; Sathiparsad, Reshma; Bos, Arjan E. R.; Vries, Hein de	2015	Predicting safe sex: Assessment of autoregressive and cross-lagged effects within the Theory of Planned Behavior	10.1177/1359105313512354
Study Aim	Hammarström, Sofia; Tikkanen, Ronny; Stenqvist, Karin	2015	Identification and risk assessment of Swedish youth at risk of chlamydia	10.1177/1403494815572722
Study Aim	Naigaga, Mpolampola D A S; Guttersrud, Øystein; Pettersen, Kjell S.	2015	Measuring maternal health literacy in adolescents attending antenatal care in a developing country - the impact of selected demographic characteristics	10.1111/jocn.12796
Study Aim	Phillips, Gregory 2nd; Ybarra, Michele L.; Prescott, Tonya L.; Parsons, Jeffrey T.; Mustanski, Brian	2015	Low Rates of Human Immunodeficiency Virus Testing Among Adolescent Gay, Bisexual, and Queer Men	10.1016/j.jadohealth.2015.06.014
Study Aim	Rahman, Azriani Abdul; Rahman, Razlina Abdul; Ismail, Shaiful Bahari; Ibrahim, Mohd Ismail; Ali, Siti Hawa; Salleh, Halim; Wan Muda, Wan Abdul Manan	2015	Factors associated with attitude toward premarital sexual activities among school-going adolescents in Kelantan, Malaysia	10.1177/1010539512449856
Study Aim	Rasmussen, Anna R.; Wohlfahrt-Veje, Christine; Renzy-Martin, Katrine Tefre de; Hagen, Casper P.; Tinggaard, Jeanette; Mouritsen, Annette; Mieritz, Mikkel G.; Main, Katharina M.	2015	Validity of self-assessment of pubertal maturation	10.1542/peds.2014-0793
Study Aim	Seto, Michael C.; Hermann, Chantal A.; Kjellgren, Cecilia; Priebe, Gisela; Svedin, Carl Göran; Långström, Niklas	2015	Viewing child pornography: prevalence and correlates in a representative community sample of young Swedish men	10.1007/s10508-013-0244-4
Study Aim	Turiho, Andrew Kampikaho; Muhwezi, Wilson Winston; Okello, Elialilia Sarikiaeli; Tumwesigye, Nazarius Mbona; Banura, Cecil; Katahoire, Anne Ruhweza	2015	Human Papillomavirus (HPV) Vaccination and Adolescent Girls' Knowledge and Sexuality in Western Uganda: A Comparative Cross-Sectional Study	10.1371/journal.pone.0137094
Study Aim	Babatunde, Oluwole Adeyemi; Ibirongbe, Demilade Olusola; Omede, Owen; Babatunde, Olubukola Oluwakemi; Durowade, Kabir Adekunle; Salaudeen, Adekunle Ganiyu; Akande, Tanimola Makanjuola	2016	Knowledge and use of emergency contraception among students of public secondary schools in Ilorin, Nigeria	10.11604/pamj.2016.23.74.8688
Study Aim	Brayboy, L. M.; Mills, B. L.; Sepolen, A. L.; Mezoian, T. J.; Wheeler, C.; Clark, M. A.	2016	Girl talk: A smartphone application to teach sexual health education to adolescent girls	10.1097/01.AOG.0000483336.51025.23
Study Aim	Cutbush, Stacey; Williams, Jason	2016	Teen dating violence, sexual harassment, and bullying among middle school youth: Examining measurement invariance by gender	10.1111/jora.12244

Reason for Exclusion/ Inclusion	Author(s)	Year	Title	DOI
Study Aim	Fagerlund, Monica; Ellonen, Noora	2016	Children's experiences of completing a computer-based violence survey: Finnish child victim survey revisited	10.1080/10538712.2016.1186769
Study Aim	Owonikoko, Kola M.; Bello-Ajao, Hajarah T.; Fawole, Adegboyega A.; Adeniji, Adetunji O.	2016	Determinants of sexual activities and contraceptive usage among adolescents in high schools in Ogbomoso, a semi-urban settlement in Nigeria	10.1515/ijamh-2015-0001
Study Aim	Rani, M.; Sheoran, P.; Kumar, Y.; Singh, N.	2016	Evaluating the effectiveness of pubertal preparedness program in terms of knowledge and attitude regarding pubertal changes among pre-adolescent girls	N/A
Study Aim	Susanto, T.; Rahmawati, I.; Wuryaningsih, E. W.; Saito, R.; Syahrul; Kimura, R.; Tsuda, A.; Tabuchi, N.; Sugama, J.	2016	Prevalence of factors related to active reproductive health behavior: a cross-sectional study Indonesian adolescent	10.4178/epih.e2016041
Study Aim	Vakilian, Katayon; Abbas Mousavi, Seyed; Keramat, Afsaneh; Chaman, Reza	2016	Knowledge, attitude, self-efficacy and estimation of frequency of condom use among Iranian students based on a crosswise model	10.1515/ijamh-2016-0010
Study Aim	Brayboy, Lynae M.; Sepolen, Alexandra; Mezoian, Taylor; Schultz, Lucy; Landgren-Mills, Benedict S.; Spencer, Noelle; Wheeler, Carol; Clark, Melissa A.	2017	Girl Talk: A Smartphone Application to Teach Sexual Health Education to Adolescent Girls	10.1016/j.jpag.2016.06.011
Study Aim	Chrisler, Alison J.	2017	Parents of lesbian, gay, and bisexual youth: Understanding parent reactions to coming out	10.1111/jftr.12194
Study Aim	Dewitte, M.; Schryver, M. de; Heider, N.; Houwer, J. de	2017	The Actual and Ideal Sexual Self Concept in the Context of Genital Pain Using Implicit and Explicit Measures	10.1016/j.jsxm.2017.03.246
Study Aim	Gilbert, Mark; Salway, Travis; Haag, Devon; Fairley, Christopher K.; Wong, Jason; Grennan, Troy; Uddin, Zhaida; Buchner, Christopher S.; Wong, Tom; Krajden, Mel; Tyndall, Mark; Shoveller, Jean; Ogilvie, Gina	2017	Use of GetCheckedOnline, a Comprehensive Web-based Testing Service for Sexually Transmitted and Blood-Borne Infections	10.2196/jmir.7097
Study Aim	Macapagal, K.; Coventry, R.; Arbeit, M. R.; Fisher, C. B.; Mustanski, B.	2017	"I Won't Out Myself Just to Do a Survey": Sexual and Gender Minority Adolescents' Perspectives on the Risks and Benefits of Sex Research	10.1007/s10508-016-0784-5
Study Aim	Nekuei, Nafisehsadat; Zirakidana, Akram; Kazemi, Ashraf; Beigi, Nastaran Mohhamad Ali; Alijanpoor, Masoomeh	2017	The Relationship Between Parental Abuse with Parental and Marital Roles Attitude in Girls	10.4103/1735-9066.205962
Study Aim	Priestley, Sharon, PHD; Lipps, Garth, PHD; Anderson, Patricia, PHD	2017	The Impact of Masculinity Ideologies and Conjugal Involvement on Sexual Risk-Taking among Young Jamaican Males	10.3149/jmh.1601.49
Study Aim	Barbour, W. L.; Rodgers, J. B.; Wang, H. E.; Donnelly, J. P.; Tapley, A. M.; Galbraith, J. W.	2018	Human Immunodeficiency Virus Risk Factors and Beliefs Reported by Families Presenting to a Pediatric Emergency Department	10.1097/PEC.0000000000000536
Study Aim	Barra, Steffen; Bessler, Cornelia; Landolt, Markus A.; Aebi, Marcel	2018	Testing the validity of criminal risk assessment tools in sexually abusive youth	10.1037/pas0000590
Study Aim	Brown, J. L.; Sales, J. M.; Sharp, C.; Cloete, J.; Lenka, M.; Rani, K.; Marime, P.; Ditlhare, I.; Moqolo, R.; Peterson, D.; Marais, L.	2018	Cultural consensus modelling to understand the reproductive health needs of South African adolescent girls	10.7196/SAJCH.2018.v12i2.1500
Study Aim	Dudeja, P.; Sindhu, A.; Shankar, P.; Gadekar, T.	2018	A cross-sectional study to assess awareness about menstruation in adolescent girls of an urban slum in western Maharashtra	10.1515/ijamh-2016-0079
Study Aim	Gerdes, Zachary T.; Alto, Kathleen M.; Jadaszewski, Stefan; D'Auria, Francisco; Levant, Ronald F.	2018	A content analysis of research on masculinity ideologies using all forms of the Male Role Norms Inventory (MRNI)	10.1037/men0000134
Study Aim	Hiremath, R. N.; Yadav, A. K.; Ghodke, Sandhya; Yadav, Jyoti	2018	Contraceptive use and its determinants amongst armed forces personnel	10.1016/j.mjafi.2016.12.007
Study Aim	Kilimnik, Chelsea D.; Pulverman, Carey S.; Meston, Cindy M.	2018	Methodologic Considerations for the Study of Childhood Sexual Abuse in Sexual Health Outcome Research: A Comprehensive Review	10.1016/j.sxmr.2017.11.006
Study Aim	Lamontagne, Erik; d'Elbée, Marc; Ross, Michael W.; Carroll, Aengus; Du Plessis, André; Loures, Luiz	2018	A socioecological measurement of homophobia for all countries and its public health impact	10.1093/eurpub/cky023
Study Aim	Patel, Pallavi; Puwar, Tapasvi; Shah, Neeta; Saxena, Deepak; Trivedi, Poonam; Patel, Krupali; Yasobant, Sandul; Fancy, Manish; Matela, Hema; Savaliya, Shital; Kalpana, Pachillu; Rana, Ritu	2018	Improving Adolescent Health: Learnings from an Interventional Study in Gujarat, India	10.4103/ijcm.IJCM_286_18
Study Aim	Tripathy, R. M.; Mohanty, S.; Panda, M.; Kar, M.	2018	Utilisation of adolescent health services under RMNCH+A in an urban slum of Berhampur, Odisha, India	10.7860/JCDR/2018/31753.11209

Reason for Exclusion/ Inclusion	Author(s)	Year	Title	DOI
Study Aim	Yépez-Tito, Paula; Ferragut, Marta; Blanca, María J.	2018	Prevalence and profile of sexting among adolescents in ecuador	10.1080/13676261.2018.1515475
Study Aim	Adimora, D. E.; Onwu, A. O.	2019	Socio-demographic factors of early sexual debut and depression among adolescents	10.4314/ahs.v19i3.39
Study Aim	Alsubaie, Ali Saad R.	2019	Exploring Sexual Behaviour and Associated Factors among Adolescents in Saudi Arabia: A Call to End Ignorance	10.2991/jegh.k.181210.001
Study Aim	Awang, Halimah; Low, Wah Yun; Tong, Wen Ting; Tan, Lih Yoong; Cheah, Whye Lian; Benedict Lasimbang, Helen; Mohd Hassan, Hamizah	2019	DIFFERENTIALS IN SEXUAL AND REPRODUCTIVE HEALTH KNOWLEDGE AMONG EAST MALAYSIAN ADOLESCENTS	10.1017/S0021932018000214
Study Aim	Berhane, Yemane; Worku, Alemayehu; Tewahido, Dagmawit; Fasil, Nebiyou; Gulema, Hanna; Tadesse, Amare W.; Abdelmenan, Semira	2019	Adolescent Girls' Agency Significantly Correlates With Favorable Social Norms in Ethiopia-Implications for Improving Sexual and Reproductive Health of Young Adolescents	10.1016/j.jadohealth.2018.12.018
Study Aim	Cairncross, Zoe F.; Ravindran, Saranyah; Yoganathan, Shaira; Dennis, Cindy-Lee; Enders, Joanne; Graves, Lisa; Mill, Catriona; Telner, Deanna; Brown, Hilary K.	2019	Measurement of preconception health knowledge: A systematic review	10.1177/0890117119835518
Study Aim	Corcoran, Jessica L.; Patrician, Patricia A.; Childs, Gwendolyn D.; Shirey, Maria R.	2019	What do we really know about adolescent sexual health education: A dimensional concept analysis // What Do We Really Know About Adolescent Sexual Health Education: A Dimensional Concept Analysis	10.1080/15546128.2019.1600446
Study Aim	Craig, Shelley L.; McInroy, Lauren B.; Eaton, Andrew David; Iacono, Gio; Leung, Vivian Wy; Austin, Ashley; Dobinson, Cheryl	2019	An Affirmative Coping Skills Intervention to Improve the Mental and Sexual Health of Sexual and Gender Minority Youth (Project Youth AFFIRM): Protocol for an Implementation Study	10.2196/13462
Study Aim	Delgado, C. Y.S.; Carrasco, J. D.R.; Sanchez, D. A.R.; Rodriguez, V. P.R.; Alvarado, J. M.P.; Carrasco, M. F.L.	2019	Descriptive study of the bio-psychosocial characteristics of adolescents in Unidad Educativa Herlinda Toral. Cuenca - Ecuador. 2018	10.30554/archmed.19.2.3285.2019
Study Aim	Handayani, O.O.K.; Wiranti, I.; Raharjo, B. B.; Nugroho, E.	2019	The reproduction health behavior of high school teenagers in semarang, indonesia	10.2174/1874944501912010309
Study Aim	Haruna, Hussein; Hu, Xiao; Chu, Samuel Kai Wah; Mellecker, Robin R.	2019	Initial Validation of the MAKE Framework: A Comprehensive Instrument for Evaluating the Efficacy of Game-Based Learning and Gamification in Adolescent Sexual Health Literacy	10.5334/aogh.1110
Study Aim	Jishala, M. I.; Kannan, P.; Kandasamy, K.; Rajagopal, S.; Ramanathan, S.	2019	Evaluating the effectiveness of mother's education in terms of knowledge, attitude, and practice regarding pubertal awareness among school-going prepubertal girls	10.5005/jp-journals-10016-1181
Study Aim	Kassie, B. A.; Yenus, H.; Berhe, R.; Kassahun, E. A.	2019	Prevalence of sexually transmitted infections and associated factors among the University of Gondar students, Northwest Ethiopia: A cross-sectional study	10.1186/s12978-019-0815-5
Study Aim	Koukounari, Artemis; Copas, Andrew J.; Pickles, Andrew	2019	A latent variable modelling approach for the pooled analysis of individual participant data on the association between depression and chlamydia infection in adolescence and young adulthood in the UK	10.1111/rssa.12387
Study Aim	Kusheta, S.; Bancha, B.; Habtu, Y.; Helamo, D.; Yohannes, S.	2019	Adolescent-parent communication on sexual and reproductive health issues and its factors among secondary and preparatory school students in Hadiya Zone, Southern Ethiopia: Institution based cross sectional study 11 Medical and Health Sciences 1117 Public Health and Health Services	10.1186/s12887-018-1388-0
Study Aim	Li, Ya-Huei; Mgbere, Osaro; Abughosh, Susan; Chen, Hua; Cuccaro, Paula; Smesny, Andrea; Essien, Ekere James	2019	Assessment of sexually transmitted disease/HIV risk among young African Americans: comparison of self-perceived and epidemiological risks utilizing ecodevelopmental theory	10.2147/HIV.S189482
Study Aim	Moltrecht, B.; Aymeric, S.; Sautiere, E.; Koenig, D.; Arnault, E.; Rusch, E.; Courtois, R.	2019	Emotional incest: Proposal of objective criteria for use with young people referred to therapeutic, educational and pedagogical institutes (ITEP)	10.1016/j.neurenf.2018.11.004
Study Aim	Morrison, Melanie A.; Bishop, C. J.; Morrison, Todd G.	2019	A Systematic Review of the Psychometric Properties of Composite LGBT Prejudice and Discrimination Scales	10.1080/00918369.2017.1422935
Study Aim	Olaoye, Titilayo; Agbede, Catherine	2019	Prevalence and personal predictors of risky sexual behaviour among in-school adolescents in the Ikenne Local Government Area, Ogun State, Nigeria	10.1515/ijamh-2019-0135
Study Aim	Patel, S. M.; Vernekar, S. P.; Desai, A. M.	2019	A study on the knowledge, attitude and practices regarding menstrual hygiene among adolescent girls in schools in a rural area of goa	10.7860/JCDR/2019/41251.12912
Study Aim	Svarrer, Rebekka O.; Rasmussen, Anna Lund; Lauszus, Finn Friis; Hammer, Anne	2019	No effect of human papillomavirus vaccination on sexual debut of school children	N/A

Reason for Exclusion/ Inclusion	Author(s)	Year	Title	DOI
Study Aim	Yakubu, Ibrahim; Garmaroudi, Gholamreza; Sadeghi, Roya; Tol, Azar; Yekaninejad, Mir Saeed; Yidana, Adadow	2019	Assessing the impact of an educational intervention program on sexual abstinence based on the health belief model amongst adolescent girls in Northern Ghana, a cluster randomised control trial	10.1186/s12978-019-0784-8
Study Aim	Yanez-Penunuri, L. Y.; Hidalgo-Rasmussen, C. A.; Chavez-Flores, Y. V.	2019	Systematic review of dating violence questionnaires in Ibero-America and evaluation of their measurement properties	10.1590/1413-81232018246.19612017
Study Aim	Abate, B. B.; Gelaw, K. H.; Fentaw, H.; Ashagire, M.; Mekash, T.	2020	Knowledge level and associated factors of reproductive health issues among secondary school students in Woldia town, Amhara, Ethiopia, 2019: A cross-sectional study	10.1155/2020/2515292
Study Aim	Abdullah, Fa'iza; Rahman, Nor Azlina A.; Muhammad, Noor Azimah; Zainuddin, N. Akmanidar; Samsudin, Suhaiza	2020	Association between social-cognitive factors and intention towards sexual activity among school-going late adolescents in kuantan, malaysia	10.1080/02673843.2020.1828111
Study Aim	Akther, N.; Begum, M.; Tasmin, T.; Imtiaz, K. S.; Alam, A. N.; Begum, A.; Begum, N.	2020	Awareness about reproductive health issues among the adolescent girls in a rural area of bangladesh	10.3329/bjms.v19i3.45876
Study Aim	Alsubaie, A.S.R.	2020	Examining HIV and stis related knowledge among male adolescents in Saudi Arabia	10.2174/1874613602014010027
Study Aim	Asal, I. S.; Sahib, M. S.; Hamza, M. S.; Kadhim Hussein Jassim, R. N.	2020	Female adolescents knowledge regarding reproductive health	10.37506/ijfmt.v14i3.10650
Study Aim	Ayele, Y. T.; Asemahagn, M. A.; Awoke, T.	2020	Prevention of Sexually Transmitted Infections and Associated Factors Among Night School Students in Bahir Dar City, Ethiopia	10.1177/1178633720927374
Study Aim	Ayton, Sarah Gabrielle; Pavlicova, Martina; Tamir, Hod; Abdool Karim, Quarraisha	2020	Development of a prognostic tool exploring female adolescent risk for HIV prevention and PrEP in rural South Africa, a generalised epidemic setting	10.1136/sextrans-2019-054067
Study Aim	Baokhumkong, C.; Leetongdee, S.	2020	Factors associated with the risk behaviors of sexually transmitted diseases among students in ubon ratchathani province, thailand	10.37506/ijphrd.v11i5.9404
Study Aim	Chaumaroeng, M.; Panza, A.	2020	Prevalence and factors associated with safe sex behaviors among adolescent vocational students in Nakhon Ratchasima Province, Thailand	10.1108/JHR-03-2019-0061
Study Aim	Cimpian, Joseph R.; Timmer, Jennifer D.	2020	Mischievous responders and sexual minority youth survey data: A brief history, recent methodological advances, and implications for research and practice	10.1007/s10508-020-01661-7
Study Aim	Dagnachew Adam, N.; Demissie, G. D.; Gelagay, A. A.	2020	Parent-Adolescent Communication on Sexual and Reproductive Health Issues and Associated Factors among Preparatory and Secondary School Students of Dabat Town, Northwest Ethiopia	10.1155/2020/4708091
Study Aim	Leon, C. M.; Aizpurua, E.	2020	DO SEXIST ATTITUDES PERSIST IN COLLEGE STUDENTS? AN ANALYSIS OF ITS PREVALENCE, PREDICTORS, AND GENDER DIFFERENCES	10.5944/educXX1.23629
Study Aim	Macdonald, David W.; Grossoehme, Daniel H.; Mazzola, Amanda; Pestian, Teresa; Schwartz, Scott B.	2020	Oral Sex Knowledge and Experience of Transgender Youth: An Opportunity for Dental Education	10.21815/JDE.019.193
Study Aim	Maina, Beatrice W.; Orindi, Benedict O.; Sikweyiya, Yandisa; Kabiru, Caroline W.	2020	Gender norms about romantic relationships and sexual experiences among very young male adolescents in Korogocho slum in Kenya	10.1007/s00038-020-01364-9
Study Aim	Maina, Beatrice W.; Orindi, Benedict O.; Sikweyiya, Yandisa; Kabiru, Caroline W.	2020	'Gender norms about romantic relationships and sexual experiences among very young male adolescents in Korogocho slum in Kenya': Correction	10.1007/s00038-020-01512-1
Study Aim	Mamani-Benito, O.; Pinto-Cahuapaza, J.; Nunez-Vargas, J.; Chani, F. Q.; Ordono, S. M.; Huamantuco, R. V.	2020	Efficacy of the educational program "Mi sol" for the prevention of child sexual abuse in children in the city of Juliaca, Peru	10.17162/au.v10i2.452
Study Aim	Morrison, A.; Olezeski, C.; Cron, J.; Kallen, A. N.	2020	A Pilot Study to Assess Attitudes Toward Future Fertility and Parenthood in Transgender and Gender Expansive Adolescents	10.1089/trgh.2019.0075
Study Aim	Oniyangi, S. O.; Tosin, J.A.Q.; Babangida, U. I.; Getso, A. M.; Helen, S.	2020	Prevalence of premarital sex among adolescents in kulende, sango in ilorin south local governmrnt area, Kwara state, Nigeria	10.37506/v11/i1/2020/ijphrd/193946
Study Aim	Rodriguez-Dominguez, C.; Perez-Moreno, P. J.; Duran, M.	2020	Cyber dating violence: A Review of Its Research Methodology	10.6018/analesps.370451
Study Aim	Santisouk, Phonevilai; Hansana, Visanou; Thanh Huong, Nguyen	2020	Pregnancy health literacy among teenagers in Kaysone district, Savannakhet Province, Lao PDR	10.1080/16549716.2020.1791412
Study Aim	Schoeps, K.; Peris Hernández, M.; Garaigordobil, M.; Montoya-Castilla, I.	2020	Risk factors for being a victim of online grooming in adolescents	10.7334/psicothema2019.179

Reason for Exclusion/ Inclusion	Author(s)	Year	Title	DOI
Study Aim	Sekera, J. C.; Preis, J.; Pazdiora, P.	2020	Health literacy on hiv infection among adolescents in the czech republic: A case study of two czech cities plzen and ceské budejovice	N/A
Study Aim	Soliman, Hussein Hassan; Abdelmonem, Amal Abdelmordi; Koran, Jennifer	2020	Adolescents' perception of the threat of sexual harassment: The development of an index	10.1080/10538712.2019.1667469
Study Aim	Stewart, Kelly E.	2020	Preventing child sexual abuse and juvenile offending through parental monitoring	10.15760/etd.6858
Study Aim	Sukanya, L.; Baskaran, R.	2020	Knowledge, attitude and practices regarding menstrual hygiene among adolescent school girls in Thandalam, Tamil Nadu	10.26452/ijrps.v11iSPL4.4358
Study Aim	Tesema, Desalew; Tamirat, Meseret; Tadele, Afework	2020	Sexual behaviors and its association with life skills among school adolescents of Mettu town, South West Ethiopia: A school-based cross-sectional study	10.1177/2050312120940545
Study Aim	Thompson, Ashley E.; Moore, Elle A.; Haedtke, Kassie; Karst, Aaron T.	2020	Assessing Implicit Associations with Consensual Non-monogamy Among U.S. Early Emerging Adults: An Application of the Single-Target Implicit Association Test	10.1007/s10508-020-01625-x
Study Aim	Zong, Chen; Donovan, Courtney; Fuchs, Taryn	2020	The Development and Validation of Teen Beliefs on Relationship Abuse Measure (TBRAM)	10.1177/0886260520976220
Study Aim	Agbede, C. O.; Ekeanyanwu, U. C.	2021	An outcome of educational intervention on the menstrual hygiene practices among school girls in Ogun State, Nigeria: a quasi-experimental study	10.11604/pamj.2021.40.214.30601
Study Aim	Allison, Bianca A.; Walters, Elizabeth M.; Butler, Benjamin W.; Perry, Martha F.	2021	A Clinic-Based Quality Improvement Initiative to Increase Screening for Gonorrhea and Chlamydia in Adolescents	10.1016/j.jcjq.2021.04.006
Study Aim	Ames, Allison J.; Leventhal, Brian C.	2021	Application of a Longitudinal IRTree Model: Response Style Changes Over Time	10.1177/10731911211042932
Study Aim	Ames, Allison J.; Myers, Aaron J.	2021	Explaining Variability in Response Style Traits: A Covariate-Adjusted IRTree	10.1177/0013164420969780
Study Aim	Anderson, R. E.; Namie, E.M.C.; Michel, P. K.; Delahanty, D. L.	2021	Study Title-Based Framing Effects on Reports of Sexual Violence and Associated Risk Factors in College Students	10.1177/08862605211016349
Study Aim	Aragie, T. G.; Abate, B. B.	2021	Utilization of Reproductive Health Services and Associated Factors among Secondary School Students in Woldia Town, Northeast Ethiopia	10.1155/2021/2917874
Study Aim	Bahari, Roza; Amin Shokravi, Farkhondeh; Anosheh, Monireh; Moridi, Maryam	2021	Effect of a health education program on puberty knowledge among visually impaired female adolescent students	10.47176/mjiri.35.74
Study Aim	Barbee, Anita P.; Antle, Becky; Langley, Cheri; Cunningham, Michael R.; Whiteside, Danielle; Sar, Bibhuti K.; Archuleta, Adrian; Karam, Eli; Borders, Kevin	2021	How to ensure fidelity in implementing an evidence based teen pregnancy prevention curriculum	10.1016/j.childyouth.2021.106175
Study Aim	Calatrava, Maria; Beltramo, Carlos; Osorio, Alfonso; Rodríguez-González, Martiño; Irala, Jokin de; Lopez-Del Burgo, Cristina	2021	Religiosity and Sexual Initiation Among Hispanic Adolescents: The Role of Sexual Attitudes	10.3389/fpsyg.2021.715032
Study Aim	Edwards-Rowlands, J.; Sakharkar, V.; Frankson, M.; Pinder-Butler, S.; Conliffe, C.; Laville, E.	2021	Evaluating the social determinants of teenage pregnancy in The Bahamas	10.1111/1471-0528.20-16715
Study Aim	Harling, Guy; Bountogo, Mamadou; Sié, Ali; Bärnighausen, Till; Lindstrom, David P.	2021	Nonverbal Response Cards Reduce Socially Desirable Reporting of Violence Among Adolescents in Rural Burkina Faso: A Randomized Controlled Trial	10.1016/j.jadohealth.2020.09.006
Study Aim	Kalliath, J. D.; Gnanaselvam, N. A.; Pinto, N. X.; Chirayath, M.; Ramesh, N.	2021	Empowerment status of school-going adolescents in Anekal Taluk of Bengaluru District	10.4103/jss.JSS_39_20
Study Aim	Langerman, S. D.; Badolato, G. M.; Goyal, M. K.	2021	Attitudes toward electronic sexual health assessments among adolescents in the emergency department	10.1097/PEC.0000000000001947
Study Aim	Lismidiati, Wiwin; Emilia, Ova; Widyawati, Widyawati	2021	Human Papillomavirus (HPV) Health Savings as an Alternative Solution: HPV Vaccination Behavior in Adolescents	10.31557/APJCP.2021.22.2.471
Study Aim	Liyeh, T. M.; Goshu, Y. A.; Belay, H. G.; Tasew, H. A.; Mihiretie, G. N.; Ayalew, A. B.	2021	Youth Reproductive Health Service Utilization and Associated Factors among Amhara Region Female Night Students, Ethiopia	10.1155/2021/6640219
Study Aim	McCarey, Catherine; Viviano, Manuela; Yaron, Michal	2021	FertiSTAT: A Potential Tool for Adolescent Sexual Health	10.1016/j.jpag.2021.04.007
Study Aim	Ogwunga, Chukwunonyerem C.; Anyadoh-Nwadike, Sylvia O.; Ahumibe, Nkenna C.; Nwakwasi, Emmanuel U.	2021	Knowledge and Attitude of Female Students of Tertiary Institutions in Imo State, Nigeria Towards Cervical Cancer and Its Screening	10.1007/s10900-020-00892-y
Study Aim	Seff, I.; Falb, K.; Yu, G.; Landis, D.; Stark, L.	2021	Gender-equitable caregiver attitudes and education and safety of adolescent girls in South Kivu, DRC: A secondary analysis from a randomized controlled trial	10.1371/journal.pmed.1003619

Reason for Exclusion/ Inclusion	Author(s)	Year	Title	DOI
Study Aim	Thompson, Erika L.; Zhou, Zhengyang; Garg, Ashvita; Rohr, Danielle; Ajoku, Brittany; Spence, Emily E.	2021	Evaluation of a School-Based Child Physical and Sexual Abuse Prevention Program	10.1177/1090198120988252
Study Aim	Ward-Peterson, Melissa; Fennie, Kristopher; Baird, Sarah; Coxe, Stefany; Trepka, Mary Jo; Madhivanan, Purnima	2021	Multilevel influences of women's empowerment and economic resources on risky sexual behaviour among young women in Zomba district, Malawi	10.1017/S0021932020000590
Study Aim	Becker, Tara; Chin, Marshall; Bates, Nancy	2022	Measuring Sex, Gender Identity, and Sexual Orientation	10.17226/26424
Study Aim	Carter, Jarvis W. [JR]; Salabarría-Peña, Yamir; Fields, Errol L.; Robinson, William T.	2022	Evaluating for health equity among a cluster of health departments implementing PrEP services	10.1016/j.evalprogplan.2021.101981
Study Aim	Vongxay, V.; Thongmixay, S.; Stoltenborg, L.; Inthapanyo, A.; Sychareun, V.; Chaleunvong, K.; Rombout Essink, D.	2022	Validation of the Questionnaire on Sexual and Reproductive Health Literacy for Adolescents Age 15 to 19 Years in Lao People's Democratic Republic	10.3928/24748307-20220207-01
Year of OMI-Development (Other)	Somers, Cheryl L.; Johnson, Stephanie A.; Sawilowsky, Shlomo S.	2002	A measure for evaluating the effectiveness of teen pregnancy prevention programs	10.1002/pits.10023
Year of OMI-Development (Other)	Canivez, Gary L.; Somers, Cheryl L.	2003	The sexual communication scale: a measure of frequency of sexual communication between parents and adolescents	N/A
Year of OMI-Development (Other)	Nagy, Stephen; Watts, Graham F.; Nagy, M. Christine	2003	Scales measuring psychosocial antecedents of coital initiation among adolescents in a rural southern state	10.2466/PR0.92.3.981-990
Year of OMI-Development (Other)	Kahn, Jessica A.; Huang, Bin; Austin, S. Bryn; Aweh, Gideon N.; Colditz, Graham A.; Frazier, A. Lindsay	2004	Development of a scale to measure adolescents' beliefs and attitudes about postponing sexual initiation	10.1016/j.jadohealth.2004.02.010
Year of OMI-Development (Other)	Farmer, G. Lawrence; McMahon, Sarah	2005	Scale for the Identification of Acquaintance Rape Attitudes: Reliability and factorial invariance	10.1300/J137v11n03_11
Year of OMI-Development (Other)	Lindfors, K.; Elovainio, M.; Sinkkonen, J.; Aalberg, V.; Vuorinen, R.	2005	Construct validity of the offer self-image questionnaire and its relationship with self-esteem, depression, and ego development	10.1007/s10964-005-5769-y
Year of OMI-Development (Other)	Teten, Andra L.; Hall, Gordon C. Nagayama; Pacifici, Caesar	2005	Validation of Acceptance of Coercive Sexual Behavior (ACSB): a multimedia measure of adolescent dating attitudes	10.1177/1073191105275042
Year of OMI-Development (Other)	Hutchinson, M. Katherine	2007	The Parent-Teen Sexual Risk Communication Scale (PTSRC-III): instrument development and psychometrics	10.1097/00006199-200701000-00001
Year of OMI-Development (Other)	Hutchinson, M. Katherine; Montgomery, Arlene J.	2007	Parent communication and sexual risk among African Americans	10.1177/0193945906297374
Year of OMI-Development (Other)	Lescano, Celia M.; Hadley, Wendy S.; Beausoleil, Nancy I.; Brown, Larry K.; D'eramo, Domenic; Zimskind, Abigail	2007	A brief screening measure of adolescent risk behavior	10.1007/s10578-006-0037-2
Year of OMI-Development (Other)	Bourdeau, Beth; Thomas, Volker K.; Long, Janie K.	2008	Latino sexual styles: Developing a nuanced understanding of risk	10.1080/00224490701845185
Year of OMI-Development (Other)	Heath, Lance; Euvrard, George	2008	The development of the Sexual Responsiveness Scale for adolescents and young adults: Putting a definition of sexual orientation to the test	10.1177/008124630803800404
Year of OMI-Development (Other)	Massey, Sean G.	2009	Polymorphous prejudice: Liberating the measurement of heterosexuals' attitudes toward lesbians and gay men	10.1080/00918360802623131

Reason for Exclusion/ Inclusion	Author(s)	Year	Title	DOI
Year of OMI-Development (Other)	Onya, Hans; Aarø, Leif Edvard; Madu, Sylvester N.	2009	Social outcome expectations regarding delayed sexual debut among adolescents in Mankweng, South Africa	10.1177/1403494808091344
Year of OMI-Development (Other)	Chen, Angela Chia-Chen; Morrison-Beedy, Dianne; Han, Chong-Suk	2010	Assessing linguistic and cultural equivalency of two Chinese-version sexual health instruments among Chinese immigrant youth	10.1016/j.pedn.2009.01.004
Year of OMI-Development (Other)	Jaruseviciene, Lina; Meyer, Sara de; Decat, Peter; Zaborskis, Apolinaras; Degomme, Olivier; Rojas, Mildrett; Arnold Hagens, Salazar; Auquilla, Nancy; Vega, Bernardo; Gorter, Anna C.; Orozco, Miguel; Lazarus, Jeffrey V.	2014	Factorial validation of the Attitudes toward Women Scale for Adolescents (AWSA) in assessing sexual behaviour patterns in Bolivian and Ecuadorian adolescents	10.3402/gha.v7.23126
Year of OMI-Development (Other)	Ubillos, Silvia; Goiburu, Eider; Puente, Alicia; Pizarro, Juan-Pablo	2016	Adaptation and validation of the Double Standard Scale in Basque adolescents = Adaptación y validación de la Escala de Doble Estándar en adolescentes vascas-os	10.1080/02134748.2016.1152683
Year of OMI-Development (Other)	Thoma, Brian C.	2017	Parental Monitoring Among Young Men Who Have Sex With Men: Associations With Sexual Activity and HIV-Related Sexual Risk Behaviors	10.1016/j.jadohealth.2017.03.004
Year of OMI-Development (Other)	Ubillos-Landa, S.; Goiburu-Moreno, E.; Puente-Martinez, A.; Pizarro-Ruiz, J. P.; Echeburua-Odriozola, E.	2017	Assessment of Distorted Thoughts About Women and Violence of Basque-speaking Secondary School Students	10.1387/RevPsicodidact.16124
Year of OMI-Development (Other)	Guzzo, Karen Benjamin; Hayford, Sarah R.; Lang, Vanessa Wanner; Wu, Hsueh-Sheng; Barber, Jennifer; Kusunoki, Yasamin	2019	Dimensions of Reproductive Attitudes and Knowledge Related to Unintended Childbearing Among U.S. Adolescents and Young Adults	10.1007/s13524-018-0747-7
Year of OMI-Development (Other)	Sicilia, Alvaro; Alcaraz-Ibáñez, Manuel; Granero-Gallegos, Antonio; Lirola, María-Jesús; Burgueño, Rafael	2020	Psychometric properties of the Objectified Body Consciousness Scale (OBCS) in Spanish preadolescents	10.1007/s11199-019-01043-x
Year of OMI-Development (Other)	Kamke, Kristyn; Widman, Laura; Javidi, Hannah	2021	The multidimensionality of adolescent girls' gender attitudes	10.1007/s12147-021-09288-1
Year of OMI-Development (Other)	Rancher, Caitlin; McDonald, Renee; Kamata, Akihito; Jackson, Mindy; Jouriles, Ernest N.	2022	Self-blame in adolescents who have been sexually abused: Factor structure and differential correlates of abuse-specific and global measures	10.1177/10731911211027632
