# Supplementary material for: A COSMIN Systematic Review of Sexual Health Literacy Self-Report Measures for Adolescents
Source: Arch Sex Behav. 2025 Jun 6;54(5):1737–68. doi: 10.1007/s10508-025-03142-1 (PMC12162768; doi:10.1007/s10508-025-03142-1)
Supplement: Supplementary file 5 — Supplementary file5 (PDF 188 KB) [file 10508_2025_3142_MOESM5_ESM.pdf]

Characteristic of the included OMI-studies

OMI	Study	Study-type	Country	Population[a] description, sample size, age range, mean age±SD, % female		
				**Design**	**Pilot**	**Validation[b]**
Adolescent Health Attitude and Behavior Survey (AHABS)	Reininger et al. (2003)	DV	USA	NA	High School Students<br>*N*=755<br>Grade 9-12	High School Students<br>*N*=4368<br>14-17y, *Mdn*=15y<br>53%f
The Adolescent Masculinity Ideology in Relationships Scale (AMIRS)	Chu et al. (2005)	DV	USA	Adolescent boys<br>*N*=65<br>12-18y	Adolescent boys<br>*N*=34<br>13-15y, *M*=14y	7th grade boys<br>*N*=114<br>10-14y, *M*=12.2y
					Adolescent boys<br>*N*=27<br>14-17y, *M*=14.5y	8th grade boys<br>*N*=133<br>12-15y, *M*=13.5y
						High school boys<br>*N*=31<br>15-18y, *M*=16.8y
Untitled	Stevens-Simon et al. (2005)	DV	USA	NA	Adolescent females<br>*N*=22	Adolescent females<br>*N*=351<br>10-19y, *M*=16.4y±1.5y
Relational Entitlement and Proprietariness Scale (REPS)	Hannawa et al. (2006)	DV	USA	Hispanic undergraduate students<br>*N*=103	NA	Undergraduate students<br>*N*=279<br>18-39y, *M*=18.92y±1.98y<br>51.3%f
The Cognitive Susceptibility Index	L'Engle et al. (2006)	DV	USA	Middle school students<br>*N*=6 focus groups<br>Grade 7	NA	Middle school students<br>*N*=1017<br>12-15y, *M*=13.7y<br>49.6%f
The Sexual Self-Concept Inventory (SSCI)	O'Sullivan et al. (2006)	DV	USA	Girls<br>*N*=68<br>12-14y	unclear	Girls<br>*N*=180<br>12-14y, *M*=13.1y*±0.73y*

OMI	Study	Study-type	Country	Population[a] description, sample size, age range, mean age±SD, % female		
				**Design**	**Pilot**	**Validation[b]**
The Measure of Adolescent Heterosocial Competence (MAHC)	Grover et al. (2007)	P	USA	NA	NA	NA
	Grover et al. (2005)	DV		Adolescents<br>*N*=150*<br>14-19y<br>55,33%f*	Adolescents<br>*N*=198*<br>14-19y<br>68.18%f*	Adolescents<br>*N*=208*<br>14-18y, *M*=15.96y±1.18y<br>50%f*
				Adolescents<br>*N*=157<br>14-19y<br>45.78%f*		
Untitled	Roye et al. (2007)	D	USA	NA	Black and Latina teenage women	NA
The HIV Knowledge Questionnaire for Adolescent Girls (HIV-KQ AG)	Volpe et al. (2007)	DV	USA	NA	NA	Single sexually active adolescent girls<br>*N*=62<br>15-19y, *M*=17.3y±1.4y
Untitled	Deardorff et al. (2008)	DV	USA	Sexually active Latino youth<br>*N*=55<br>16-22y	*N*=42	Sexually active Latino youth<br>*N*=694<br>16-22y, *M*=18.47y±1.65y<br>61%f
				Sexually active Latino youth<br>*N*=24<br>16-22y		
The Parent-Adolescent Communication Scale (PACS)	Sales et al. (2008)	DV	USA	African American female adolescents<br>*N*= 40<br>14-18y	African American female adolescents<br>*N*= 15<br>14-18y	Single<br>sexually active african American female adolescents<br>*N*=522<br>14-18y, *M*=16y±1.2y
The Worry About Sexual Outcomes (WASO) Scale	Sales et al. (2009)	DV	USA	African-American adolescent females<br>N=40	Sexually active African-American adolescent females<br>*N*=15<br>14–18y	Heterosexually active African-American adolescent females<br>*N*=522<br>14-18y, *M*=16y±1.2y

OMI	Study	Study-type	Country	Population[a] description, sample size, age range, mean age±SD, % female		
				**Design**	**Pilot**	**Validation[b]**
Reproductive Health Scale (RHS)	Saydam et al. (2010)	DV	Turkey	NA	Turkish university students<br>*N*=15<br>17-30y	Turkish university students<br>*N*=668<br>17-30y, *M*=18.9±1.6y<br>44.3%f
Adolescent Clinical Sexual Behavior Inventory-Self-Report (ACSBI-S)	Friedrich et al. (2004)	DV	USA	NA	Teenagers receiving inpatient treatment<br>*N*=23	Teenagers<br>*N*=174<br>12-18y, *M*=15.0y±1.4y<br>53.4%f
	Wherry et al. (2009)	V		NA	NA	Teenagers<br>*N*=141<br>12-19y, *M*=15.11±1.4y<br>55%f
An HIV/AIDS knowledge scale for adolescents	(Mũkoma et al., 2009)	DV	South Africa<br>Norway<br>Tanzania	NA	Students<br>*N*≈150<br>"Grades of interest"	High school and primary school students<br>*N*=217<br>Grade 5-6
						High school and primary school students<br>*N*=295<br>Grade 8
						High school and primary school students<br>*N*=120<br>Grade 8
	Aarø et al. (2011)	V		NA	NA	Students<br>*N*=15782<br>*M*=13.5y, 84.8%=12-15y<br>51.5%f
Adolescent Sexual Expectancies Scale (ASEXS)	Bourdeau et al. (2011)	DV	USA	NA	NA	Youth<br>*N*=1105<br>10-17y, *M*=14.1y

OMI	Study	Study-type	Country	Population[a] description, sample size, age range, mean age±SD, % female		
				Design	Pilot	Validation[b]
Untitled	Buhi et al. (2011)	DV	USA	NA	NA	Texas middle school youth, Wave 1<br>*N*=451<br>Grade 7-8<br>63.1%f
						Texas middle school youth, Wave 2<br>*N*=448, *M*=13.19y±0.745y<br>59.3%f
Sexual Motivations Scale – Revised (SMS-R) & Motivations Against Sex Questionnaire (MASQ)	Patrick et al. (2011)	DV	USA	NA	NA	High school students<br>*N*=1653<br>17-20y, *M*=17.99y±0.73y<br>58.4%f*
The Sexual Attitudes and Experiences Scale (SAES)	Tobin (2011)	DV	USA	NA	Undergraduate college students<br>*N*=202<br>at least 17y, 62%=17-19y<br>52%f	Undergraduate college students<br>*N*=455<br>at least 17y, 52%=17-19y<br>65%f
The Perceived Heterosexism Scale (PHS) & The Preoccupation with Disclosure of Parents' Sexual Orientation Scale (PDPSOS)	Vyncke et al. (2011)	DV	Canada	NA	NA	Adolescent children of lesbian mothers<br>*N*=64<br>12-18y, *M*=15.43y±1.71y<br>55%f*
The Adolescent Attitudes to Abortion Scale (AAA Scale)	Skinner et al. (2008)	P	Australia	Sexually active adolescent females<br>N=68<br>14-19y, Mdn=17y	NA	NA
	Skinner et al. (2009)	P		Sexually active adolescent females<br>N=68<br>14-19y, Mdn=17y	NA	NA
	Hendriks et al. (2012)	DV		NA	NA	Adolescents<br>N=406<br>12-19y<br>50%f

OMI	Study	Study-type	Country	Population[a] description, sample size, age range, mean age±SD, % female		
				**Design**	**Pilot**	**Validation[b]**
The Jamaican Maternal Sexual Role Modelling (Jamaican MSRM) Questionnaire	Hutchinson et al. (2007)	P	USA<br>Jamaica	NA	NA	NA
	Hutchinson, Kahwa, et al. (2012)	P		Jamaican female adolescents<br>*N*=46<br>14-18y, *M*=16.4y	NA	NA
	Hutchinson, Smith, et al. (2012)	DV		NA	NA	Jamaican female adolescents<br>*N*=209<br>*M*=15.1y±2.1y
The Relationship Options Survey (ROS)	Lusczakoski & Rue (2012)	DV	USA	NA	NA	Young Adults<br>*N*=201<br>18-19y<br>60%f
The Homophobic Bullying Scale	Prati (2012)	DV	Italy	unclear	Italian high school students<br>*N*=104	Italian public high school students<br>*N*=863<br>15-22y, *M*=17.26y±1.59y<br>60.7%f
The Comfort with Sexual Matters for Young Adolescents scale (CWSMYA)	Rye et al. (2012)	DV	Canada	NA	NA	Girls<br>*N*=883, *M*=12.5y<br><br>University students<br>*N*=55<br>18-20y, *M*=18y, SD<1y<br>70.9%f*
Alcohol and Sexual Consent Scale	Ward et al. (2012)	DV	USA	NA	NA	First year students<br>*N*=462, *M*=18.26y±1.53y
The Social Dating Goals Scale-Revised (SDGS-R)	Zimmer-Gembeck et al. (2012)	DV	Canada	*N*=10<br>19-22y<br>50%f	NA	Late Adolescents<br>*N*=121<br>16-19y
The Contraceptive Behavior Scale (CBS)	Wang et al. (2013)	DV	Taiwan	Sexually active female adolescents without experience of unintended pregnancy<br>*N*=3	Sexually active female adolescents<br>*N*=3	Female students<br>*N*=2325<br>15-19y, *M*=17.24y±0.86y

OMI	Study	Study-type	Country	Population[a] description, sample size, age range, mean age±SD, % female		
				Design	Pilot	Validation[b]
The Attitudes Toward Condom Use Scale (ATCUS)	Masa & Chowa (2014)	DV	USA<br>South Africa	NA	Ghanaian junior high school students<br>*N*=51<br><br>students<br>*N*=20	Ghanaian youth<br>*N*=6252, *M*=15.33y<br>51%f
Scale of Knowledge about Sexually Transmitted Infections	Nelas et al. (2014)	DV	Portugal	NA	NA	Adolescents<br>*N*=840<br>Grade 9
Measurement of Bystander Intervention in Bullying and Sexual Harassment	Nickerson et al. (2014)	DV	USA	NA	NA	High school students<br>*N*=662<br>13-20y, *M*=16.11y±1.28y
Maternal Health Literacy Scale (MaHeLi Scale)	Guttersrud et al. (2015)	DV	Norway	NA	NA	Pregnant adolescents<br>*N*=384<br>15-19y
Untitled	Mushwana et al. (2015)	DV	South Africa<br>Canada	NA	NA	Female adolescents<br>*N*=147<br>10-20y, 17.8%=10-15y, 82.2%=16-20y
Cyberdating Q_A	Sánchez et al. (2015)	DV	Spain	Secondary school students<br>*N*=16<br>14-17y<br>50%f*	First-year teacher-training university students<br>*N*=90<br>*M*=18.9y±0.88y	Adolescents and young adults<br>*N*=626<br>12-21y, *M*=15.13y±1.34y<br>48.6%f*
Sexual Health Questionnaire	Acharya et al. (2016)	DV	UK	Pupils	Pupils<br>*N*=24<br>14-18y,<br>*M*=15.75y±1.07y<br>91.67%f*	Secondary school pupils (Nepal)<br>*N*=259<br>*M*=15.38y±1.00y<br>56.37%f
The Sexting Motivations Questionnaire (SMQ)	Bianchi et al. (2016)	DV	Italy	NA	NA	Total<br>*N*=509<br>13-35y, *M*=21.4y±4.6y<br>63.7%f<br>(Subgroups: Adolescents & Young adults)

OMI	Study	Study-type	Country	Population[a] description, sample size, age range, mean age±SD, % female		
				Design	Pilot	Validation[b]
Sexual Self-Concept (SSC) Scale	Biney (2016)	DV	Ghana	Adolescents<br>*N*=50<br>12-19y<br>46%f*	NA	Adolescents<br>*N*=196<br>12-19y, 34.7%=12-14y, 33.7%=15-17y, 31.6%=18-19y<br>52%f*
Untitled	Jerman et al. (2015)	DV	USA	Hispanic Adolescents	Adolescents from the target population	Hispanic students<br>*N*=86<br>15-17y, *M*=15.58y*±0.54y*<br>48.8%f<br><br>Hispanic students<br>*N*=202<br>13-16y, *M*=14.14y*±0.52y*<br>50%f<br><br>Hispanic students<br>*N*=291<br>13-16y, *M*=14.19y*±0.58y*<br>54.6%f
Measures of Adolescents' Attitudes about Sexual Relationship Rights	Berglas et al. (2017)	DV	USA	Youth<br>*N*=16	Students in the target communities<br>N>700<br>Grade 9	Students<br>*N*=655<br>9%≤13y, 90%=14-15y, 1%≥16y<br>51%f
Scale for the Assessment of Sexual Standards among Youth (SASSY)	Emmerink et al. (2017)	DV	Netherlands	NA	NA	Heterosexual adolescents<br>*N*=465<br>16-20y, *M*=18.08y±1.34y<br>54.8%f
Condom Use Barriers Scale for Adolescents (CUBS-A)	Escribano et al. (2017)	DV	Spain	NA	Small sample<br>*N*=10<br>48.5%f*	Adolescents<br>*N*=629<br>13-18y, *M*=15.17y±1.09y
Attitudes Toward Sex Education (ATSES)	Sim-Sim & Viana (2017)	DV	Portugal	NA	Students<br>*N*=15	Students<br>*N*=186<br>12-18y, *M*=14y±1.51y<br>48.1%f*
The Makeup Questionnaire (MUQ)<br>The Sexualized Clothing Questionnaire (SCQ)	Smith et al. (2017)	DV	USA	Undergraduate women<br>*N*=27	NA	First-year college women<br>*N*=403<br>18-32y, *M*=18.79y±1.14y

OMI	Study	Study-type	Country	Population[a] description, sample size, age range, mean age±SD, % female		
				Design	Pilot	Validation[b]
Belief-Based Reproductive Health Questionnaire (BBRHQ)	Darabi et al. (2018)	DV	Iran	Participants<br>*N*=40	Adolescent girls<br>*N*=10<br>12-15y	Female students<br>*N*=289<br>12-15y, *M*=14.26y±0.96y
Untitled	Edwards et al. (2015)	P	USA	High school youth<br>*N*=218<br>13-18y,<br>*M*=15.56y±1.32y<br>44.5%f, 0.9% other	NA	NA
	Edwards et al. (2018)	DV		NA	NA	Students<br>*N*=3172<br>13-19y, *M*=15.71y±1.17y<br>51.4%f
Untitled	Fisher et al. (2018)	DV	USA	Transgender youth	Transgender youth<br>*N*=11	Transgender youth<br>*N*=228<br>14-21y, *M*=17.86±1.86y<br>45% trans masculine, 41% trans feminine, 14% gender nonbinary
Attitudes Toward Affective-Sexual Diversity Scale (ADAS)	Garrido-Hernansaiz et al. (2018)	DV	Spain	NA	NA	High school students<br>*N*=676<br>13-18y, *M*=14.48y±1.17y<br>49%f*
The Sexual Relationship Power Scale (SRPS) - Subscale	Pulerwitz et al. (2018)	DV	USA (Kenya)	NA	NA	Adolescent girls and young women<br>*N*=1101<br>15-24y, 23.7%=15-17y, 35.4%=18-20y
Untitled	Apidechkul (2019)	DV	Thailand	*N*=12<br>50%f*	*N*=20<br>50%f*	Hill tribe youths<br>*N*=1325<br>15-24y, 58.9%=15-17y, 30%=18-20y<br>60.5%f
Scale of Myths about Sexuality (Escala de Mitos sobre<br>la Sexualidad<br>EMS)	Guerra et al. (2019)	DV	Spain	Adolescents	Adolescents<br>*N*=6	*N*=216<br>*M*=14.19y±1.45y<br>47.22%f<br><br>*N*=661<br>*M*=15.07y±1.20y<br>48.87%f

OMI	Study	Study-type	Country	Population[a] description, sample size, age range, mean age±SD, % female		
				Design	Pilot	Validation[b]
Questionnaire on Sexual and Reproductive Health Literacy (Questionnaire on SRHL)	Vongxay et al. (2019)	DV	Lao PDR Netherlands	NA	High school adolescents<br>*N*=40	High school students<br>*N*=416<br>15-19y, *M*=16.9y±1.0y<br>52,9%f
Adolescent Students' Attitudes Scale towards Sexuality (Escala de Atitudes dos Alunos Adolescentes em face da Sexualidade E3AS)	Barros et al. (2020)	DV	Portugal	NA	Students<br>*N*=20<br>19y or younger	Adolescents<br>*N*=394<br>*M*=14.9y±1.4y<br>46.7%f*
HIV Attitudes Scale (HIV-AS)	Espada et al. (2013)	DV	Spain USA	NA	Students<br>*N*=25	High school students<br>*N*=1216<br>male: *M*=15.87y±0.80y, female: *M*=15.81y±0.76y<br>56.9%f,
	Morales et al. (2019)	TV	Spain	NA	Adaption:<br>Adolescents<br>*N*=23<br>13-19y	Adolescents<br>*N*=1574<br>15-18y, *M*=16.32y±1.02y<br>54%f
	Gómez-Lugo et al. (2020)	TV	Colombia Spain	NA	Cultural adaptation without adolescent population	Students<br>*N*=867<br>14-19y, *M*=15.97y±1.37y<br>52.8%f*
Untitled	Yau et al. (2020)	DV	Thailand	NA	Students<br>*N*=47	Students<br>*N*=423<br>15-19y, *M*=16.79y±1.07y<br>49.9%f
Untitled	Zakaria et al. (2020)	DV	Bangladesh China	NA	Adolescent girl students<br>*N*=60	Older adolescent girls<br>*N*=792, *M*=16.59y±0.49y
HIV and Other STIs Knowledge Scale (KSI)	Abello-Luque et al. (2021)	DV	Colombia Spain	NA	NA	Adolescents<br>*N*=866<br>14-19y, *M*=15.94±1.30y<br>53%f

OMI	Study	Study-type	Country	Population[a] description, sample size, age range, mean age±SD, % female		
				Design	Pilot	Validation[b]
Gender Equitable Attitudes Scale	Yonas et al. (2013)	P	USA	NA	NA	NA
	Hill et al. (2021)	DV		Adolescent boys	A community-based sample of adolescents and young adults	Male participants<br>*N*=866<br>13-19y, *M*=15.50y*±1.64y*<br><br>Boys<br>*N*=698<br>Grade 8-9<br><br>Girls<br>*N*=246<br>13-19y, *M*=15.23y*±1.54y*
Reproductive Health Literacy Questionnaire	Ma et al. (2021)	DV	China	NA	Unmarried youth<br>*N*=20	Students<br>*N*=1587<br>16-25y (60.4%=16-18y<br>34.5%=19-21y<br>5.1%=22-25y)<br>50.2%f
The Sexual and Reproductive Empowerment Scale for Adolescents and Young Adults	Fefferman & Upadhyay (2018)	P	USA	Young men and women<br>*N*=40<br>15-24y	Young men and women<br>*N*=40<br>15-24y, 47%=18-21y<br>50%f*	NA
	Upadhyay & Lipkovich (2020)	P		NA	Adolescents and young adults<br>*N*=30<br>15-26y*, 27%=15-17y, 33%=18-20y<br>60%f, 37% male, 3% transgender	NA
	Upadhyay et al. (2021)	DV		NA	NA	Adolescents and young adults<br>*N*=1117<br>15-24y, 35.8%=15-17y, 17.3%=18-19y<br>49.4%f, 49.3% male, 1.3% transgender

OMI	Study	Study-type	Country	Population[a] description, sample size, age range, mean age±SD, % female		
				**Design**	**Pilot**	**Validation[b]**
Stage of Change (SOC) Measure	Yusufov & Orchowski (2020)	DV	USA	NA	Female Particpants<br>*N*=17	Women at a university<br>*N*=300<br>18-24y, *M*=18.5±0.79y
The Married Adolescent Women's Sexual and Reproductive Health Needs Assessment Questionnaire (MAWSRHNAQ)	Ghiasi et al. (2022)	DV	Iran	Married adolescent women<br>*N*=34	Married adolescent women<br>*N*=10	Married adolescent women<br>*N*=248
The Inventory of Anal Sex Knowledge (iASK)	Kutner et al. (2022)	DV	USA	NA	Adolescent sexual minority, Males<br>N=4<br>16-17y	Adolescent sexual minority, Males<br>*N*=154<br>14-17y, *M*=15.96y±0.85y
The Youth Sexual Intention Scale (YSIS)	Lubis et al. (2022)	DV	Indonesia	Sexually Active Adolescents<br>*N*=30<br>15-18y	NA	Adolescents<br>*N*=396<br>15-18y, *M*=16.2y±0.92y<br>48.74%f
The Condom Use Negotiated Experiences Through Technology (CuNET) Scale	Okumu et al. (2022)	DV	Uganda<br>USA<br>Canada	Adolescents<br>*N*=4<br><br>Peer research assistans<br>*N*=12<br>18-24y	NA	Forcibly displaced adolescents in the slums of Kampala<br>Uganda<br>*N*=242<br>16-19y, *M*=17.56±1.10y<br>81%f
The Masturbation Beliefs Scale (BMS!) – Chinese Version	Ren et al. (2022)	DV	China	information-rich interview subjects	NA	Students<br>*N*=1645<br>16-26y, male: *M*=18.92y±1.1y, female: *M*=18.97y±1.09y<br>29.7%f<br><br>Students<br>*N*=1568<br>16-28y, male: *M*=19.64y±1.21y, female: *M*=19.38y±1.13y<br>76.6%f

OMI	Study	Study-type	Country	Population[a] description, sample size, age range, mean age±SD, % female		
				Design	Pilot	Validation[b]
The Gender Roles and Male Provision Expectations (GRMPE) scale	Kyegombe et al. (2020)	P	USA<br>UK<br>Uganda	Adolescents and young adults<br>*N*=40*<br>14-24y, 30%*=14+y in school, 30%*= 14-17y out of school, 40%*=18-24y out of school<br>55%f*	NA	NA
	Stoebenau et al. (2022)	DV		Young women (Focus Groups)<br>*N*=10<br>15-24y<br><br>Secondary Analysis: Adolescents and young adults<br>*N*=40*<br>14-24y, 30%*=14+y in school, 30%*= 14-17y out of school, 40%*=18-24y out of school<br>55%f*	Women<br>*N*=32<br>15-24y	Adolescent Girls and young women<br>*N*=108, *M*=19.9y
The Attitudes Toward Transactional Sex Scale	St Lawrence et al. (2023)	DV	USA<br>Botswana	Botswana adolescents<br>*N*=40*<br>13-19y, *M*=15.3y<br>50%f*	NA	Public junior and secondary school youth<br>*N*=186<br>13-18y, *M*=NA<br>45%f<br><br>Youth<br>*N*=387<br>13-18y, *M*=15.2y<br>57%f*

OMI	Study	Study-type	Country	Population[a] description, sample size, age range, mean age±SD, % female		
				**Design**	**Pilot**	**Validation[b]**
The Gender Climate Scale (GCS)	Ullman (2014)	P	Australia	Students<br>*N*=5<br>16-19y<br>60%f*<br>20%* transgender male<br>20%* male	NA	NA
	Ullman et al. (2023)	DV		NA	Gender and/or sexually diverse young people<br>*N*=4	Gender and/or sexually diverse Youth<br>*N*=2376<br>13-18y, *M*=15.6<br>58.7%f<br>21.0% male<br>8.9% non-binary<br>7.1% unsure of their gender<br>4.3% another way

Sorted by publication year of the most recent study on the respective OMI; (/*Country*)=Study was conducted in that country but without authors from this country; Type of study: P=preliminary study, D=Development study, T=Translation/Cultural Adaption, V=Validation study; (*Language*)=The OMI language was not explicitly mentioned in the publication itself and was derived from the context of the study; NA=not applicable

[a]Adolescent (sub)groups that where included in the development or validation process, involved experts are not mentioned; when possible description includes: description, sample size, age range, Mean age±SD, y=years, %f=% female

[b]Unless noted otherwise, the baseline population is reported

*calculated by the author (MM) using the published data from the study
