# Supplementary material for: A COSMIN Systematic Review of Sexual Health Literacy Self-Report Measures for Adolescents
Source: Arch Sex Behav. 2025 Jun 6;54(5):1737–68. doi: 10.1007/s10508-025-03142-1 (PMC12162768; doi:10.1007/s10508-025-03142-1)
Supplement: Supplementary file 6 — Supplementary file6 (PDF 124 KB) [file 10508_2025_3142_MOESM6_ESM.pdf]

Fig. 1

Years of publication of studies included in the systematic review of self-reported outcome measurement instruments of sexual health literacy in adolescents

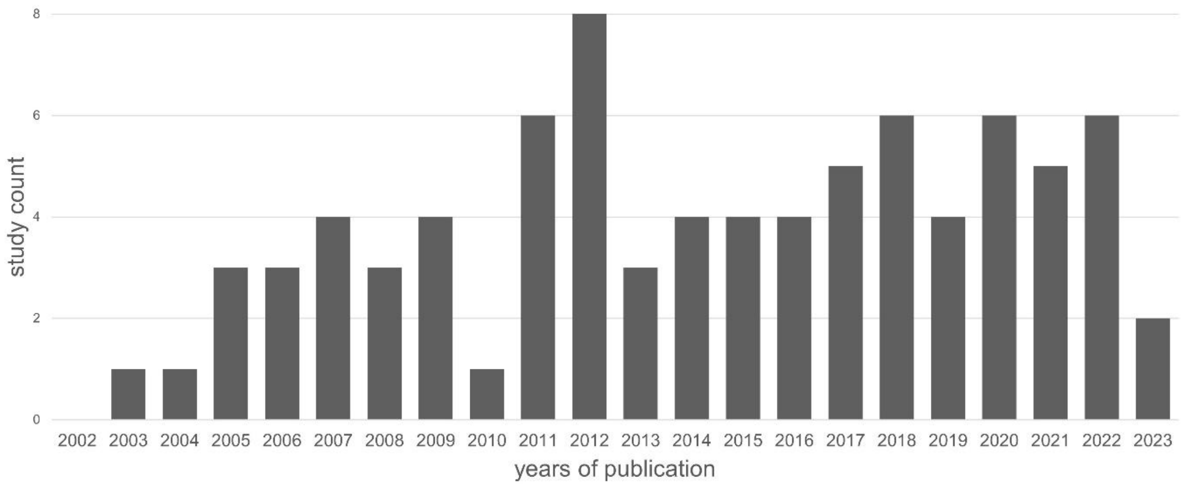

Note. Included studies were published between 2003 and 2023.

Fig. 2

Availability of sample descriptions in the studies included in the systematic review of self-reported outcome measurement instruments of sexual health literacy in adolescents

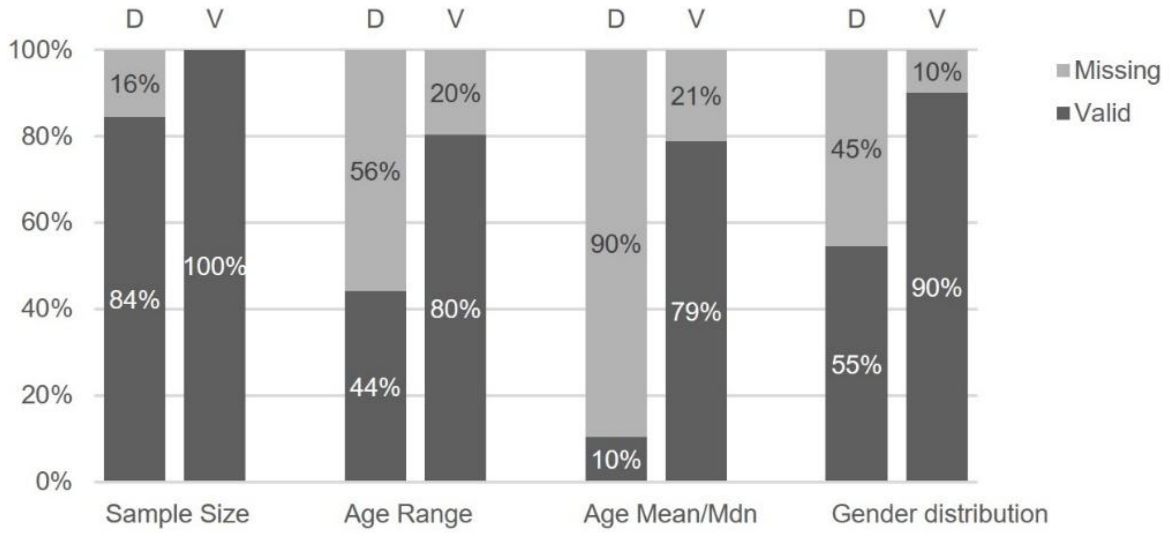

Note. D=Development study (design and pilot phase), V=psychometric validation study
