# Supplementary material for: A COSMIN Systematic Review of Sexual Health Literacy Self-Report Measures for Adolescents
Source: Arch Sex Behav. 2025 Jun 6;54(5):1737–68. doi: 10.1007/s10508-025-03142-1 (PMC12162768; doi:10.1007/s10508-025-03142-1)
Supplement: Supplementary file 7 — Supplementary file7 (PDF 84 KB) [file 10508_2025_3142_MOESM7_ESM.pdf]

Spanish and Portuguese articles

Bello-Villanueva, A. M., Oviedo-Trespalac, O., Vera-Villarroel, P., Oviedo, Ó., Rodriguez-Diaz, M., Celis-Atenas, K., & Pavez, P. (2014). Presentación de una escala para evaluar actitudes y creencias sobre la sexualidad reproductiva en adolescentes varones de la región Caribe colombiana [Presentation of an Attitudes and Beliefs Scale to assess adolescent sexuality and reproductive health of young males in the Caribbean region]. *Universitas Psychologica*, *13*(1), 47–60. https://doi.org/10.11144/Javeriana.UPSY13-1.peea

Bello-Villanueva, A. M., Palacio, J., Vera-Villarroel, P., Oviedo-Trespalacios, O., Rodríguez-Díaz, M. A., Celis-Atenas, K., & Pavez, P. (2016). Construcción y validación de una escala para evaluar salud sexual y reproductiva en adolescentes mujeres de la Región Caribe Colombiana [The development and validation of a scale to measure reproductive and sexual health of young women in the Colombian Caribbean region]. *Universitas Psychologica*, *15*(1), 99–115. https://doi.org/10.1037/t61799-000.

Espada, J. P., Guillén-Riquelme, A., Morales, A., Orgilés, M., & Sierra, J. C. (2014). Validación de una escala de conocimiento sobre el VIH y otras infecciones de transmisión sexual en población adolescente [Validation of an HIV and other sexually transmitted infections knowledge scale in an adolescent population]. *Atencion primaria*, *46*(10), 558–564. https://doi.org/10.1016/j.aprim.2014.03.007

Fernández-Fuertes, A. A., Fuertes, A., & Pulido, R. F. (2006). Evaluación de la violencia en las relaciones de pareja de los adolescentes Validación del Conflict in Adolescent Dating Relationships Inventory (CADRI) - Versión española [Assessment of violence in adolescent couples Validation of the Conflict in Adolescent Dating Relationships Inventory (CADRI) - Spanish version]. *International Journal of Clinical and Health Psychology*, *6*(2), 339–358. https://doi.org/10.1037/t00856-000.

Guerra, C., Del Rio, F. J., Im Morales, & Cabello, F. (2017). Validation of the reduced version for adolescents of the Revised sexual opinion survey. *Revista internacional de andrologia*, *15*(4), 135–140. https://doi.org/10.1016/j.androl.2016.10.006

Lima-Serrano, M., Lima-Rodríguez, J. S., Sáez-Bueno, A., & Cáceres-Rodríguez, B. (2013). Diseño y validación de escalas para medir la actitud adolescente hacia: sexualidad, sustancias adictivas y seguridad vial ¿se relacionan con los comportamientos? [Design and validation of scales to measure adolescent attitudes toward sexuality, addictive substances and road safety. Are they related to behaviour?]. *Anales del sistema sanitario de Navarra*, *36*(2), 203–215. https://doi.org/10.4321/s1137-66272013000200004

Magalhães, E., Oliveira, G. K., Leitão, F., Chaves, S., Capela, S., Nogueira, C., & Martins, C. (2007). Adaptação do 'Inventário de Sexismo Ambivalente' para uma população de estudantes universitários Portugueses [Adaptation of the Ambivalent Sexism Inventory for a population of a Portuguese students]. *Psicologia: Teoria, Investigação E Prática*, *12*(1), 41–54. https://doi.org/10.1037/t00700-000.

Martins, S., Machado, C., Abrunhosa, R., & Manita, C. (2012). Escala de crenças sobre violência sexual (ECVS) [Scale of beliefs about sexual violence (ECVS)]. *Análise Psicológica*, *30*(1-2), 177–191.

Sierra, J. C., Iglesias, P. S., & Monge, F. S. (2013a). Inventory of Negative Attitudes Toward Masturbation: Validity, Reliability and Proposal of a Reduced Version for the Adolescent Population. *Revista Argentina de Clinica Psicologica*, *22*(1), 57–65.

Sierra, J. C., Iglesias, P. S., & Monge, F. S. (2013b). Inventario de Actitudes Negativas Hacia La Masturbación: Validez, Fiabilidad y Propuesta de una Versión Reducida para Población Adolescente [Negative Attitudes Toward Masturbation Inventory: Validity, Reliability, and Proposal of a Reduced Version for Adolescents]. *Revista Argentina De Clinica Psicologica*, *XXII*(I), 57.

Sierra, J. C., Perla, F., & Gutierrez-Quintanilla, R. (2010). Attitudes Toward Masturbation in Adolescents: Psychometric Properties of Spanish Version of Attitudes Toward Masturbation Inventory. *Universitas Psychologica*, *9*(2), 531–542.

Teva, I., & Bermúdez, M. P. (2008). Adaptación castellana y propiedades psicométricas de la escala de búsqueda de sensaciones sexuales en adolescentes Españoles [Psychometric properties of a Spanish adaptation of the Sexual Sensation Seeking Scale in Spanish adolescents]. *Revista Mexicana De Psicología*, *25*(1), 129–137. https://doi.org/10.1037/t04803-000

Vallejo-Medina, P., Saavedra-Roa, A., Gomez-Lugo, M., Morales, A., Abello-Luque, D., Garcia-Montano, E., Garavito-Zamudio, C., & Espada, J. P. (2018). Adaptación, Fiabilidad y Validez de una Versión Breve de la Multicomponent AIDS Phobia Scale (MAPS) en Adolescentes Colombianos [Adaptation, reliability and validity of a Brief Multicomponent AIDS Phobia Scale (MAPS) in Colombian adolescents]. *REVISTA IBEROAMERICANA DE PSICOLOGIA Y SALUD*, *9*(1), 42–57. https://doi.org/10.23923/j.rips.2018.01.014

Vargas, E., & Ponsoda, V. (2010). Escala de actividad sexual: Un instrumento para predecir el inicio temprano de relaciones sexuales [Sexual Activity Scale: A measure to predict the early onset of sexual intercourse]. *Psicología Conductual*, *18*(3), 591–611.

Vega, V. C. (2006). Construccion de un instrumento para la medicion de la conducta sexual en adolescentes femeninas: El CCS [Assessment of a sexual behavior questionnaire for female adolescents: The CCS]. *Interdisciplinaria Revista De Psicología Y Ciencias Afines*, *23*(1), 47–79. https://doi.org/10.1037/t19271-000.

Yago Simón, T., & Tomás Aznar, C. (2013). Condicionantes de género en anticoncepción: diseño y validación de un cuestionario [Gender-determinant factors in contraception: design and validation of a questionnaire]. *Atencion primaria*, *45*(8), 418–425. https://doi.org/10.1016/j.aprim.2013.04.013
